# Frequency and patterns of ribonucleotide incorporation around autonomously replicating sequences in yeast reveal the division of labor of replicative DNA polymerases

**DOI:** 10.1093/nar/gkab801

**Published:** 2021-09-22

**Authors:** Penghao Xu, Francesca Storici

**Affiliations:** School of Biological Sciences, Georgia Institute of Technology, Atlanta, GA 30332, USA; School of Biological Sciences, Georgia Institute of Technology, Atlanta, GA 30332, USA

## Abstract

Ribonucleoside triphosphate (rNTP) incorporation in DNA by DNA polymerases is a frequent phenomenon that results in DNA structural change and genome instability. However, it is unclear whether the rNTP incorporation into DNA follows any specific sequence patterns. We analyzed multiple datasets of ribonucleoside monophosphates (rNMPs) embedded in DNA, generated from three rNMP-sequencing techniques. These rNMP libraries were obtained from *Saccharomyces cerevisiae* cells expressing wild-type or mutant replicative DNA polymerase and ribonuclease H2 genes. We performed computational analyses of rNMP sites around early and late-firing autonomously replicating sequences (ARSs) of the yeast genome, where leading and lagging DNA synthesis starts bidirectionally. We found the preference of rNTP incorporation on the leading strand in wild-type DNA polymerase yeast cells. The leading/lagging-strand ratio of rNTP incorporation changes dramatically within the first 1,000 nucleotides from ARSs, highlighting the Pol δ - Pol ϵ handoff during early leading-strand synthesis. Furthermore, the pattern of rNTP incorporation is markedly distinct between the leading and lagging strands not only in mutant but also in wild-type polymerase cells. Such specific signatures of Pol δ and Pol ϵ provide a new approach to track the labor of these polymerases.

## INTRODUCTION

Ribonucleoside monophosphates (rNMPs), the basic units of RNA, are abundantly embedded in the DNA of many species (reviewed in (1)), leading to DNA structural changes (2), genome instability (3), and disease (4–6). The rNMP patterns in DNA and the biological and clinical significance of rNMPs in genomic DNA still need to be discovered. A proven, major cause for rNMP presence in DNA is rNTP misincorporation by replicative polymerases during the DNA replication process. It has been shown that abundant rNTPs are incorporated during DNA replication by the three replicative DNA polymerases of *Saccharomyces cerevisiae*, DNA Pol α, Pol δ, and Pol ϵ, which have different rNTP incorporation rates *in vitro* in physiological conditions (7, 8). Pol α incorporates one rNTP per 625 nt, Pol δ incorporates one rNTP per 5,000 nt, and Pol ϵ incorporates one rNTP per 1,250 nt on average (8,9). Each replicative DNA polymerase (Pol α, Pol δ, and Pol ϵ) contains a tyrosine in the active site functioning as a steric gate to block the introduction of rNTPs in its catalytic subunit (10). Hence, point mutations at or next to this tyrosine residue (*pol1-Y869A* and *pol1-L868M* for Pol α, *pol2-M644G* for Pol ϵ, *pol3-L612M and pol3-L612G* for Pol δ) lead to a deficiency in the discrimination of rNTPs, and thus cause a higher level of rNTP incorporation (11,12).

The ribonucleotide excision repair (RER) pathway is the principal repair mechanism of rNTP misincorporation, which is initiated by the ribonuclease (RNase) H2 (13). Cells containing a null allele of the catalytic subunit of RNase H2 (*rnh201*-null for yeast and RNase H2A^-/-^ for mammalian cells) cannot perform the RER process, resulting in abundant rNMPs in their genomic DNA (7,14).

**Figure 1.**
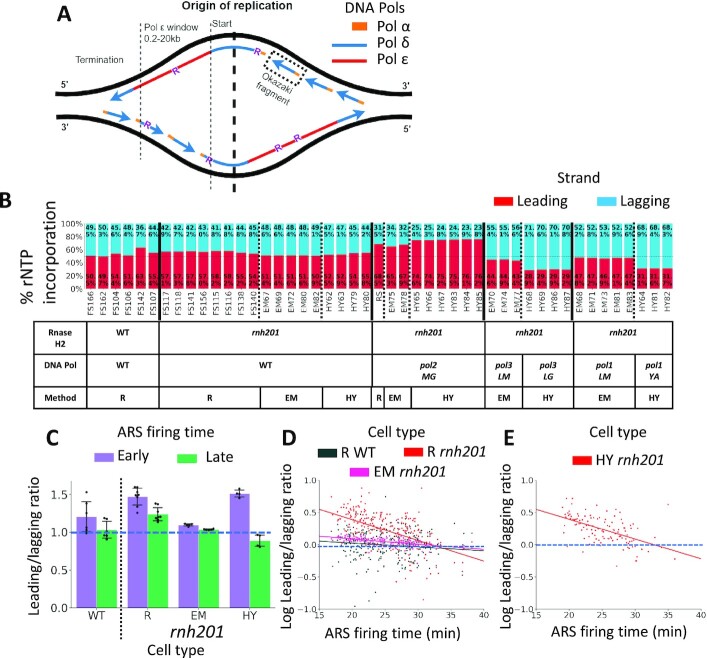
rNTP incorporation is prevalent on the leading strand in the presence of wild-type DNA polymerases α, δ, and ϵ. (**A**) Model for the division of labor of yeast replicative DNA polymerases and rNTP incorporation at the replication fork. DNA replication starts from the ARS, and the leading and lagging strands are synthesized along the ARS flanks. The leading strand synthesis starts upstream of the ARS center with a lagging-strand primer, which is initiated by DNA Pol α and extended by Pol δ. Pol ϵ takes over and synthesizes the bulk of the leading strand afterward. Finally, DNA Pol ϵ hands back to Pol δ when the leading strand meets the incoming lagging strand (12,18). The lagging strand is composed of Okazaki fragments, which are synthesized by Pol α and Pol δ. Abundant rNMPs (R, in purple) are embedded in the newly synthesized strands during the replication process. (**B**) Bar graphs showing the percentage of rNTP incorporation on the leading (red bars) and lagging (blue bars) strands around all confirmed ARSs. The rNMP library name is indicated below each bar. ARS flank length = 15 kb. The table below the bar graphs shows the genotypes of RNase H2 and DNA polymerases, as well as the technique used for the rNMP library preparation. R, ribose-seq libraries; EM, emRiboSeq libraries; HY, RHII-HydEn-seq libraries; *rnh201*, RNase H2 defective mutant; *pol2MG*, *pol2-M644G* mutant; *pol3LM*, *pol3-L612M* mutant; *pol3LG*, *pol3-L612G* mutant; *pol1LM*, *pol1-L868M* mutant; *pol1YA*, *pol1-Y869A* mutant. (**C**) Bar graph showing the mean leading/lagging ratio of rNTP incorporation around early (purple) and late (green) firing ARSs in ribose-seq libraries with wild-type DNA polymerase and wild-type RNase H2 (*N* = 6), and *rnh201*-null ribose-seq (*N* = 8), emRiboSeq (*N* = 5) and RHII-HydEn-seq (*N* = 4) libraries. The thin, dashed line marks a leading/lagging ratio = 1. The error bar represents the 1.5 interquartile range (IQR). (**D**) Scatter plot showing the relation between the log-leading/lagging ratio of rNTP incorporation around ARSs with different firing times in the sacCer2 reference genome. The leading/lagging ratio around each ARS is calculated with maximum likelihood estimation and its logarithm is used to draw the scatter plot. Clear decrease is found in wild-type RNase H2 ribose-seq libraries (*N* = 6, coefficient = -0.0061), *rnh201-*null ribose-seq libraries (*N* = 8, coefficient = -0.0321), and *rnh201-*null emRiboSeq libraries (*N* = 5, coefficient = -0.0082). (**E**) Clear decrease is also found in *rnh201-*null RHII-HydEn-seq libraries (*N* = 4, coefficient = -0.0306) with different ARSs in L03 reference genome. The thin, dashed line marks a leading/lagging ratio = 1.

DNA replication of the leading and lagging strand starts at autonomously replicating sequences (ARSs) in *S. cerevisiae*. The DNA replication starting at ARS proceeds bidirectionally requiring the activity of DNA polymerases α, δ, and ϵ. The lagging-strand synthesis is well known to occur in Okazaki fragments made by Pol α, followed by Pol δ. In contrast, whether leading-strand synthesis is catalyzed by Pol δ and/or Pol ϵ has been subject to ample debate (15,16). Excitingly, recent experiments of replisome reconstitution *in vitro* using yeast purified proteins uncovered that an initial Okazaki fragment synthesized by Pol α and Pol δ on the lagging strand extends on the other side of the origin to prime leading strand synthesis by Pol ϵ (Figure 1A) (17,18). Furthermore, subsequent studies conducted in yeast cells, exploiting leading/lagging biased distribution of rNTP incorporation by a catalytic mutant of Pol α, δ, or ϵ, have provided evidence for the role of yeast DNA Pol δ in initiating leading strand DNA replication (12,19). Nevertheless, taking into consideration that the current work in yeast cells is mainly based on DNA polymerase mutants, which may not accurately represent what happens in cells carrying wild-type DNA polymerases, the prevalence of the model for leading strand synthesis initiated by Pol δ and continued by Pol ϵ needs further confirmation. Besides, there is also another polymerase handoff in the termination zone of the leading strand. When a nascent leading strand meets the incoming lagging strand, the main DNA polymerase is switched from Pol ϵ to Pol δ (12).

With the recent development of rNMP-capture techniques, studies have begun investigating rNTP incorporation in DNA, mainly in yeast *rnh201*-null cells carrying wild-type or mutant alleles of replicative polymerase α, δ, and ϵ (11,12,20–23). All these studies in *rnh201*-null cells revealed more frequent embedding of rNMPs on the leading strand in *S. cerevisiae pol2-M644G* (Pol ϵ mutant) (11,12,21,23) and *Schizosaccharomyces pombe cdc20-M630F* (Pol ϵ mutant) cells consistently (22). Moreover, abundant rNMPs were found on the lagging strand in *S. cerevisiae pol1-L868M, pol1-Y869A*, *pol3-L612M*, and *pol3-L612G* mutant cells (11,12,23).

Here, we conducted a computational study using published datasets from three different rNMP mapping techniques, ribose-seq (21), emRiboSeq (11), and RHII-HydEn-Seq (12), derived from cells of five different yeast strain backgrounds (BY4741, BY4742, YFP17, E134, and Δl(-2)l-7BYUNI300) expressing not only mutant but also wild-type polymerases, containing RNase H2 wild-type or *rnh201-*null alleles to provide new findings highlighting rNMP patterns and the division of labor at the replication fork around *S. cerevisiae* ARSs.

We analyzed rNTP incorporation characteristics in specific regions around early and late firing *S. cerevisiae* ARSs. We found that the rNMP distribution around ARSs displays distinct leading/lagging-strand biases and patterns in different RNase H2 and DNA polymerase genotypes, which are also influenced by the ARS firing time and ARS efficiency.

By studying the rNMP distribution and patterns at different distances from the ARSs, we developed a model of rNTP incorporation rate on the leading and lagging strand and demonstrated the handoff from DNA Pol δ to Pol ϵ at the beginning of the leading strand. Furthermore, we identified unique patterns of rNTP incorporation on the leading and lagging strands, which reflect the rNTP incorporation preference of DNA polymerase Pol δ and Pol ϵ, validating the handoff from DNA Pol δ to Pol ϵ at the beginning of the leading strand synthesis.

## MATERIALS AND METHODS

### Alignment of rNTP incorporation

All libraries in this study were downloaded from NCBI. Library names in this study with corresponding SRR accession and BioProject accession are listed in Supplementary Table S1. For the ribose-seq and emRiboSeq libraries, the locations and counts of rNTP incorporation were identified using the Ribose-Map bioinformatics toolkit and sacCer2 from SGD as the reference genome (24). For the RHII-HydEn-seq libraries, the BigWig files containing the sites of rNTP incorporation based on the L03 reference genome were downloaded from NCBI. We then converted the BigWig files into BED files using bigWigToBedGraph and customized script (12,23).

### ARS region information

For the ribose-seq and emRiboSeq libraries, ARS annotations of OriDB were downloaded and transferred to the sacCer2 reference genome with Liftover software. Only confirmed ARSs were included (*n* = 410) (25). Among them, 276 ARSs have a known firing time (26). They were divided into two halves, early-firing ARSs (*T* ≤ 24.7 min, *n* = 139) and late-firing ARSs (*T* > 24.7 min, *n* = 137). For the RHII-HydEn-seq libraries, a total of 465 ARSs with firing time were used in the study (12). They were also divided into two halves, early-firing ARSs (*T* ≤ 26.7 min, *n* = 233) and late-firing ARSs (*T* > 26.7 min, *n* = 232). ARSs are also divided into two groups based on their efficiency data in the study (12). ARSs with an efficiency ≥0.7 are treated as high-efficiency ARSs, while ARSs with an efficiency ≤0.5 are low-efficiency ARSs. In the sacCer2 reference genome, a total of 357 ARSs has known efficiency. One hundred and fifty-nine of them are high-efficiency ARSs. One hundred and five of them are low-efficiency ARSs. For the RHII-HydEn-seq libraries, the efficiency of all 465 ARSs is known. One hundred and sixty-six of them are high-efficiency ARSs. One hundred and eighty-nine of them are low-efficiency ARSs. All ARSs used in the study are listed in Supplementary Table S3.

### Calculation of the flank of ARSs and binning

The upstream and downstream 15-kb regions are considered as the flanks of an ARS. The 5′-upstream flanks of ARSs for both the Watson and Crick strands correspond to the lagging strand, and the 3′-downstream flanks of ARSs correspond to the leading strand. If two ARSs are close to each other and the distance between them is smaller than 30 kb, the position of the collision point of the corresponding converging replication forks is calculated with their firing times and the average fork moving speed of 1.6 kb/min (27). Their flanks are between the ARSs location and the calculated collision point. We divided each ARS flank into 500-nt bins starting at each ARS location to analyze the variation of rNTP incorporation preference on the leading and lagging strands during the DNA replication process. Hence, each ARS flank has a maximum number of 30 bins.

### Calculation of rNTP incorporation rate

The rNTP incorporation probability per base (PPB) is used as the measurement of the rNTP incorporation rate in ribose-seq, emRiboSeq and RHII-HydEn-seq libraries. The number of rNTP incorporation in a particular bin or ARS flank is divided by the bin size or ARS flank size to get rNMP per base (RPB). Then, this value is further divided by the total number of rNMPs captured in the library to get the PPB value. The PPB value shows the average probability of an rNTP to be incorporated at each base of one bin or ARS flank.

### The ratio of rNTP incorporation on the leading and lagging strands

Using the number of rNMPs embedded on the 15-kb flanks of the leading and lagging strands around ARSs, we calculated the mean value of the leading/lagging ratio. Moreover, an estimated leading/lagging ratio for the line chart is obtained using libraries of the same genotype and prepared by the same rNMP mapping technique by the following assumption and calculation.

For the libraries ${{i\;}} = \;1,2,3, \ldots ,\;{{N}}$ with the same genotype and prepared by the same rNMP mapping technique, let ${X_i}$ and ${Y_i}$ denote the number of rNMPs located in the leading and lagging strand in the *i*th library. Assuming $a$ and $b$ are the rNTP incorporation rates in the leading and lagging strand at each nucleotide, and ${r_i}$ is the rate at which the embedded rNMP could be captured. Letting $L$ denote the length of the reference genome, we have that the number of rNMP sites follows the Poisson distribution.

$$\begin{equation*}{X_i} \sim {\rm Poisson}\left( {{r_i}aL} \right),{\rm{\;}}\quad {Y_i} \sim {\rm Poisson}\left( {{r_i}bL} \right)\end{equation*}$$

Then we can use Maximum likelihood estimation to calculate the rNTP incorporation rates $a$ and $b$.

$$\begin{equation*}{\rm{}}\left( {a,b} \right) = {\rm{\;argma}}{x_{a,b}}\mathop \prod \limits_{i\; = {\rm{\;}}1}^N {\rm Poisson}\left( {{X_i}|{r_i}aL} \right){\rm{\;}}{\rm Poisson}\left( {{Y_i}|{r_i}bL} \right)\end{equation*}$$

Solving the equation, we obtain,

$$\begin{equation*}{{a}} = \frac{{\mathop \sum \nolimits_{i = 1}^N {X_i}}}{{L\mathop \sum \nolimits_{i = 1}^N {r_i}}},\quad b = \frac{{\mathop \sum \nolimits_{i = 1}^N {Y_i}}}{{L\mathop \sum \nolimits_{i = 1}^N {r_i}}}\;\end{equation*}$$

Hence, the estimated average leading/lagging ratio ${\rm{\theta }}$ could be calculated.

$$\begin{equation*}{\rm{\theta }} = \frac{a}{b}{\rm{}} = \frac{{\mathop \sum \nolimits_{i = 1}^N {X_i}}}{{\mathop \sum \nolimits_{i = 1}^N {Y_i}}}\end{equation*}$$

To identify a changing phase for the rNTP incorporation leading/lagging ratio in *rnh201*-null libraries, we calculated the slope of the line from the replication beginning to each 500-nt bin during DNA replication. A changing phase starts at the center of the ARS locus (0 position on the x-axis) and ends when reaches the highest slope.

### Simulation of rNTP incorporation rate during DNA replication

**Figure 2.**
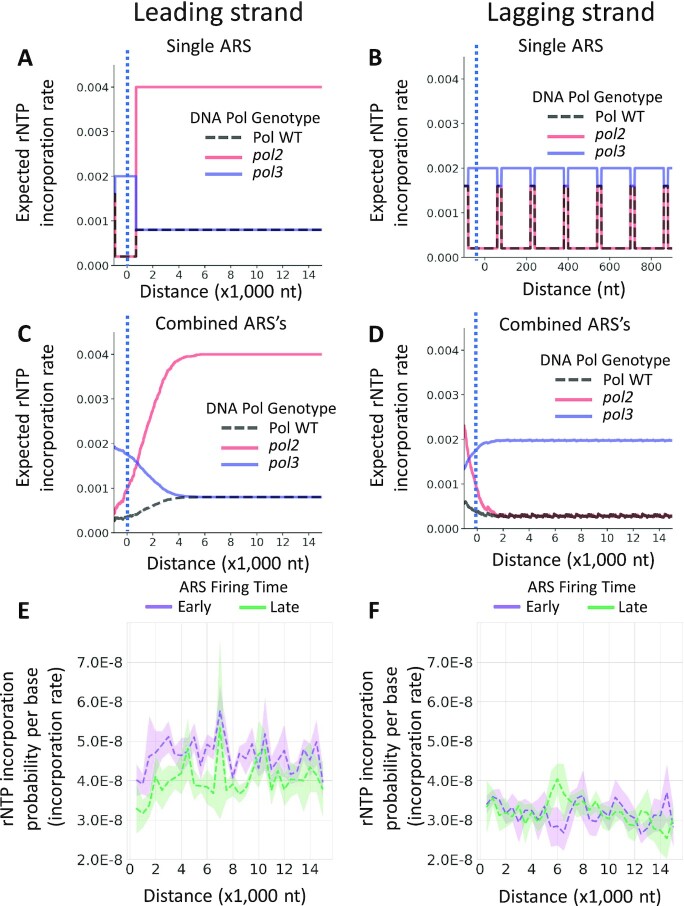
A simulation model for the rate change of rNTP incorporation during DNA replication. (A-D) Simulation results for the rate change of rNTP incorporation starting at the replication origin (See Methods). (**A**) The rNTP incorporation rate on the leading strand of a single ARS. The leading strand synthesis begins with a ‘lagging strand’ primer initiated by DNA Pol α and extended by Pol δ on the upstream of the ARS location (dotted line), which is shown as the leftmost of the X-axis (12,18). The rNTP incorporation rate will change immediately when DNA replication is switched to a different DNA polymerase. Hence, there are 3 phases of the rate induced by DNA Pol α, Pol δ, and Pol ϵ synthesis. Only two phases (Pol δ and Pol ϵ) can be found on the leading strand from the ARS location. (**B**) Simulation result of the rNTP incorporation rate on the lagging strand of a single ARS. The rNTP-incorporation rate changes with the period of the Okazaki fragment (∼200 nt). (**C**) The average rNTP incorporation rate on the leading strands of combined ARSs. When multiple ARSs are considered, the rate is smoothed due to the deviation of the annotated ARS location and the different Pol δ tract lengths. The rate of wild-type Pols and *pol2* mutant increases, and the rate of *pol3* mutant decreases gradually at the beginning. Then all genotypes keep steady. (**D**) The average rNTP incorporation rate on the lagging strand of multiple ARSs. The rate of wild-type Pols and *pol2* mutant decreases, and the rate of *pol3* mutant increases gradually at the beginning. Then all genotypes keep steady. (**E**) Example of an average rNTP incorporation probability per base (PPB) on the leading strand. All ribose-seq *rnh201-*null libraries with wild-type DNA polymerases are used (*N* = 8). The increase of the rNTP incorporation rate at the beginning agrees with the pattern of wild-type DNA polymerase of the leading strand (dashed curve in C). The shadow region represents the standard deviation. (**F**) Example of an average rNTP incorporation probability per base (PPB) on the lagging strand. All ribose-seq *rnh201-*null libraries with wild-type DNA polymerases are used (*N* = 8). The rate decreased slightly at the beginning and then has small variations, which follows the pattern of wild-type DNA polymerase of the lagging strand (dashed curve in D). The shadow region represents the standard deviation.

According to the DNA polymerases division of labor during the DNA replication and the different rNTP incorporation rate of DNA polymerase, we did a simulation (see Data Availability section for the software we used) of rNTP incorporation rate with the leading and lagging strand synthesis. The leading strand synthesis is initiated by DNA Pol α (∼10 nt) at about 100 nt upstream of ARS, followed by a short tract synthesized by DNA Pol δ (on average 160 nt), and then Pol ϵ synthesizes the bulk leading strand (12,18,28). The lagging strand is composed of Okazaki fragments synthesized by DNA Pol α (∼10 nt) and DNA Pol δ (∼160 nt). With the average rNTP incorporation rate of Pol α (1/625 nt), Pol δ (1/5000 nt), and Pol ϵ (1/1250 nt) (8,9) and the higher rNTP incorporation rate of mutant DNA polymerase (mutant Pol ϵ: ∼5-fold of the wild-type Pol ϵ, mutant Pol δ: ∼10-fold of the wild-type Pol δ, estimated from rNMP counts around ARSs in RHII-HydEn-seq libraries) (12), we generated line charts using a custom script simulating the rNTP incorporation rate on the leading and lagging strands around a single ARS as shown in Figure 2A, B. Because the ARS annotation has deviations, and the length of Pol δ tract on the leading strand is different around each ARS, we simulated the average rNTP incorporation rate on the leading and lagging strands of combined ARSs (*n* = 400) with a random deviation of ARS annotations (Normal distribution with standard deviation = 1000 nt), and a random Pol δ-tract length on the leading strand (500-4000 nt) as shown in Figure 2C, D. The parameters including the tract length of each polymerase, rNTP incorporation rate of each polymerase, different number of ARSs, and ARS deviation in the different reference genomes, can change the scale of *x* and *y* axes. But the trend of the single ARS and combined ARSs plot remains the same.

### Preference of rNTP incorporation

The heatmaps showing rNTP incorporation preference were generated as described in (29). Briefly, the four types of rNMPs were counted inside the 4000-10 000-nt, 0-500-nt, 0-200-nt or 0-100-nt window of the ARSs. The dinucleotides composed by the embedded rNMP and its upstream neighbour (NR) or downstream neighbour (RN) were also counted. Each type of rNMP or dinucleotide was normalized based on the background dNMP frequency in the reference genome to obtain the normalized frequency. The background frequencies of the sacCer2 reference genome used by ribose-seq and emRiboSeq, and of the L03 reference genome used by RHII-HydEn-seq are listed in Supplementary Table S2. The frequency was further normalized so that the sum of four types of rNMPs or four dinucleotides with the same rNMP in the same position is 1. Libraries with less than 100 rNMPs embedded in the select range of the ARS are excluded from the composition analysis, and those with less than 400 rNMPs embedded around the ARSs are excluded from the dinucleotide analysis to remove the large variation induced by a limited number of embedded rNMPs. The box plots for dinucleotides (NR) around early and late-firing ARSs with different genotypes were generated with a 1.5 interquartile range (IQR).

### Statistical test

A one-sided Mann-Whitney *U* test was performed to check whether the percentage of rNMPs embedded on the leading strand was significantly higher or lower around early-firing ARSs compared to late-firing ARSs, and to identify preferred rNTP incorporation patterns on the leading and lagging strands within 0-100-nt, 0-200-nt, 0-500-nt or within 4000-10 000-nt windows around early and late-firing ARSs.

## RESULTS

### rNTPs are preferentially incorporated on the leading strand of yeast genomic DNA derived from cells with wild-type DNA polymerases and wild-type RNase H2

We analyzed 15 ribose-seq libraries derived from 4 yeast strain backgrounds: E134, BY4741, BY4742, and YFP17 (21,29), 15 emRiboSeq libraries derived from the Δl(-2)l-7B-YUNI300 background (11), and 17 RHII-HydEn-seq libraries derived from the Δl(-2)l-7B-YUNI300 background (12) to obtain the detailed preference of rNTP incorporation with different RNase H2 and DNA polymerase genotypes (Supplementary Table S1). The rNMP libraries with wild-type Pols were prepared by ribose-seq (*N* = 14), emRiboSeq (*N* = 5), or RHII-HydEn-Seq (*N* = 4) (11,12,29). Six of the 14 ribose-seq libraries of wild-type Pols are also RNase H2 wild-type. All other libraries of wild-type and mutant Pols are *rnh201*-null. Pol ϵ mutant *pol2-M644G* libraries were prepared by ribose-seq (*N* = 1), emRiboSeq (*N* = 2), or RHII-HydEn-Seq (*N* = 6) (11,12,21). Pol δ mutant *pol3-L612M* and *pol3-L612G* libraries were prepared by emRiboSeq (*N* = 3) or RHII-HydEn-Seq (*N* = 4) (11,12). Pol α mutant *pol1-L868M* and *pol1-Y869A* libraries were prepared by emRiboSeq (*N* = 5) or RHII-HydEn-Seq (*N* = 3) (11,12). We utilized 410 confirmed OriDB ARSs in the sacCer2 reference genome for the ribose-seq and emRiboSeq libraries (25) and the 465 ARSs with predicted firing time from the L03 reference genome for RHII-HydEn-seq (12). We counted rNMPs in 15-kb flanks of the ARSs, which cover the initiation and elongation tract of the leading strand synthesis. Most termination zones are not included within these 15-kb flanks of the ARSs (12). Results show the percentages of rNTP incorporation on the leading and lagging strands are different (Figure 1B). Although different yeast strain backgrounds have some variations, there are more rNTPs incorporated on the leading strand in all wild-type DNA polymerase libraries. Among them, the preference is more stable in *rnh201*-null libraries (17 out of 17) than in wild-type RNase H2 libraries (5 out of 6, while 1 library shows no preference), which is likely because the *rnh201*-null libraries have a larger number of incorporated rNTPs (11,12,29).

Since low fidelity mutants of DNA polymerases have a higher probability of misincorporating rNTPs in a strand-biased manner (9), our analysis finds a strong preference on the leading strand of *pol2-M644G* mutant libraries, whose mutant is embedded in DNA Pol ϵ, strengthening previous findings (11,12,20–23). Oppositely, there is a prominent preference on the lagging strand of *pol1* and *pol3* mutant libraries (*pol1-L868M* and *pol3-L612M* for emRiboSeq libraries, *pol1-Y869A* and *pol3-L612G* for RHII-HydEn-seq libraries), whose mutants are in DNA Pol α and Pol δ (Figure 1B). We note that results obtained using RHII-HydEn-seq libraries show stronger strand bias in the mutant Pols. A possible explanation could be the mutants *pol1-Y869A* and *pol3-L612G* have a higher rNTP incorporation rate compared to *pol1-L868M* and *pol3-L612M* used in the emRiboSeq libraries (11,12). The L03 reference genome specifically used for the preparation of these RHII-HydEn-seq libraries may also contribute to the stronger bias.

### The strand preference for rNTP incorporation is stronger around ARSs with an early firing time and high efficiency

The different firing times of ARSs affect the length of the synthesized leading and lagging strand tracts from the ARSs. We wondered whether the firing time of the ARSs would affect the leading/lagging-strand preference of rNMPs that we observed for wild-type RNase H2 and *rnh201*-null cells of wild-type and mutant DNA polymerases. To address this question, we divided the ARSs with known firing times into two halves based on their early or late firing time, as described in the Methods. We then calculated the percentage of rNTP incorporation on the leading and lagging strands for the two groups with all rNMP libraries. In the RNase H2 wild-type and *rnh201*-null of wild-type DNA polymerase libraries, the rNMP preference is on the leading strand, and such preference weakens within the late-firing ARSs compared to the early-firing ARSs (*P* = 0.046, *N* = 6 for RNase H2 wild-type, and *P* = 3.2 × 10^-4^, *N* = 17 for *rnh201*-null libraries, respectively; Supplementary Figure S1). The weakened rNMP preference of the leading strand is also observed in the *pol2-M644G* mutant libraries for the late ARSs compared to the early ARSs (*P* = 2.9 × 10^-4^, *N* = 9, Supplementary Figure S1). As for the *pol1* and *pol3* mutant libraries (*pol1-L868M* and *pol3-L612M* for emRiboSeq libraries, *pol1-Y869A* and *pol3-L612G* for RHII-HydEn-seq libraries), in which the rNMPs are preferentially embedded on the lagging strand, such preference is also less significant around the late-firing ARSs compared to the early-firing ARSs (*pol1*: *P* = 0.042, *N* = 8; *pol3*: *P* = 0.063, *N* = 7, Supplementary Figure S1).

We then calculated the leading/lagging ratio of rNTP incorporation and obtain the mean value to determine the effect of ARS firing time on the preference of rNTP incorporation in wild-type DNA polymerase libraries. Results show a reduced leading/lagging ratio around the late-firing ARSs in both RNase H2 wild type and *rnh201*-null ribose-seq libraries, which suggests that the leading-strand preference is weakened around late-firing ARSs. The *rnh201*-null emRiboSeq libraries show a similar but more slight decrease, and *rnh201*-null RHII-HydEn-seq libraries show a stronger decrease (Figure 1C). To quantitatively determine how the increased firing time reduces the rNTP incorporation preference on the leading or the lagging strand, we used maximum likelihood estimation to calculate the average leading/lagging ratio in each ARS flank. Then we performed linear regression analysis with the log-ratios and the firing time for the ARSs. Despite some variance due to the small number or rNTP incorporation in a single ARS flank, we observed a clear decrease of the log leading/lagging ratio with the increased firing time in *rnh201*-null ribose-seq libraries (coefficient = -0.0321) and RHII-HydEn-seq libraries (Figure 1E, coefficient *=* -0.0306). The decrease of the log leading/lagging ratio is weaker in *rnh201*-null emRiboSeq libraries (Figure 1D, coefficient = -0.0082) but is still stronger than wild-type RNase H2 ribose-seq libraries (Figure 1D, coefficient = -0.0061). In summary, we found that both the leading-strand preference for rNTP incorporation in wild-type DNA Pols and *pol2-M644G* libraries and the lagging-strand preference in *pol1-L868M, pol1-Y869A*, *pol3-L612M*, and *pol3-L612G* libraries are reduced with increasing firing time. And the reduction is stronger in *rnh201*-null libraries compared to wild-type RNase H2 libraries. Although the variation in dNTP pools affects the rNTP incorporation rate (30), the dNTP pools are usually maintained within a certain range to allow genome replication (31). Hence, factors beyond variation in dNTP pools likely affect rNTP incorporation characteristics. A possible explanation for the reduced leading/lagging bias observed around the late-firing ARSs is that the late-firing ARSs tend to have low efficiency and be inconsistently firing. Furthermore, the late-firing ARSs may have a shorter flank length than the 15-kb flank used in the study, which could also reduce the preference.

The firing time of an ARS is directly related to its efficiency. If an early-firing ARS locates near a late-firing ARS, the newly synthesized leading or lagging strand may reach the late-firing ARS location, which inhibits the late ARS firing (12). We divided ARSs with known efficiency into two groups. High-efficiency ARSs have an efficiency ≥0.7. Low-efficiency ARSs have an efficiency ≤0.5. The early-firing ARSs are usually high-efficiency ARSs. And the late-firing ARSs are often low-efficiency ARSs. We studied the incorporated rNTPs around high and low-efficiency ARSs and found a similar conclusion with early and late-firing ARSs. There are more rNTP incorporation on the leading strand in RNase H2 wild-type and *rnh201*-null wild-type DNA Pols and *rnh201*-null *pol2* mutant libraries. There are more rNTPs incorporated on the lagging strand in *rnh201*-null *pol3* mutant libraries and *rnh201*-null *pol1* mutant libraries (Supplementary Figure S2A, B). The preference is stronger around high-efficiency ARSs than low-efficiency ARSs, and such difference is stronger compared to the difference between early and late-firing ARSs. A higher leading/lagging ratio is found around high-efficiency ARSs compared to low-efficiency ARSs in *rnh201*-null ribose-seq, emRiboSeq, and RHII-HydEn-seq libraries (Supplementary Figure S2C). However, due to strong leading strand rNTP incorporation preference in one library (FS142), a higher leading/lagging ratio exists around low-efficiency ARSs in wild-type RNase H2 ribose-seq libraries. Moreover, the firing time of ARSs is usually inversely proportional to ARS efficiency. Therefore, we found a clear increase of the log leading/lagging ratio with the increased ARS efficiency in *rnh201*-null ribose-seq libraries (Supplementary Figure S2D, coefficient = 0.4573) and RHII-HydEn-seq libraries (Supplementary Figure S2E, coefficient = 0.4195). The increase of the log leading/lagging ratio is weaker in *rnh201*-null emRiboSeq libraries (Supplementary Figure S2D, Coefficient = 0.1234). But no increase is found in wild-type RNase H2 ribose-seq libraries (Supplementary Figure S2E, coefficient = -0.0243). In summary, we found that incorporated rNTPs around early-firing ARSs show a similar preference to the ones around high-efficiency ARSs. Also, incorporated rNTPs around late-firing ARSs show a similar preference to the ones around low-efficiency ARSs. The strand preference for rNTP incorporation is stronger around ARSs with an early firing time and high efficiency. This phenomenon is generated by the direct relation between ARS firing time and efficiency.

### DNA Pol δ to Pol ϵ handoff shapes the trend of rNTP incorporation rate on the leading and lagging strands

Using the rNTP incorporation rate for mutant or wild-type DNA Pols, we performed a simulation of rNTP incorporation rate change starting at the replication origin (See Methods). Specifically, the rNTP incorporation rate on the leading strand would follow a high-low-medium pattern from the starting sites. However, the leading strand synthesis initiates upstream of the ARS (18). If we track the rNTP incorporation rate from the ARS, we should start with the Pol δ phase following a low-medium pattern (Figure 2A). On the lagging strand, the rNTP incorporation rate would change with a periodical pattern of Okazaki fragment length (Figure 2B). Furthermore, the average rate change of rNTP incorporation around combined ARSs would be smoothed because there are always deviations in the position of the actual ARSs relative to the annotated ones, and the Pol δ tract length on the leading strand has some variation around different ARSs. With increasing distance from the ARSs, on the leading strand, the rNTP incorporation rate should increase in wild-type DNA polymerase and *pol2-M644G* libraries and decrease in *pol3-L612M* and *pol3-L612G* libraries (Figure 2C). On the lagging strand, the rNTP incorporation rate should decrease in wild-type DNA polymerase and *pol2-M644G* libraries and increase in *pol3-L612M* and *pol3-L612G* libraries (Figure 2D). Although the length of the changing phase may be different when the different parameters (tract length of each polymerase, rNTP incorporation rate of each polymerase, different number of ARSs, and ARS deviation in the different reference genomes, see Methods) are used in the simulation, the increase and the decrease pattern remain unchanged. As a validation, we calculated the average value of rNTP incorporation probability per base (PPB) of wild-type DNA polymerases in *rnh201*-null ribose-seq libraries. On the leading strand, the PPB gradually increases in the first 2,500 nt around early-firing ARSs and the first 4000 nt around late-firing ARSs (Figure 2E). On the lagging strand, after a slight decrease at the beginning, the PPB keeps steady in the DNA replication process. Only small variations are found around early or late-firing ARSs (Figure 2F). These results suggest that the trends of the rNTP incorporation rate during DNA replication are in line with our theoretical model shown in Figure 2C, D.

**Figure 3.**
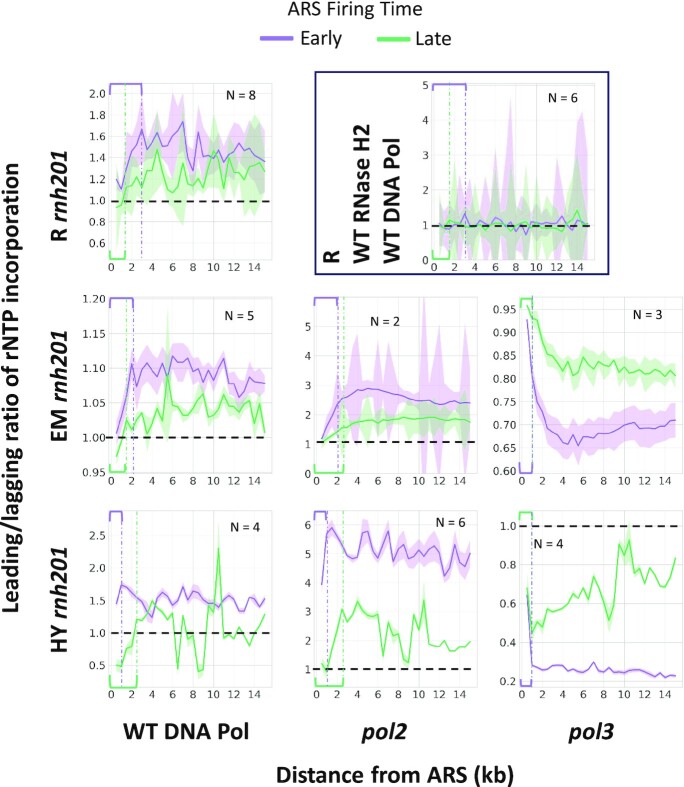
The leading/lagging ratio of rNTP incorporation changes during DNA replication. Maximum likelihood estimation is used to calculate the leading/lagging ratio in RNase H2 wild-type and wild-type DNA polymerase libraries, or *rnh201-*null wild-type DNA polymerase, or mutant polymerase of ribose-seq, emRiboSeq, or RHII-HydEn-seq libraries. The shadow region represents the standard deviation. ARS flank length = 15 kb and bin size = 0.5 kb are used for the plots. The extension of the leading/lagging ratio changing phases (see Materials and Methods) of each panel is indicated by purple (early-firing ARS) and green (late-firing ARS) brackets and dotted-dash lines of the corresponding colors, respectively. R, ribose-seq libraries; EM, emRiboSeq libraries; HY, RHII-HydEn-seq libraries; *rnh201*, RNase H2 defective mutant; *pol2, pol2-M644G* mutant; *pol3, pol3-L612G* mutant for RHII-HydEn-seq libraries and *pol3-L612M* mutant for emRiboSeq libraries. The results presented in this figure are also shown using the same scale in Supplementary Figure S3.

The leading/lagging ratio of rNTP incorporation has the same trend as the leading strand since the rNTP incorporation rate on the lagging strand always changes in the opposite direction, as seen in Figure 2C,D. In wild-type DNA polymerase *rnh201-*null libraries, the leading/lagging ratio of rNTP incorporation increases at the beginning both around early and late-firing ARSs (Figure 3 and Supplementary Figure S3). Since wild-type DNA Pol δ has a lower rNTP incorporation rate than wild-type Pol ϵ, the observed increase suggests that the working polymerase for DNA replication is gradually changing from DNA Pol δ to Pol ϵ. A similar increase is also evident in the wild-type RNase H2 libraries (Figure 3 and Supplementary Figure S3). The increase is weaker since the RER mechanism balances the leading strand preference, and there are more prominent variations after the increase due to the limited number of incorporated rNTPs. A similar but stronger increase occurs in RNase H2 deficient *pol2-M644G* mutant libraries since mutant DNA Pol ϵ has an even higher rNTP incorporation rate than wild-type Pol ϵ (Figure 3 and Supplementary Figure S3). In *pol3-L612M* RHII-HydEn-seq and *pol3-L612G* emRiboSeq libraries, the leading/lagging ratio of rNTP incorporation decreases at the beginning, both for early and late-firing ARSs. This is because mutant Pol δ has a higher rNTP incorporation rate than wild-type Pol ϵ (Figure 3 and Supplementary Figure S3). At the beginning of replication, all libraries have a leading/lagging ratio near 1 compared to the following windows since Pol δ is present on both leading and lagging strands. Overall, these analyses demonstrate that from the ARS center on the leading strand, Pol δ starts DNA synthesis with a handoff to Pol ϵ.

The changing phases of libraries of different DNA polymerases built by different rNMP capture techniques reflect the occurrence of Pol δ to Pol ϵ handoff during the replication beginning, which is indicated by purple (early-firing ARS) and green (late-firing ARS) brackets and dotted-dash lines, respectively (Figure 3). For example, in wild-type DNA polymerase libraries of *rnh201*-null cells, at the beginning of DNA replication when DNA Pol δ takes all synthesis work on both the lagging and the leading strands, the rNTP incorporation rate is the lowest. After the handoff, when DNA Pol ϵ takes the synthesis work on the leading strand, the rNTP incorporation rate is the highest. Thus, the length of the changing phase marks the range of the occurring handoff from Pol δ to Pol ϵ. We find that the changing phase is 2,000-3,000 nt in WT DNA Pols ribose-seq and emRiboSeq libraries and 1,000 nt in the RHII-HydEn-seq libraries (Figure 3).

The same analysis is performed on the rNTP incorporation around the high and low-efficiency ARSs. The results are generally similar to the results around early and late-firing ARSs. The increasing phase is found at the beginning of wild-type Pols and *pol2 rnh201*-null libraries. And the decrease phase locates at the beginning of *pol3 rnh201*-null RHII-HydEn-seq libraries (Supplementary Figure S4). However, we cannot identify a decreased phase in *pol3 rnh201*-null emRiboSeq libraries. A possible reason is that the ARS efficiency in the L03 reference genome does not have a strong correlation to the ARS firing time in the *S. cerevisiae* sacCer2 reference genome. In our study, only 100 out of 139 early-firing ARSs are marked as high-efficiency ARSs, and only 25 out of 137 late-firing ARSs are marked as low-efficiency ARSs in the sacCer2 reference genome. Therefore, ARSs in the sacCer2 reference genome used for emRiboSeq libraries may have different efficiency from those in the L03 reference genome since they have a different location and firing time.

Our analyses of rNMP data around early and late, as well as high and low-efficiency ARSs, show that the leading/lagging ratio of rNTP incorporation is also a better measurement of rNTP incorporation preference compared to the PPB since it is insensitive to rNMP counts compared to the leading-strand and lagging-strand rNTP incorporation rate (Supplementary Figure S5).

### The rNMP composition on the leading and lagging strands is consistent

**Figure 4.**
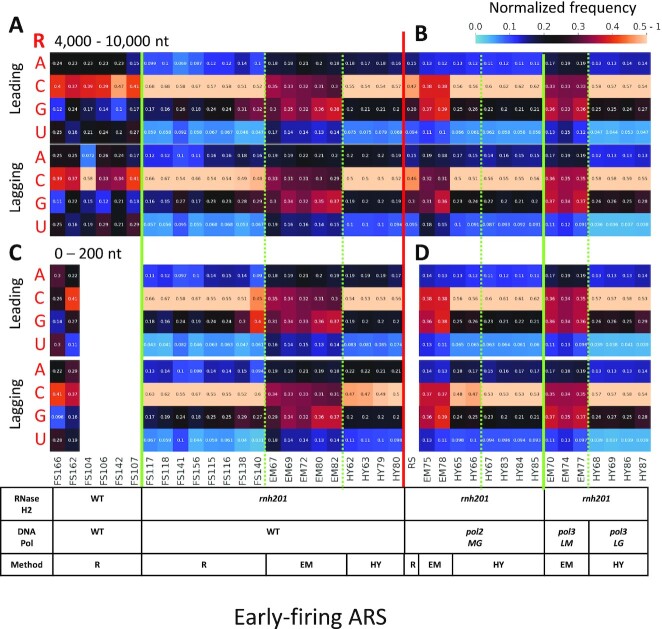
The composition of embedded rNMPs around early-firing ARSs on the leading and lagging strand is the same. Heatmap analyses with the normalized frequency of each type of rNMP (R: rA, rC, rG or rU). The counts for each type of embedded rNMP are normalized to the nucleotide frequencies of the 4000-10 000 nt (top) and 0-200 nt (bottom) windows for the leading or lagging strand around early ARSs from the sacCer2 reference genome for all the ribose-seq and emRiboSeq libraries, and from the L03 reference genome for all the RHII-HydEn-seq libraries. The sum of the four types of rNMP frequency is further normalized to 1. Hence, 0.25 is the expected normalized frequency if there is no rNTP incorporation preference. The corresponding formula used is shown in the Methods section. The background nucleotide frequencies of ribose-seq and emRiboSeq (according to the sacCer2 reference genome), and RHII-HydEn-seq (according to the L03 reference genome) libraries are reported in Supplementary Table S2A and 2B, respectively. Each column of the heatmap shows the results of a specific library. Some libraries with less than 100 rNMPs embedded in the windows were excluded to generate the 0-200-nt window plots. The table underneath the heatmap shows the genotypes of RNase H2 and DNA polymerases, as well as the technique used for the rNMP library preparation. The thick, vertical, red line separates data obtained with wild-type DNA polymerase (**A** for 4000-10 000 nt, **C** for 0-200 nt) from data obtained with mutant DNA polymerases (**B** for 4000-10 000 nt, **D** for 0-200 nt). The thick, vertical, green lines separate data obtained with wild-type RNase H2 from those obtained with *rnh201-*null libraries of wild-type DNA polymerase, and data obtained with different mutant DNA polymerases of *rnh201*-null libraries. The dashed, green lines separate data obtained using different rNMP mapping techniques. Each row shows results obtained for a type of rNMP. The bar to the right shows how normalized frequencies are represented as different colors: black for 0.25; black to yellow for 0.25 to 0.5-1, and black to light blue for 0.25 to 0. R, ribose-seq libraries; EM, emRiboSeq libraries; HY, RHII-HydEn-seq libraries; *rnh201*, RNase H2 defective mutant; *pol2MG, pol2-M644G* mutant; *pol3LM, pol3-L612M* mutant for emRiboSeq libraries; *pol3LG, pol3-L612G* mutant for RHII-HydEn-seq libraries.

We chose two windows for each ARS flanks to check the rNTP incorporation patterns in the leading and lagging strands. The first is a 4000-10 000-nt window. In this window, the leading strand is mainly synthesized by DNA Pol ϵ because the handoffs from Pol δ to Pol ϵ around almost all ARSs are completed at such distance from the ARSs. The other window is the 0-200 nt, which corresponds to the changing phase for all the three rNMP-mapping techniques. In this window, the leading strand is synthesized by both DNA Pol δ and Pol ϵ. For the 4000-10 000-nt window, we analyzed 39 libraries prepared using the ribose-seq, emRiboSeq or RHII-HydEn-seq techniques and derived from 5 different yeast strain backgrounds and 11 different genotypes (Figure 4). In our recent study by Balachander, Gombolay, Yang, Xu et al., 2020 (29), we revealed both conservations in rNMP composition, including low rU in *rnh201*-null strains in nuclear DNA, as well as some variations across different strains of the same *S. cerevisiae* species. Notably, some strains had a very high frequency of rC, while both rC and rG had a high frequency in other strains (29). Like the full double-stranded nuclear genome (29) in *rnh201*-null cells, rU is always the least abundant rNTP incorporated on both the leading and lagging strands around early as well as late-firing ARSs, independently from the genotype of the DNA polymerases (Figure 4A, B, and Supplementary Figure S6A). And rG is the least incorporated rNTP in wild-type RNase H2 cells on both the leading and the lagging strands around early as well as late ARSs (Figure 4A and Supplementary Figure S6A). Moreover, there is no significant difference across the various polymerase alleles in libraries prepared by the three techniques (Figure 4B, Supplementary Figure S6A). We note that for the ribose-seq and RHII-HydEn-seq libraries of *rnh201*-null cells, rC is the most abundant rNMP, while both rC and rG are abundant in emRiboSeq libraries. We also find that different strain backgrounds lead to variation in rNMP composition, as noted before (29). Here, we found that the composition of incorporated rNTPs is consistently similar between the leading and lagging strands around both early (Figure 4A) and late-firing ARSs (Supplementary Figure S6A) in all genotypes and libraries examined within the 4000-10 000-nt window around the ARSs. For the 0-200-nt window, there are fewer rNMPs embedded, and libraries with less than 100 rNMPs embedded in this window were excluded from the analysis. Among the 34 libraries examined, we find that the rNMP composition in the 0-200-nt window is like that in the 4000-10 000-nt window. And no difference is found between leading and lagging strands and around the early or late-firing ARS window (Figure 4C, D, and Supplementary Figure S6A). A similar conclusion exists around high and low-efficiency ARSs, and the termination zone (Supplementary Figure S6B-D).

### The dinucleotide context of rNTP incorporation on the leading and lagging strands are markedly distinct

**Figure 5.**
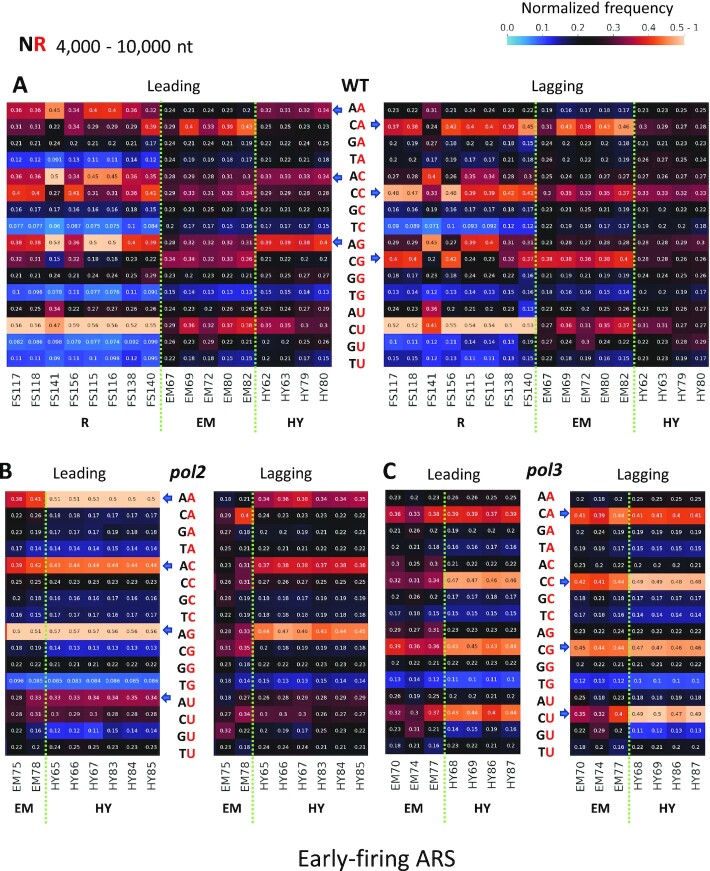
Different dinucleotide NR preferences are revealed on the leading and lagging strands for wild-type and mutant Pols around 4000-10 000 nt of early-firing ARSs in *rnh201-*null libraries. Heatmap analyses with the normalized frequency of dinucleotides composed of the embedded rNMP (R: rA, rC, rG, or rU) and its upstream neighbor (N: dA, dC, dG or dT) (NR) around early-firing ARSs in *rnh201-*null libraries. The counts for each type of dinucleotide are normalized to the dinucleotide frequencies of the 4000-10 000-nt window for the leading or lagging strand around early-firing ARSs in the sacCer2 reference genome for all the ribose-seq and emRiboSeq libraries, and in the L03 reference genome for all the RHII-HydEn-seq libraries. The normalized frequency means the probability of an rNTP to be incorporate in the second position in the dinucleotide. The sum of four normalized frequencies with the same type of incorporated rNTP is further normalized to 1. Hence, 0.25 is the expected normalized frequency if there is no rNTP incorporation preference. The corresponding formula used is shown in the Methods section. The background nucleotide frequencies of ribose-seq and emRiboSeq (according to the sacCer2 reference genome), and RHII-HydEn-seq (according to the L03 reference genome) libraries are reported in Supplementary Table S2A and 2B, respectively. The rNTP incorporation position in the dinucleotide is shown in red. Each column of the heatmap shows the results of a specific library. Each row shows results obtained for a type of rNMP. The preferred patterns are indicated with the blue arrows, and they are different on the leading and lagging strand in *rnh201-*null (**A**) wild-type DNA polymerase ribose-seq, emRiboSeq, and RHII-HydEn-seq libraries, (**B**) *pol2* mutant emRiboSeq and RHII-HydEn-seq libraries, and (**C**) *pol3* mutant emRiboSeq and RHII-HydEn-seq libraries. The color bar on the top right shows how normalized frequencies are represented as different colors: black for 0.25; black to yellow for 0.25 to 0.5-1, and black to light blue for 0.25 to 0. EM, emRiboSeq libraries; HY, RHII-HydEn-seq libraries; *pol2*: *pol2-M644G* mutant libraries, *pol3*: *pol3-L612M* mutant for emRiboSeq libraries, and *pol3-L612G* mutant for RHII-HydEn-seq libraries.

In contrast to the rNMP composition, we uncover that the sequence contexts of embedded rNMPs are different on the leading and lagging strands. For the same 4000-10 000-nt window from the ARSs, we determined the frequency in which each rA, rC, rG or rU is found in a dinucleotide pair with any of the four dNMPs at neighbouring upstream or downstream positions and analyzed such preference. When the upstream dNMP is combined with the embedded rNMP (NR), clear preferences exist around early-firing ARSs in the *rnh201*-null libraries that are also specific on the leading or lagging strands. In particular, dArA and in part dArC and dArG are more frequent on the leading strand than the lagging strand for all *rnh201*-null libraries of wild-type polymerases prepared with all three techniques (Figure 5A, dArA: *P* = 1.82 × 10^-4^, dArC: *P* = 1.07 × 10^-4^, dArG: *P* = 3.46 × 10^-4^). In contrast, dCrA, dCrC, dCrG are more frequent on the lagging strand (Figure 5A, dCrA: *P* = 2.58 × 10^-2^, dCrC: *P* = 7.59 × 10^-3^, dCrG: *P* = 4.66 × 10^-3^). We also examined whether there was any difference in the dinucleotide preference of embedded rNMPs on the leading and lagging strands for the mutant DNA polymerase libraries. In the *pol2*-M644G mutant libraries, the preferred patterns are dArA, dArC, dArG, and dArU, which means the rNMPs tend to be embedded after a dAMP. This pattern is significantly stronger on the leading than on the lagging strand both for emRiboSeq and RHII-HydEn-seq libraries (Figure 5B, dArA: *P* = 2.43 × 10^-4^, dArC: *P* = 1.74 × 10^-4^, dArG: *P* = 1.74 × 10^-4^, dArU: *P* = 7.59 × 10^-3^). Differently, in the Pol δ mutant libraries (*pol3*-*L612M* emRiboSeq and *pol3*-*L612G* RHII-HydEn-seq libraries), the rNMPs tend to be embedded after a dCMP, and this pattern is significantly stronger on the lagging strand (Figure 5C, dCrA: *P* = 1.34 × 10^-3^, dCrC: *P* = 5.51 × 10^-2^, dCrG: *P* = 9.04 × 10^-3^, dCrU: *P* = 8.99 × 10^-2^). Moreover, the pattern for the wild-type DNA polymerase libraries discussed above appears like a combination of the two patterns observed in Pol ϵ and Pol δ mutant libraries. The rNMPs after a dAMP are preferred on the leading strand, and the rNMPs after a dCMP are preferred on the lagging strand (Figure 5A). While the mutant polymerases incorporated more rNTPs due to their reduced sugar discriminations, they also have reduced base selectivity (32). However, the dArN and dCrN patterns are shown in both wild-type and mutant DNA Pols libraries, which suggests that these patterns are not affected by reduced base selectivity (Figure 5).

We performed a similar analysis for dinucleotides (NR) within the 0-200-nt window of the leading and lagging strands. The preferences of rNMP after a dAMP in the leading strand, and rNMP after a dCMP in the lagging strand also exist around early-firing ARSs in the 0-200-nt window (Supplementary Figure S7). However, compared to the 4000-10 000-nt window, the preference is weaker, and the bias of those patterns between the leading strand and lagging strand is also reduced. It is because the 0-200 nt corresponds to the DNA Pol δ to Pol ϵ-handoff phase, resulting in a combined Pol δ and Pol ϵ activity on the leading strand. Such leading and lagging patterns in wild-type polymerases, and Pol ϵ and Pol δ mutants are distinct in the early ARSs, while for late-firing ARSs the difference is maintained, although it is less evident (Supplementary Figure S8A,B). The rNTP incorporation around high-efficiency ARSs has a similar preference to that around early-firing ARSs, while the rNTP incorporation around low-efficiency ARSs is more similar to the one around late-firing ARSs (Supplementary Figure S8C, D).

**Figure 6.**
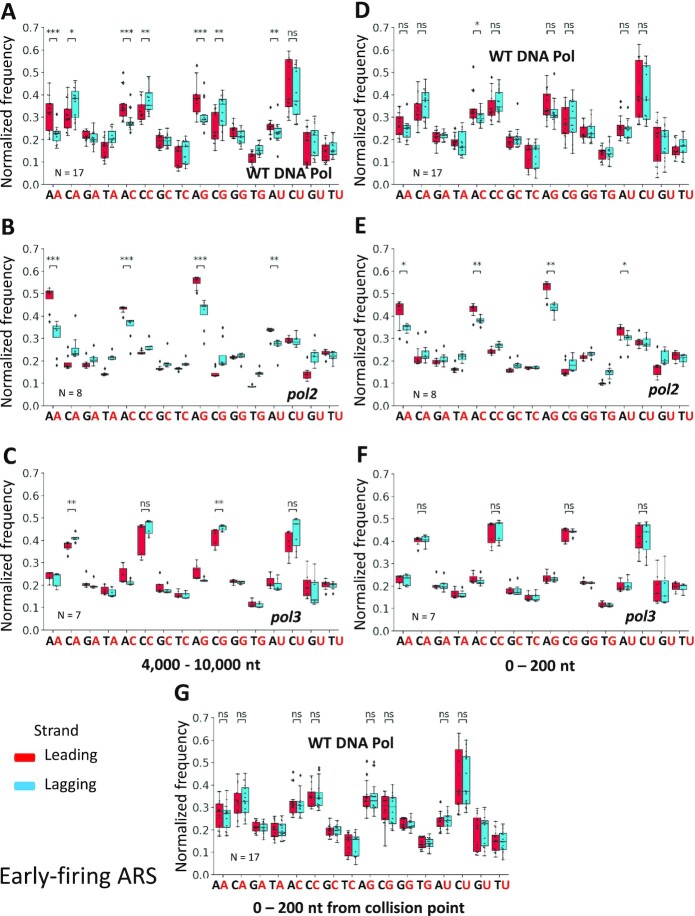
The rNMPs are preferentially preceded by dAMP on the leading strand and dCMP on the lagging strand within the 4000-10 000 nt region around early-firing ARSs in *rnh201*-null libraries. Boxplot of dinucleotide (NR) normalized frequency of each rNMP library around early-firing ARSs. The error bar represents the 1.5 interquartile range (IQR). And the outliers are marked as diamonds. The normalized frequencies of rNTP incorporation in the 4000-10 000-nt window (**A**-**C**), 0-200-nt window (**D**-**F**), and 0-200-nt window from collision point (**G**) on the leading or lagging strands around early-firing ARS are calculated. Normalized dinucleotide (NR) frequencies of wild-type DNA polymerase libraries (*N* = 17) are shown in A, D and G, respectively. Normalized dinucleotide (NR) frequencies of *pol2* mutant libraries, including the *pol2-M644G* mutant of emRiboSeq and RHII-HydEn-seq libraries (*N* = 8) are shown in B and E, respectively. Normalized dinucleotide (NR) frequencies of *pol3* mutant libraries, including *pol3-L612M* mutant of emRiboSeq and *pol3-L612G* mutant of RHII-HydEn-seq libraries (*N* = 7) are shown in C and F, respectively. Mann-Whitney U tests are performed on dinucleotides (NR) with rA in *pol2* mutant libraries (B and E), rC in *pol3* mutant libraries (C and F), and rA or rC embedded in wild-type DNA polymerase libraries (A, D and F). ns: *P*> 0.05, *: 0.05 > *P*> 0.01, **: 0.01 > *P*> 0.001, ***: *P*< 0.001.

To make a better comparison between the two different window lengths, we extracted the normalized frequencies of all 16 dinucleotides (NR) and drew box plots of preferred rNTP incorporation patterns on the leading and lagging strands in wild-type polymerase, Pol ϵ and Pol δ mutant libraries. We find that in wild-type DNA polymerase libraries, all the dArN patterns are significantly preferred on the leading strand, and all dCrN patterns, except dCrU, are significantly preferred on the lagging strand within 4000-10 000-nt window around early-firing ARSs (Figure 6A). However, within the 0-200-nt window, dArC is the only pattern that is significantly preferred on the leading strand, and there is no significantly preferred pattern on the lagging strand (Figure 6D). In Pol ϵ mutant libraries, there are stronger preferences for all dArN patterns, and all preferences are significant within both 4000-10 000 nt and 0-200-nt windows. However, the *P*-values are smaller within the 4000-10 000-nt window (Figure 6B, E). In Pol δ mutant libraries, there are significant dCrA and dCrG preferences in the 4000-10 000-nt window, and no significant preference is present within the 0-200-nt window (Figure 6C,F). These comparisons show that the preferred patterns are more significant within the 4000-10 000-nt window, in which most of the Pol δ to ϵ switches already happened, compared to the 0-200-nt window.

We also performed the same analysis in the 0-500 nt and 0-100-nt windows (Supplementary Figure S9A). Overall, comparing the four windows, we find that the most significant preferences occur in the 4000-10 000-nt window, then 0-500-nt window, then 0-200-nt window, and the 0-100-nt window has the least significant preference of patterns. This phenomenon suggests that there is less leading/lagging strand difference in the windows closer to the ARS, in which less Pol δ to Pol ϵ switch happens, and that there is more Pol δ activity on the leading strand in these windows. The same preferred rNMP patterns are also revealed around the late-firing ARSs, and the difference is more significant in the 4000-10 000-nt window. The rNTP incorporation around high-efficiency ARSs has a similar preference to the ones around early-firing ARSs, while the rNTP incorporation around low-efficiency ARSs is more like the one around late-firing ARSs (Supplementary Figure S9B-G).

We then performed the same analysis with the termination zone (0-200-nt window from the collision points. See Methods.), which is generally like the 0-200-nt window. The two windows keep a similar preference on the leading and lagging strand (Figure 6G, Supplementary Figure S8I). Both also show no significant difference between the leading and lagging strands in wild-type DNA Pols *rnh201*-null libraries. The high similarity between the leading and lagging strands are also shown in the mutant DNA Pol libraries, where the termination zone has only one significantly different pattern (dArC) on the leading and lagging strand in *pol2* mutant libraries and no significance in *pol3* mutant libraries (Supplementary Figure S9H). The similarity of the termination zone to the 0-200-nt window is caused by the fact that the termination zone is the DNA Pol ϵ to Pol δ-handoff phase on the leading strand. And Pol δ synthesizes both leading and lagging strands in the termination zone.

If we combine the rNMPs with their downstream neighbour (RN) in the 4000-10 000-nt window around early-firing ARSs, no clear preference is found in the ribose-seq libraries. The rAdT in wild-type DNA Pols is the only preferred pattern in emRiboSeq libraries. The rNMPs are likely to be embedded before dTMPs in RHII-HydEn-seq libraries. There is also a predominant rAdC pattern around late-firing and low-efficiency ARSs. Those patterns may be caused by a technical factor in the construction of these libraries (Supplementary Figure S10A-D). The same rNdT patterns are also present in the 0-200-nt windows (Supplementary Figure S10E-H) and the termination zone of all confirmed ARSs (Supplementary Figure S10I).

## DISCUSSION

### DNA Pol δ contributes to the beginning of leading strand synthesis in yeast cells with wild-type or mutant DNA polymerases

The function of Pol δ around the replication origin on the leading strand was previously observed from work in yeast (19,33), and from *in vitro* work (18). Our study analyzed the embedded rNMPs on the leading and lagging strands around ARSs in 47 libraries of different genotypes and yeast strains prepared by three rNMP-mapping techniques. We identified the Pol δ to Pol ϵ handoff as changing phase in our study, which is 0 to 3000 nt in ribose-seq, 0 to 2000 nt in emRiboSeq libraries, and 0 to 1000 nt in RHII-HydEn-seq libraries of wild-type Pols and *rnh201*-null cells (Figure 3). This changing phase suggests that the working DNA polymerases are gradually switched from Pol δ to Pol ϵ at the beginning of DNA replication, not only in libraries with the low-fidelity mutant DNA polymerases as previously described (12,19) but also in the wild-type DNA polymerase libraries. The RHII-HydEn-seq data maintain a short-changing phase, which suggests that the ARS annotation in the L03 reference genome is more accurate than the OriDB annotation in the sacCer2 reference genome used by ribose-seq and emRiboSeq data. The same genome identity between the RHII-HydEn-seq libraries and the L03 reference likely contributes to the more accurate results as well. We also found that the Pol δ to Pol ϵ handoff has a longer extension around the late-firing ARSs compared to the early ones in *pol2* mutant libraries. Our interpretation of these results is that the late-firing ARSs usually have low efficiency and are inconsistently firing. Hence, the late ARSs are more difficult to be annotated and their locations have a larger deviation. Moreover, in the wild-type DNA polymerase and *pol2-M644G* mutant RHII-HydEn-seq libraries, the leading/lagging ratio shows a slight decrease at the beginning around late-firing ARSs, which may be related to the Pol α to Pol δ switch.

### Different DNA polymerase usage leads to different dinucleotide preference patterns on the leading and lagging strands

The rNTP incorporation in DNA sequences around the ARSs is caused by different replicative DNA polymerases. Our study demonstrates that while the composition of rNMPs in *rnh201*-null cells is strikingly similar on the leading and lagging strands around ARSs in libraries with wild-type or different DNA Pol δ and Pol ϵ alleles, the dinucleotide preference of rNTP incorporation markedly changes on the leading and lagging strands at different distances from the ARSs and in different DNA polymerase alleles. First, the dinucleotide preference in the 4000-10 000-nt window on the leading strand is different from the preference observed in the same window on the lagging strand. In this window, the leading strand is mainly synthesized by Pol ϵ, and the lagging strand is mainly synthesized by Pol δ. We discovered that DNA Pol ϵ tends to incorporate rNTPs after a dAMP, while DNA Pol δ tends to incorporate rNTPs after a dCMP in the 4000-10 000-nt window (Figure 5, Supplementary Figure S8A-D). These patterns are highly conserved among all the libraries with wild-type or low-fidelity mutant DNA polymerases prepared using the different rNMP-capture techniques. The frequency of the preferred dinucleotide patterns is also different on the leading and lagging strands due to the different DNA polymerase usage. In the wild-type polymerase libraries, we found a combination of the two patterns observed in Pol ϵ and Pol δ mutant libraries. However, the dArN pattern of Pol ϵ is stronger on the leading strand since Pol ϵ synthesizes the main part of the leading strand, and the dCrN pattern of Pol δ is stronger in the lagging strand since Pol δ synthesizes the main part of lagging strand. These dArN-leading and dCrN-lagging patterns are particularly accentuated in the Pol ϵ and Pol δ low fidelity mutant libraries, respectively (Figure 5B, C). Therefore, the specific leading and lagging strand dinucleotide patterns of rNTP incorporation that we found in the 4000-10 000-nt windows from the ARSs reveal unique signatures of Pol δ and Pol ϵ. The patterns also provide a new approach to track the labor of these polymerases not only in yeast but possibly in other eukaryotic cells, like human cells.

Second, we found that the leading/lagging strand differences of the preferred patterns show a progressive reduction of the significance with the shortening distance to the ARSs, as observed in the four different windows, 4000-10 000-nt, 0-500-nt, 0-200-nt, and 0-100-nt on the leading and lagging strands. Such markedly reduced distinction of the leading and lagging dinucleotide patterns (NR) with proximity to the ARSs is evident not only with the mutant but also with the wild-type Pol libraries. These results suggest that the DNA polymerase usage on the leading and lagging strands is more similar in the windows that are closer to the ARSs location (Figure 6A-F and Supplementary Figure S9A-G). The similar DNA polymerase usage in the windows close to the ARSs reveals the Pol δ activity on the leading strand, which is reduced with the occurrence of the Pol δ to Pol ϵ handoff.

### DNA Pol δ contributes to the synthesis of termination zone on the leading strand

Another handoff from DNA Pol δ to Pol ϵ happens in the termination zone on the leading strand before it collides with the incoming lagging strand. Hence, Pol δ also contributes to the leading strand synthesis around the collision points (12). We analyzed the rNTP incorporation dinucleotide preference in the 0-200-nt window from the collision points. The results show a similar preferred pattern as the 4000-10 000-nt window and 0-200-nt window from ARS. The rNMPs are likely to be embedded after dAMP in *pol2* mutant libraries, and after dCMP in *pol3* mutant libraries (Supplementary Figure S8I). However, the preferred patterns and their normalized frequency are similar on the leading and lagging strand (Figure 6G, Supplementary Figure S9H). Since DNA Pol δ synthesizes the majority part of the lagging strand, this similarity suggests that Pol δ also contributes to the termination zone synthesis on the leading strand. Moreover, our results revealed that Pol δ contributes to the termination zones of leading strand synthesis not only in the libraries with low-fidelity DNA polymerases but also in those with wild-type DNA polymerases.

### No rNTP incorporation preference is found between early and late-firing ARSs

Although the majority of ARSs are embedded in the euchromatin regions, each heterochromatin region (HML, HMR, rDNA and sub-telomeric regions) contains at least one ARS (34). There is a total of 52 ARSs in the heterochromatin region; of these, 9 have known firing time in sacCer2 reference genome, and are late-firing ARSs (Supplementary Table S3). There are no ARSs in the centromeres (CENs). However, some ARSs are located near the centromeres (CEN2 & ARS208, CEN3 & ARS308, CEN9 & ARS920 and CEN12 & ARS1208). Three of them are classified as early-firing ARSs and one of them (ARS308) does not have an annotated firing time in the sacCer2 reference genome. The late-firing ARSs may have some relation to the heterochromatin. However, we do not observe a preference for rNTP incorporation in heterochromatin or centromeres. For some late-firing ARSs, there are abundant rNTPs incorporated around. As an example, considering that the late-firing ARS316 located at chrIII: 271 867, there are many rNTPs incorporated around it on both strands in library FS156. These regions with abundant embedded rNMPs can be prone to breakage by RNase H2. There are also some early-firing ARSs that have a high level of rNTP incorporation. Therefore, regions with abundant rNMPs can locate around both early and late-firing ARSs.

### Comparison of the libraries built by using different rNMP capture techniques.

Ribose-seq, emRiboSeq, and RHII-HydEn-seq are three rNMP capture techniques to locate the rNTP incorporation position at single-nucleotide resolution in a reference genome. The characteristics of rNMPs captured by these three techniques are usually similar. As discussed in Balachander *et al.*, emRiboSeq and ribose-seq libraries show a consensus rNTP incorporation pattern in budding yeast cells (29). In our study, we found that rUMP is always the least represented rNMP in all *rnh201*-null libraries prepared using each of these techniques regardless of the polymerase genotype. rCMP and rGMP are the most represented rNMPs across all libraries with *rnh201*-null genotype, independently from the polymerase genotype (Figure 4).

There are also some variations in the predominance of rCMP and rGMP across the emRiboSeq libraries and those of ribose-seq and RHII-HydEn-seq for the *rnh201*-null genotype. We believe that such variation in part derives from the different strain backgrounds of the libraries (ribose-seq versus emRiboSeq). The RHII-HydEn-seq data share the same strain background as emRiboSeq (Δl(-2)l-7BYUNI300). However, the RHII-HydEn-seq data have been generated using a different reference genome from the one used to generate the emRiboSeq data, and this may also affect in part the rNMP composition. Moreover, because the yeast cells used to prepare the ribose-seq, emRiboSeq and RHII-HydEn-seq libraries were cultured in slightly different conditions, the growth conditions may in part contribute to differences in rNMP composition and patterns observed across the different strains for the same genotypes (see column Growth Phase in Supplementary Table S1). In addition, the difference of rNMP-mapping techniques may also contribute to the variation. The three techniques ribose-seq, emRiboSeq, and RHII-HydEn-seq utilize different procedures and enzymes to build the rNMP libraries; thus, these rNMP mapping techniques have different capacities to capture rNMPs. For example, ribose-seq is the only method to directly capture rNMPs without capturing nicks in DNA and RNA primers of Okazaki fragments. The emRiboSeq technique captures the upstream sequence of the incorporated rNTPs and it may capture nicks in DNA. RHII-HydEn-seq technique captures the rNMP terminated sequence and it may capture RNA primers of Okazaki fragments.

Recently a new rNMP-mapping technique was developed, RiSQ-seq that uses RNase HI and HII to cleave at rNMPs embedded in DNA and capture the 5′ dNMP neighbour of the embedded rNMPs (35). While no significant leading strand preference was revealed in wild-type RNase H2 libraries, in line with ours and previous studies, *rnh201-*null libraries showed enrichment of rNTP incorporation on the leading strand (35). Moreover, Iida *et al.* found that the rNMP composition has a higher G/C preference on the leading strand (35). In our data, we also find that the frequency of rGMP and rCMP is slightly higher on the leading strand, but it is not significant (Figure 4A). Potential reasons for these differences are: (i) in our study, we use six rNMP libraries of WT RNase H2 from four yeast strains prepared using the ribose-seq technique, and 17 *rnh201*-null libraries from five strains using three different rNMP mapping techniques; Iida *et al.* use four WT RNase H2 libraries and four *rnh201*-null libraries, all from one strain, BY4741. (ii) The ARSs are used to define the leading and lagging strands as well as the replication start and termination zones in our study. In contrast, Iida *et al.* use fragment coverage of Okazaki fragment sequencing data to only determine the replication direction within gene sequences (35).

In this study, we mainly focus on the common characteristics of rNMP libraries sharing the same genotype and prepared using different techniques and different yeast strains. We analyzed the rNTP incorporation compositions and patterns at different distances from the ARSs. We aimed to find the general rNTP incorporation characteristics around ARSs and understand their relation to the replicative DNA polymerases Pol δ and Pol ϵ. Our results show that libraries built using the three techniques, ribose-seq, emRiboSeq and RHII-HydEn-Seq share similar Pol δ and Pol ϵ handoff and rNTP incorporation dinucleotide patterns—Pol ϵ prefers to incorporate rNTP after a dCMP and Pol δ prefers to incorporate rNTP after a dAMP.

## DATA AVAILABILITY

The *ribose-seq*, emRiboSeq, and RHII-HydEn-seq datasets used in this study are available under NCBI BioProject accession PRJNA613920, PRJNA271170, and PRJNA517710. The *pol2* mutant *ribose-seq* dataset is available under NCBI BioProject accession PRJNA261234.

The liftover software, bigWigToBedGraph software, and the chain file from sacCer1 to sacCer2 were downloaded from UCSC Genome Browser Utilities (http://hgdownload.soe.ucsc.edu/downloads.html).

The Ribose-Map bioinformatics toolkit is available on GitHub at https://github.com/agombolay/ribose-map. The RibosePreferenceAnalysis tools used to generate the heatmaps are available on GitHub at https://github.com/xph9876/RibosePreferenceAnalysis. Other scripts for rNMP calculation, plotting, and rNTP incorporation rate-change simulation are available on GitHub at https://github.com/xph9876/rNMP_ARS_analysis.

## Supplementary Material

gkab801_Supplemental_Files
