# Supplementary material for: Frequency and patterns of ribonucleotide incorporation around autonomously replicating sequences in yeast reveal the division of labor of replicative DNA polymerases
Source: Nucleic Acids Res. 2021 Sep 22;49(18):10542–57. doi: 10.1093/nar/gkab801 (PMC8501979; doi:10.1093/nar/gkab801)
Supplement: gkab801_Supplemental_Files [file gkab801_supplemental_files.zip › Supplementary Figures with legends_20210905.pdf]

Supplementary Table 1. All rNMP libraries used in the study. All the libraries used in the study with their SRR Accession and BioProject Accession are listed. For the ribose-seq and emRiboSeq libraries, FASTQ files are downloaded, and Ribose-Map software is used to locate the rNTP incorporation in the SGD sacCer2 reference genome. For the RHII-HydEn-seq libraries, aligned BigWig files with the L03 reference genome are used and converted into BED format.

Supplementary Table 2. Background frequency of heatmaps in the *S. cerevisiae* sacCer2 and the L03 reference genome. Count and frequencies (%) of mononucleotides and dinucleotides of the 4,000-10,000 nt and 0-200-nt windows of the leading and lagging strands around early and late-firing ARSs in (**A**) sacCer2 and (**B**) L03 reference genome. The raw number of each dNMP or dinucleotide in the reference genome is counted, and the percentage is calculated. For the dinucleotide tables, "R" means embedded ribonucleotide, "N" means deoxyribonucleotide neighbor of base A, C, G, or T. The percentage is calculated by dividing the raw number of NR or RN by the sum of the four combinations for each fixed R, e.g., in the NR = AC, the percentage is ACx100/(AC+CC+GC+TC); for RN = AC, the percentage is ACx100/(AA+AC+AG+AT).

Supplementary Table 3. ARSs used in the study. All ARSs used in the study, including all confirmed ARS in SacCer2 reference genome (25), ARSs with firing time in SacCer2 reference genome (26), and ARSs in the L03 reference genome (12) are listed. The firing time group and efficiency group of ARSs, and their special chromatin features are also indicated in columns.

Supplementary Figure 1. rNTP incorporation is prevalent on the leading strand around early and late firing ARSs. Bar graphs showing the percentage of rNTP incorporation on the leading (red bars) and lagging (blue bars) strands around (**A**) early and (**B**) late-firing ARSs. All the libraries with the same RNase H2 and DNA Pols genotype and generated by the same rNMP-capture techniques are combined to a single bar. The error bar shows the standard deviation. The black vertical line separates different RNase H2 and DNA Pols genotypes. The dashed black horizontal line shows where the leading percentage is 50%, which means the same amount of rNMPs embedded in the leading and lagging strands. ARS flank length = 15 kb. All confirmed ARSs in OriDB are used for ribose-seq and emRiboSeq libraries, while confirmed ARSs in the L03 reference genome are used for RHII-HydEn-seq libraries (see Methods). R, ribose-seq libraries; EM, emRiboSeq libraries; HY, RHII-HydEn-seq libraries; *rnh201*, RNase H2 defective mutant; *pol2MG*, *pol2-M644G* mutant; *pol3LM:*, *pol3-L612M* mutant; *pol3LG*, *pol3-L612G* mutant; *pol1LM*, *pol1-L868M* mutant; *pol1YA*, *pol1-Y869A* mutant.

Supplementary Figure 2. rNTP incorporation is prevalent on the leading strand around high and low-efficiency ARSs in the presence of wild-type DNA polymerases α, δ and ε. (**A, B**) Bar graphs showing the percentage of rNTP incorporation on the leading (red bars) and lagging (blue bars) strands around (**A**) early and (**B**) late-firing ARSs. All the libraries with the same RNase H2 and DNA Pols genotype and generated by the same rNMP-capture techniques are combined to a single bar. The error bar shows the standard deviation. The black vertical line separates different RNase H2 and DNA Pols genotypes. The dashed black horizontal line shows where the leading percentage is 50%, which means the same amount of rNMPs embedded in the leading and lagging strands. ARS flank length = 15 kb. All confirmed ARSs in OriDB are used for ribose-seq and emRiboSeq libraries, while confirmed ARSs in the L03 reference genome are used for RHII-HydEn-seq libraries (see Methods). R, ribose-seq libraries; EM, emRiboSeq libraries; HY, RHII-HydEn-seq libraries; *rnh201*, RNase H2 defective mutant; *pol2MG*, *pol2-M644G* mutant; *pol3LM*, *pol3-L612M* mutant; *pol3LG*, *pol3-L612G* mutant; *pol1LM*, *pol1-L868M* mutant; *pol1YA*, *pol1-Y869A* mutant. (**C**) Bar graph showing the mean leading/lagging ratio of rNTP incorporation around high (purple) and low (green) efficiency ARSs in ribose-seq libraries with wild-type DNA polymerase and wild-type RNase H2 (N = 6), and *rnh201*-null ribose-seq (N = 8), emRiboSeq (N = 5), and RHII-HydEn-seq (N = 4) libraries. The thin, dashed line marks a leading/lagging ratio = 1. The error bar represents the 1.5 interquartile range

(IQR). (**D**) Scatter plot showing the relation between the log-leading/lagging ratio of rNTP incorporation around ARSs with different efficiency in the sacCer2 reference genome. The leading/lagging ratio around each ARS is calculated with maximum likelihood estimation and its logarithm is used to draw the scatter plot. Clear increase is found in *rnh201*-null ribose-seq libraries (N = 8, Coefficient = 0.4573), and *rnh201*-null emRiboSeq libraries (N = 5, Coefficient = 0.1234), but not in wild-type RNase H2 ribose-seq libraries (N = 6, Coefficient = -0.0243). (**E**) Clear increase is also found in *rnh201*-null RHII-HydEn-seq libraries (N = 4, Coefficient = 0.4195) with different ARSs in L03 reference genome. The thin, dashed line marks a leading/lagging ratio = 1.

Supplementary Figure 3. The leading/lagging ratio of rNTP incorporation changes during DNA replications on the same scale. The same plots as those shown in **Figure 3**. Here, all plots are on the same scale.

Supplementary Figure 4. The leading/lagging ratio of rNTP incorporation changes during DNA replication around high and low-efficiency ARSs. Maximum likelihood estimation is used to calculate the leading/lagging ratio in RNase H2 wild-type and wild-type DNA polymerase libraries, or *rnh201*-null wild-type DNA polymerase, or mutant polymerase of ribose-seq, emRiboSeq, or RHII-HydEn-seq libraries. The shadow region represents the standard deviation. The extension of the leading/lagging ratio changing phases (see Methods) of each panel is indicated by purple (high-efficiency ARS) and green (low-efficiency ARS) brackets with dotted-dash lines of the corresponding colors, respectively. ARS flank length = 15 kb and bin size = 0.5 kb are used for the plots. R, ribose-seq libraries; EM, emRiboSeq libraries; HY, RHII-HydEn-seq libraries; *rnh201*, RNase H2 defective mutant; *pol2, pol2-M644G* mutant; *pol3, pol3-L612G* mutant for RHII-HydEn-seq libraries and *pol3-L612M* mutant for emRiboSeq libraries.

Supplementary Figure 5. The rNTP incorporation probability per base (PPB) on the leading and lagging strands. The rNTP incorporation probability per base (PPB) on the leading (**A, C**) and lagging (**B, D**) strands in RNase H2 wild-type and wild-type DNA polymerase libraries, or *rnh201*-null wild-type DNA polymerase, or mutant polymerase of ribose-seq, emRiboSeq or RHII-HydEn-seq libraries. ARSs with different firing times are separated in (**A, B**). ARSs with different efficiency are separated in (**C, D**). The shadow region shows the standard deviation at each position. ARS flank length = 15,000 nt and bin size = 500 nt are used for the plots. R, ribose-seq libraries; EM, emRiboSeq libraries; HY, RHII-HydEn-seq libraries; *rnh201*, RNase H2 defective mutant; *pol2, pol2-M644G* mutant; *pol3, pol3-L612G* mutant for RHII-HydEn-seq libraries and *pol3-L612M* mutant for emRiboSeq libraries.

Supplementary Figure 6. The composition of embedded rNMPs around early-firing ARSs on the leading and lagging strand is the same. Heatmap analyses with the normalized frequency of each type of rNMP (R: rA, rC, rG, or rU). The counts for each type of embedded rNMP are normalized to the nucleotide frequencies of the 4,000-10,000 nt (top) and 0-200 nt (bottom) windows for the leading or lagging strand around (**A**) late-firing ARSs, (**B**) high-efficiency ARSs, and (**C**) low-efficiency ARSs from the sacCer2 reference genome for all the ribose-seq and emRiboSeq libraries, and from the L03 reference genome for all the RHII-HydEn-seq libraries. Similar analysis with rNMPs embedded in the 0-200 nt region from anticipated collision points of all confirmed ARSs is shown in (**D**). The sum of the 4 types of rNMP frequency is further normalized to 1. Hence, 0.25 is the expected normalized frequency if there is no rNTP incorporation preference. The corresponding formula used is shown in the Methods section. The background nucleotide frequencies of ribose-seq and emRiboSeq (according to the sacCer2 reference genome), and RHII-HydEn-seq (according to the L03 reference genome) libraries are reported in **Supplementary Table 2A** and **2B**, respectively. Each column of the heatmap shows the results of a specific library. Some libraries with less than 100 rNMPs embedded in the windows were excluded to generate the 0-200-nt window plots. The table underneath the heatmap shows the genotypes of RNase H2 and DNA polymerases, as well as the technique used for

the rNMP library preparation. The thick, vertical, red line separates data obtained with wild-type DNA polymerase from data obtained with mutant DNA polymerases. The thick, vertical, green lines separate data obtained with wild-type RNase H2 from those obtained with *rnh201*-null libraries of wild-type DNA polymerase, and data obtained with different mutant DNA polymerases of *rnh201*-null libraries. The dashed, green lines separate data obtained using different rNMP mapping techniques. Each row shows results obtained for a type of rNMP. The bar to the right shows how normalized frequencies are represented as different colors: black for 0.25; black to yellow for 0.25 to 0.5 – 1, and black to light blue for 0.25 to 0. R, ribose-seq libraries; EM, emRiboSeq libraries; HY, RHII-HydEn-seq libraries; *rnh201*, RNase H2 defective mutant; *pol2MG, pol2-M644G* mutant; *pol3LM, pol3-L612M* mutant for emRiboSeq libraries; *pol3LG, pol3-L612G* mutant for RHII-HydEn-seq libraries.

Supplementary Figure 7. Different dinucleotide NR preferences are revealed on the leading and lagging strands for wild-type and mutant Pols around 0-200 nt of early-firing ARSs in *rnh201*-null libraries. Heatmap analyses with the normalized frequency of dinucleotides composed of the embedded rNMP (R: rA, rC, rG, or rU) and its upstream neighbor (N: dA, dC, dG or dT) (NR) around early-firing ARSs in *rnh201*-null libraries. The counts for each type of dinucleotide are normalized to the dinucleotide frequencies of the 0-200 nt window for the leading or lagging strand around early-firing ARSs in the sacCer2 reference genome for all the ribose-seq and emRiboSeq libraries, and in the L03 reference genome for all the RHII-HydEn-seq libraries. The normalized frequency means the probability of an rNMP to be embedded in the second position in the dinucleotide. The sum of four normalized frequencies with the same type of embedded rNMP is further normalized to 1. Hence, 0.25 is the expected normalized frequency if there is no rNTP incorporation preference. The corresponding formula used is shown in the Methods section. The background nucleotide frequencies of ribose-seq and emRiboSeq (according to the sacCer2 reference genome), and RHII-HydEn-seq (according to the L03 reference genome) libraries are reported in **Supplementary Table 2A** and **2B**, respectively. The rNMP-embedding position in the dinucleotide is shown in red. Each column of the heatmap shows the results of a specific library. Each row shows results obtained for a type of rNMP. The preferred patterns are indicated with the blue arrows, and they are different on the leading and lagging strand in *rnh201*-null (**A**) wild-type DNA polymerase ribose-seq, emRiboSeq, and RHII-HydEn-seq libraries, (**B**) *pol2* mutant emRiboSeq and RHII-HydEn-seq libraries, and (**C**) *pol3* mutant emRiboSeq and RHII-HydEn-seq libraries. The color bar on the top right shows how normalized frequencies are represented as different colors: black for 0.25; black to yellow for 0.25 to 0.5 – 1, and black to light blue for 0.25 to 0. EM, emRiboSeq libraries; HY, RHII-HydEn-seq libraries; *pol2*: *pol2-M644G* mutant libraries, *pol3*: *pol3-L612M* mutant for emRiboSeq libraries, and *pol3-L612G* mutant for RHII-HydEn-seq libraries.

Supplementary Figure 8. Dinucleotide NR preferences on the leading and lagging strands for wild-type and mutant Pols in *rnh201*-null libraries. Heatmap analyses with the normalized frequency of dinucleotides composed of the embedded rNMP (R: rA, rC, rG, or rU) and its upstream neighbor (N: dA, dC, dG or dT) (NR) around (**A, E**) early-firing ARSs, (**B, F**) late-firing ARSs, (**C, G**) high-efficiency ARSs, and (**D, H**) low-efficiency ARSs in *rnh201*-null libraries. The counts for each type of dinucleotide are normalized to the dinucleotide frequencies of the (**A-D**) 4,000-10,000 nt and (**E-H**) 0-200 nt window for the leading or lagging strand around ARSs in the sacCer2 reference genome for all the ribose-seq and emRiboSeq libraries, and in the L03 reference genome for all the RHII-HydEn-seq libraries. Similar analysis with rNMPs embedded in the 0-200 nt region from estimated collision points of all confirmed ARSs is shown in (**I**). The normalized frequency means the probability of an rNMP to be embedded in the second position in the dinucleotide. The sum of four normalized frequencies with the same type of embedded rNMP is further normalized to 1. Hence, 0.25 is the expected normalized frequency if there is no rNTP incorporation preference. The corresponding formula used is shown in the Methods section. The background nucleotide frequencies of ribose-seq and emRiboSeq (according to the sacCer2 reference genome), and RHII-HydEn-seq (according to the L03 reference genome) libraries are reported in **Supplementary Table 2A** and **2B**,

respectively. The rNMP-embedding position in the dinucleotide is shown in red. Each column of the heatmap shows the results of a specific library. Each row shows results obtained for a type of rNMP. The color bar on the top right shows how normalized frequencies are represented as different colors: black for 0.25; black to yellow for 0.25 to 0.5 – 1, and black to light blue for 0.25 to 0. EM, emRiboSeq libraries; HY, RHII-HydEn-seq libraries; *pol2*: *pol2-M644G* mutant libraries, *pol3*: *pol3-L612M* mutant for emRiboSeq libraries, and *pol3-L612G* mutant for RHII-HydEn-seq libraries.

Supplementary Figure 9. Comparison of rNTP incorporation dinucleotide preference on the leading and lagging strand in *rnh201*-null libraries. Boxplot of dinucleotide (NR) normalized frequency of each rNMP library around (**A**) early-firing ARSs, (**B, E**) late-firing ARSs, (**C, F**) high-efficiency ARSs, and (**D, G**) low-efficiency ARSs. The error bar represents the 1.5 interquartile range (IQR). And the outliers are marked as diamonds. The normalized frequencies of rNTP incorporation in the (**B-D**) 4,000-10,000-nt window and 0-200-nt window, (**A, E-G**) 0-500-nt window and 0-100-nt window on the leading or lagging strands around ARSs are calculated. Similar analysis with rNMPs embedded in the 0-200 nt region from anticipated collision points of all confirmed ARSs is shown in (**H**). Normalized dinucleotide (NR) frequencies of wild-type DNA polymerase libraries (N = 17), *pol2* mutant libraries, including the *pol2-M644G* mutant of emRiboSeq and RHII-HydEn-seq libraries (N = 8)**,** and *pol3* mutant libraries, including *pol3-L612M* mutant of emRiboSeq and *pol3-L612G* mutant of RHII-HydEn-seq libraries (N = 7) are shown, respectively. Mann-Whitney U tests are performed on dinucleotides (NR) with rA in *pol2* mutant libraries, rC in *pol3* mutant libraries, and rA or rC embedded in wild-type DNA polymerase libraries. ns: P>0.05, *: 0.05>P>0.01, **: 0.01>P>0.001, ***: P<0.001.

Supplementary Figure 10. Dinucleotide RN preferences on the leading and lagging strands for wild-type and mutant Pols in *rnh201*-null libraries. Heatmap analyses with the normalized frequency of dinucleotides composed of the embedded rNMP (R: rA, rC, rG, or rU) and its downstream neighbor (N: dA, dC, dG or dT) (RN) around (**A, E**) early-firing ARSs, (**B, F**) late-firing ARSs, (**C, G**) high-efficiency ARSs, and (**D, H**) low-efficiency ARSs in *rnh201*-null libraries. The counts for each type of dinucleotide are normalized to the dinucleotide frequencies of the (**A-D**) 4,000-10,000 nt and (**E-H**) 0-200 nt window for the leading or lagging strand around ARSs in the sacCer2 reference genome for all the ribose-seq and emRiboSeq libraries, and in the L03 reference genome for all the RHII-HydEn-seq libraries. Similar analysis with rNMPs embedded in the 0-200 nt region from estimated collision points of all confirmed ARSs is shown in (**I**). The normalized frequency means the probability of an rNMP to be embedded in the second position in the dinucleotide. The sum of four normalized frequencies with the same type of embedded rNMP is further normalized to 1. Hence, 0.25 is the expected normalized frequency if there is no rNTP incorporation preference. The corresponding formula used is shown in the Methods section. The background nucleotide frequencies of ribose-seq and emRiboSeq (according to the sacCer2 reference genome), and RHII-HydEn-seq (according to the L03 reference genome) libraries are reported in **Supplementary Table 2A** and **2B**, respectively. The rNMP-embedding position in the dinucleotide is shown in red. Each column of the heatmap shows the results of a specific library. Each row shows results obtained for a type of rNMP. The color bar on the top right shows how normalized frequencies are represented as different colors: black for 0.25; black to yellow for 0.25 to 0.5 – 1, and black to light blue for 0.25 to 0. EM, emRiboSeq libraries; HY, RHII-HydEn-seq libraries; *pol2*: *pol2-M644G* mutant libraries, *pol3*: *pol3-L612M* mutant for emRiboSeq libraries, and *pol3-L612G* mutant for RHII-HydEn-seq libraries.

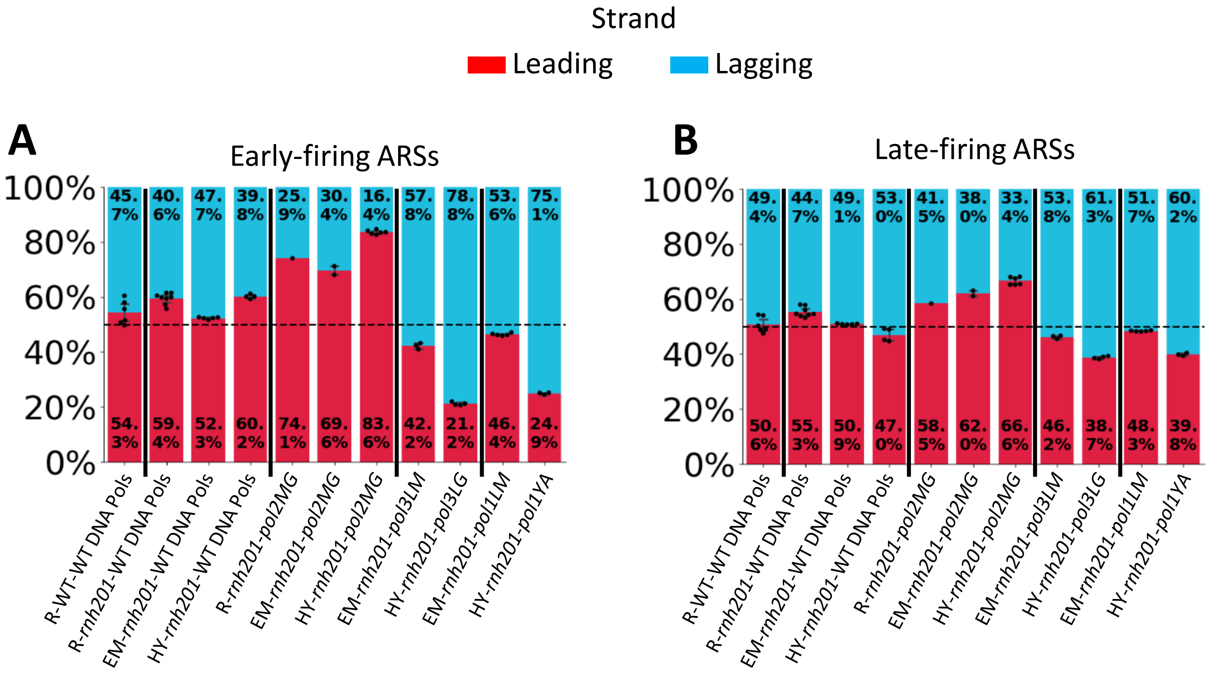

Supplementary Figure 1

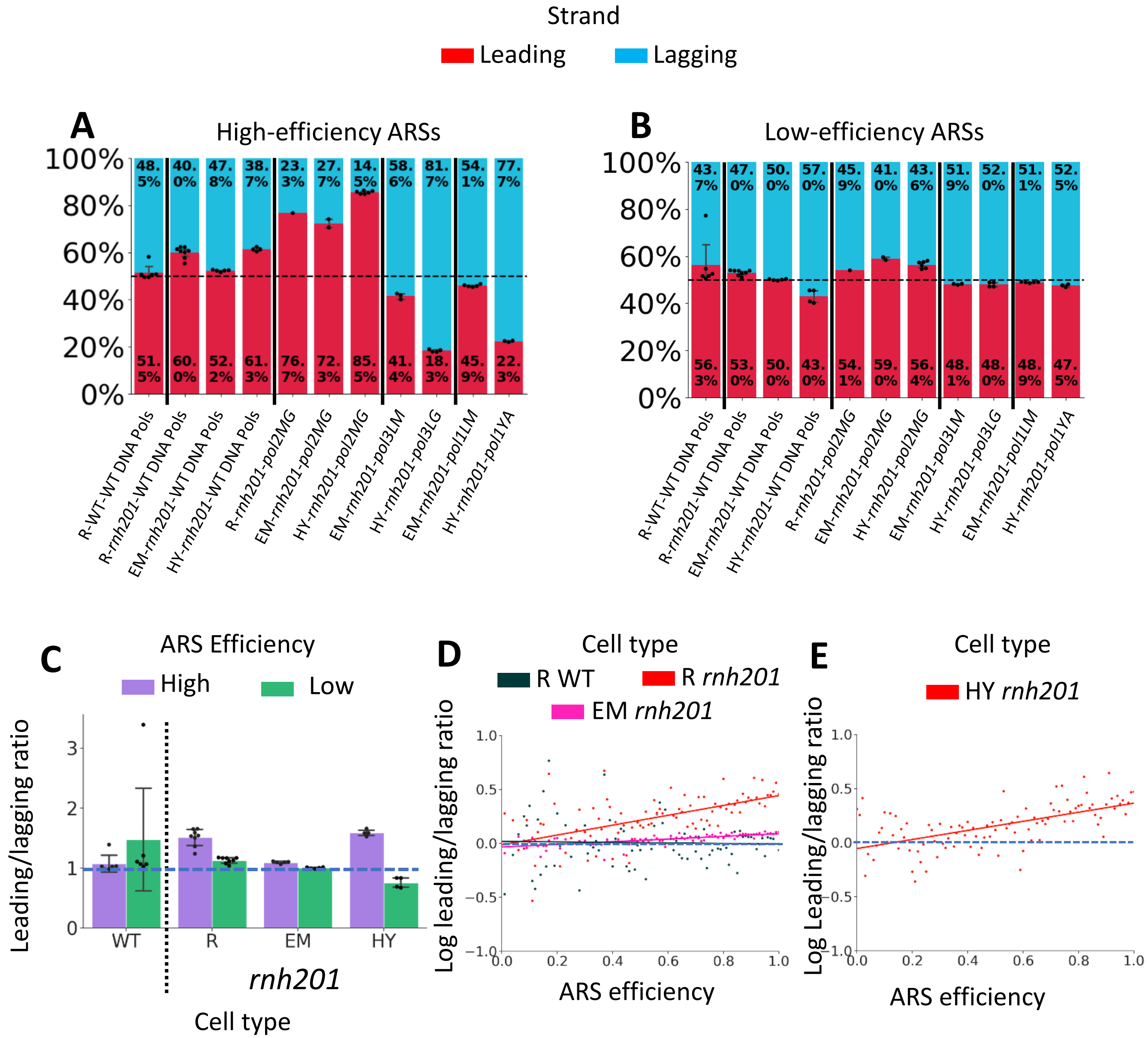

Supplementary Figure 2

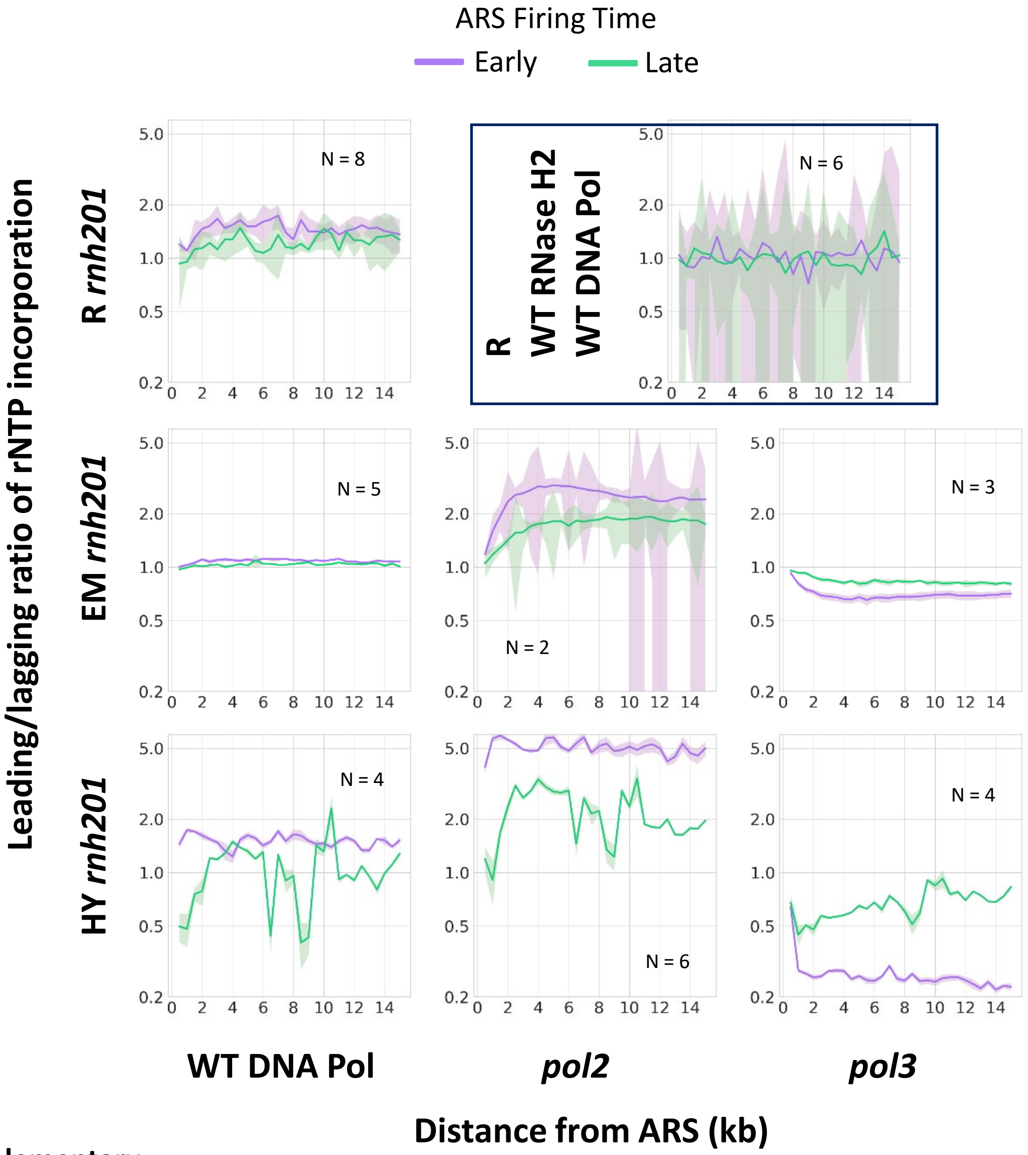

Supplementary Figure 3

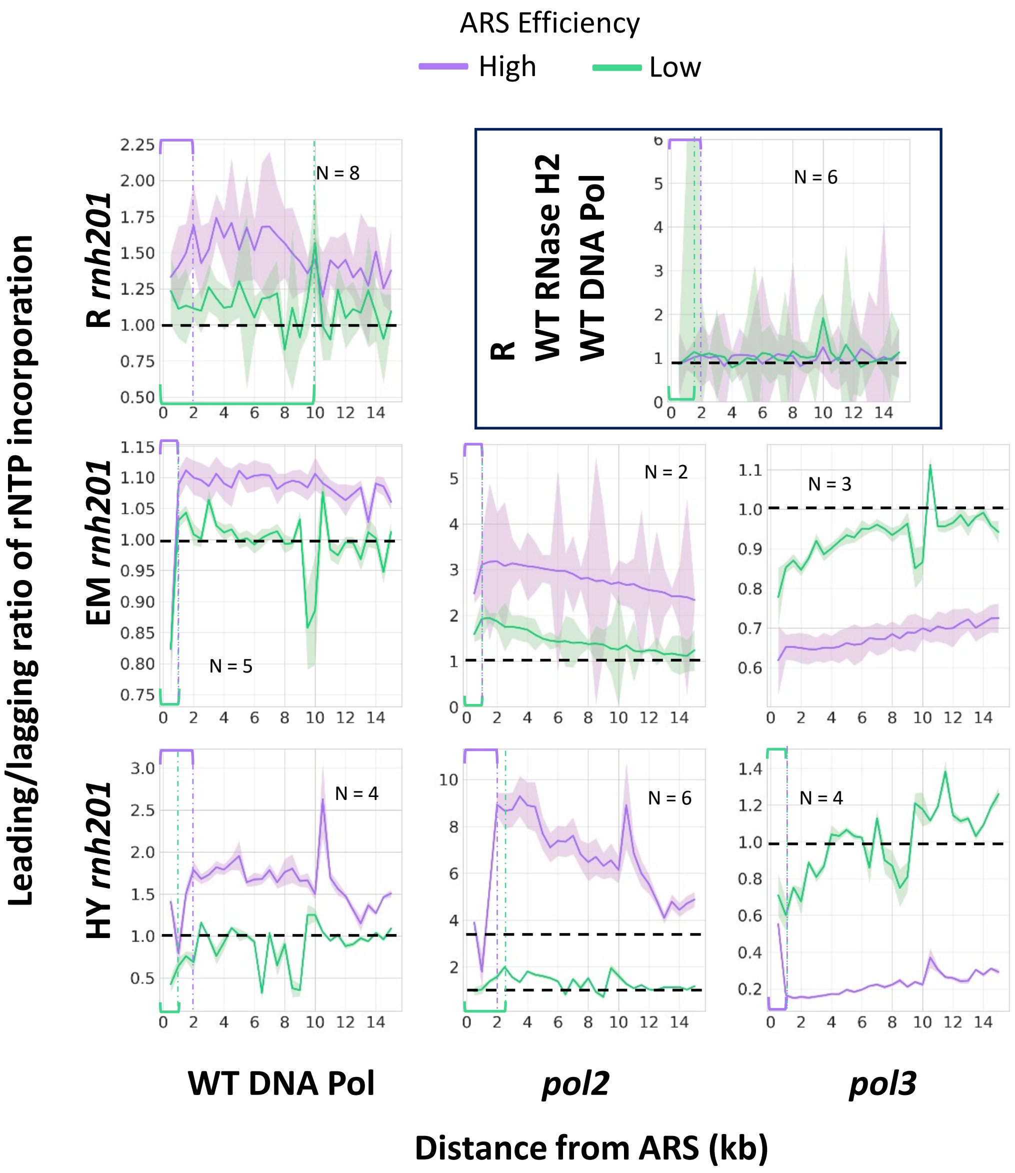

Supplementary Figure 4

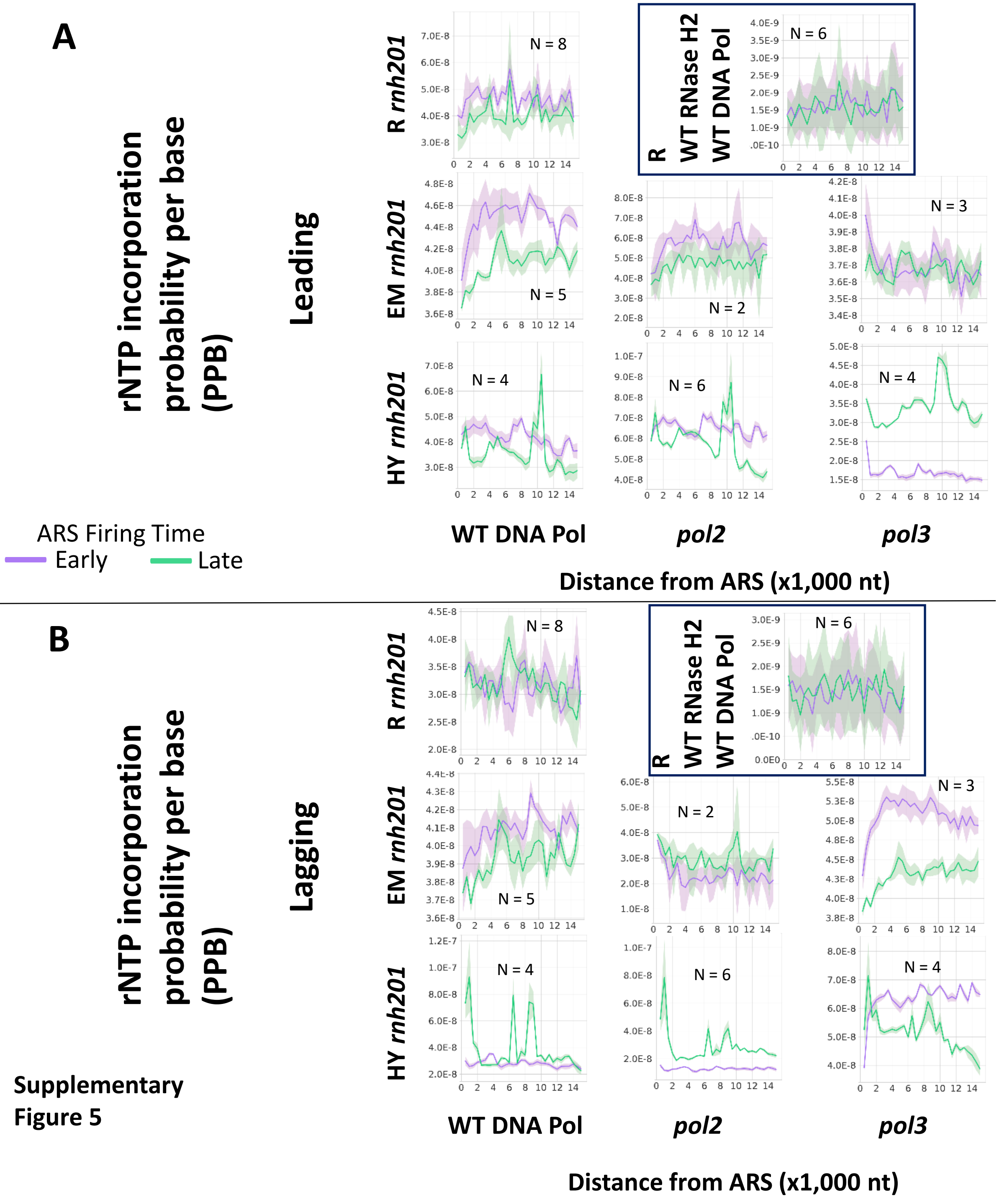

Supplementary Figure 5

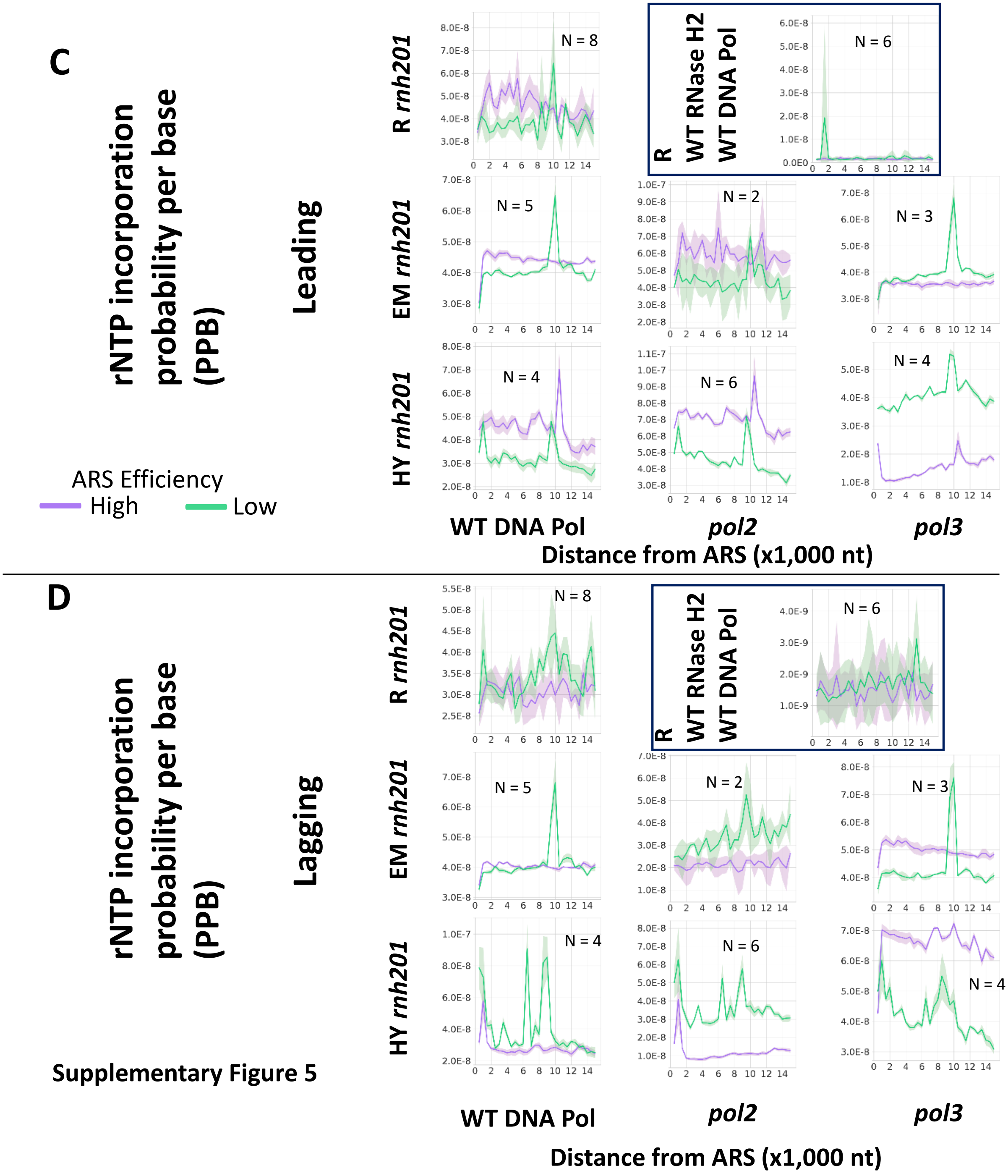

Supplementary Figure 5

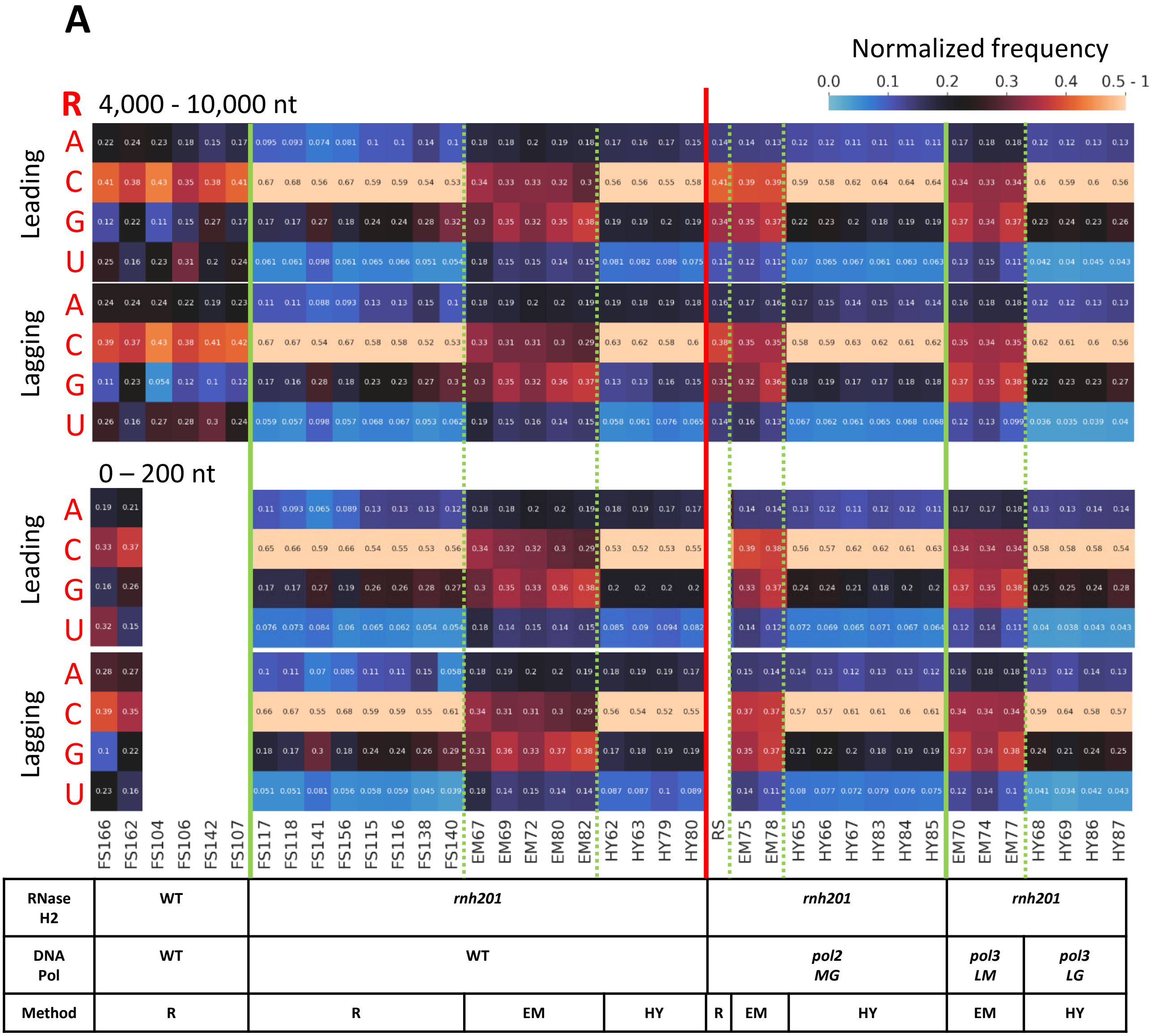

Supplementary Figure 6 Late-firing ARS

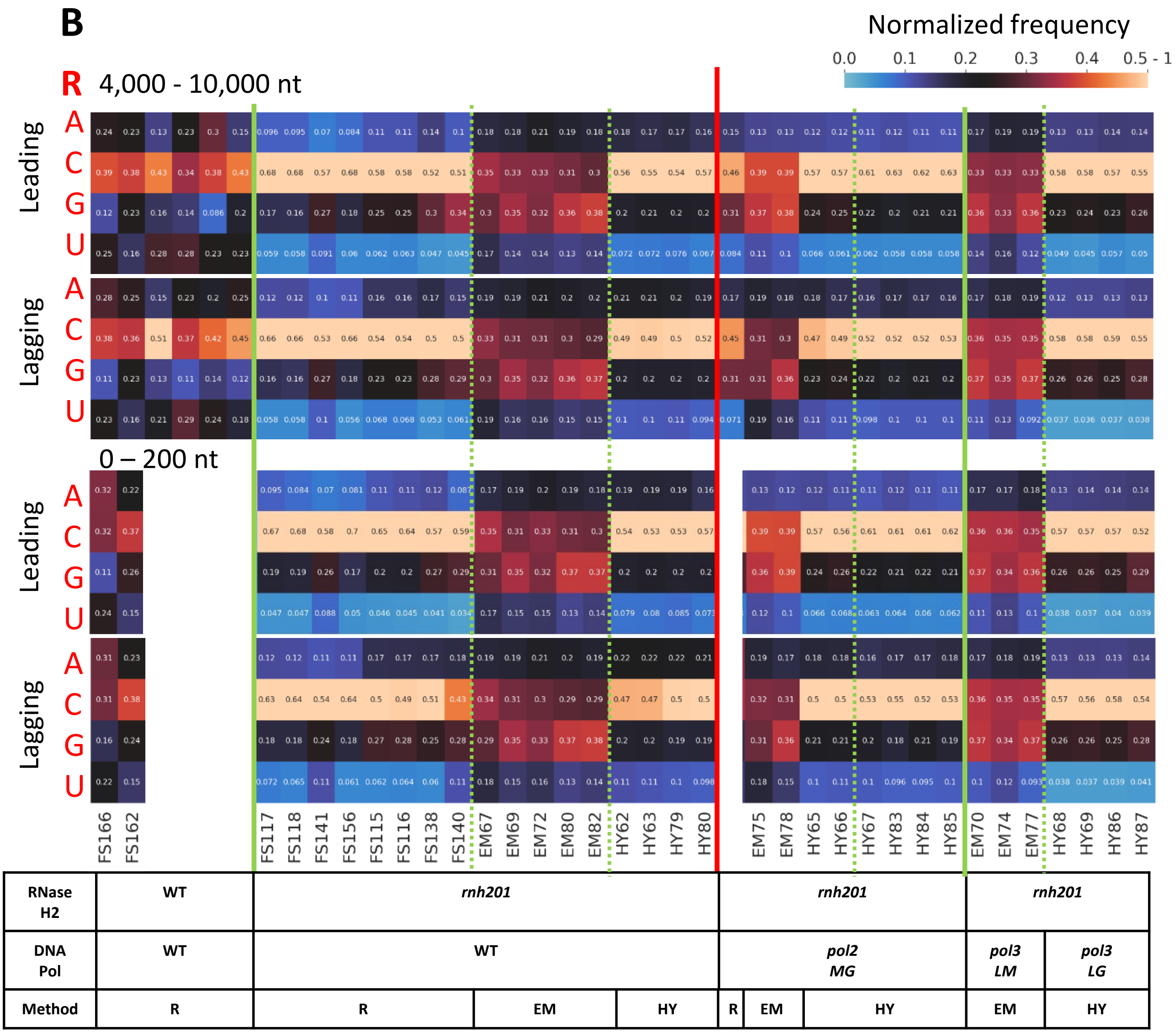

High-efficiency ARS

Supplementary Figure 6

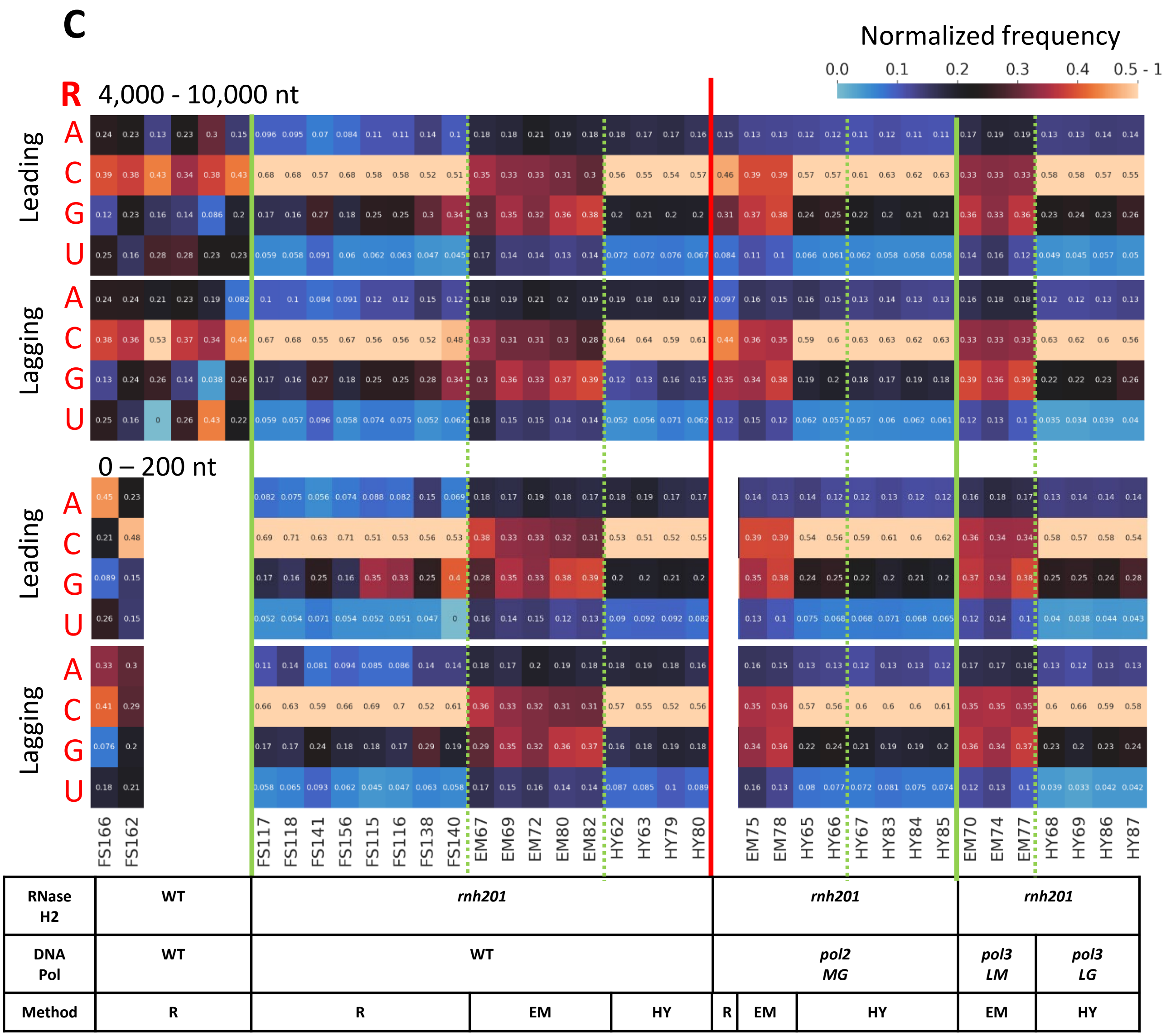

Low-efficiency ARS

Supplementary Figure 6

D

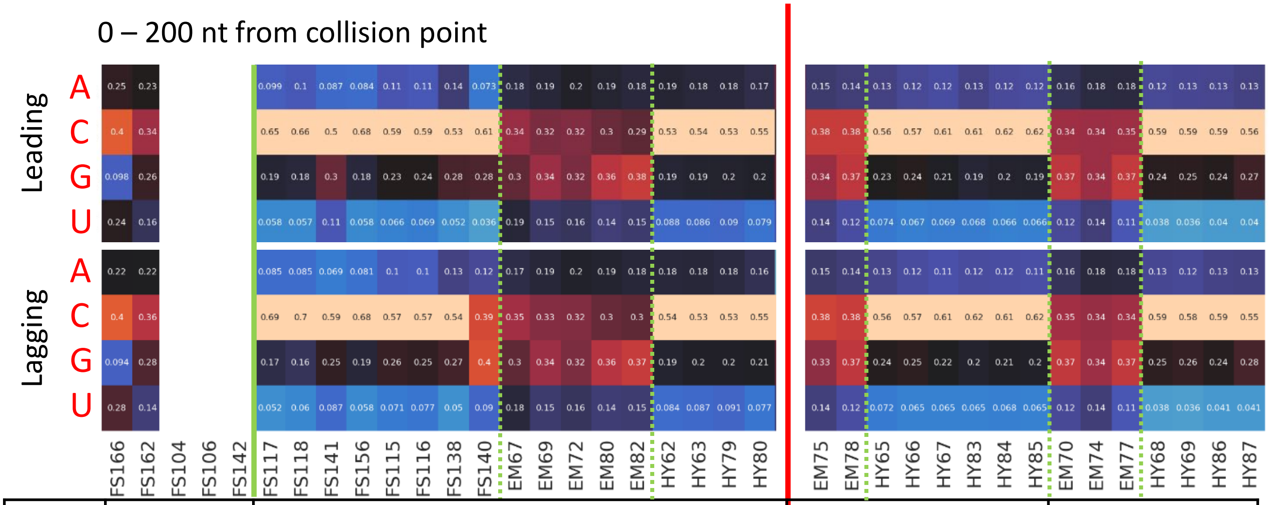

RNase H2	WT	*rnh201*			*rnh201*		*rnh201*	
DNA Pol	WT	WT			*pol2 MG*		*pol3 LM*	*pol3 LG*
Method	R	R	EM	HY	EM	HY	EM	HY

Termination zone

Supplementary Figure 6

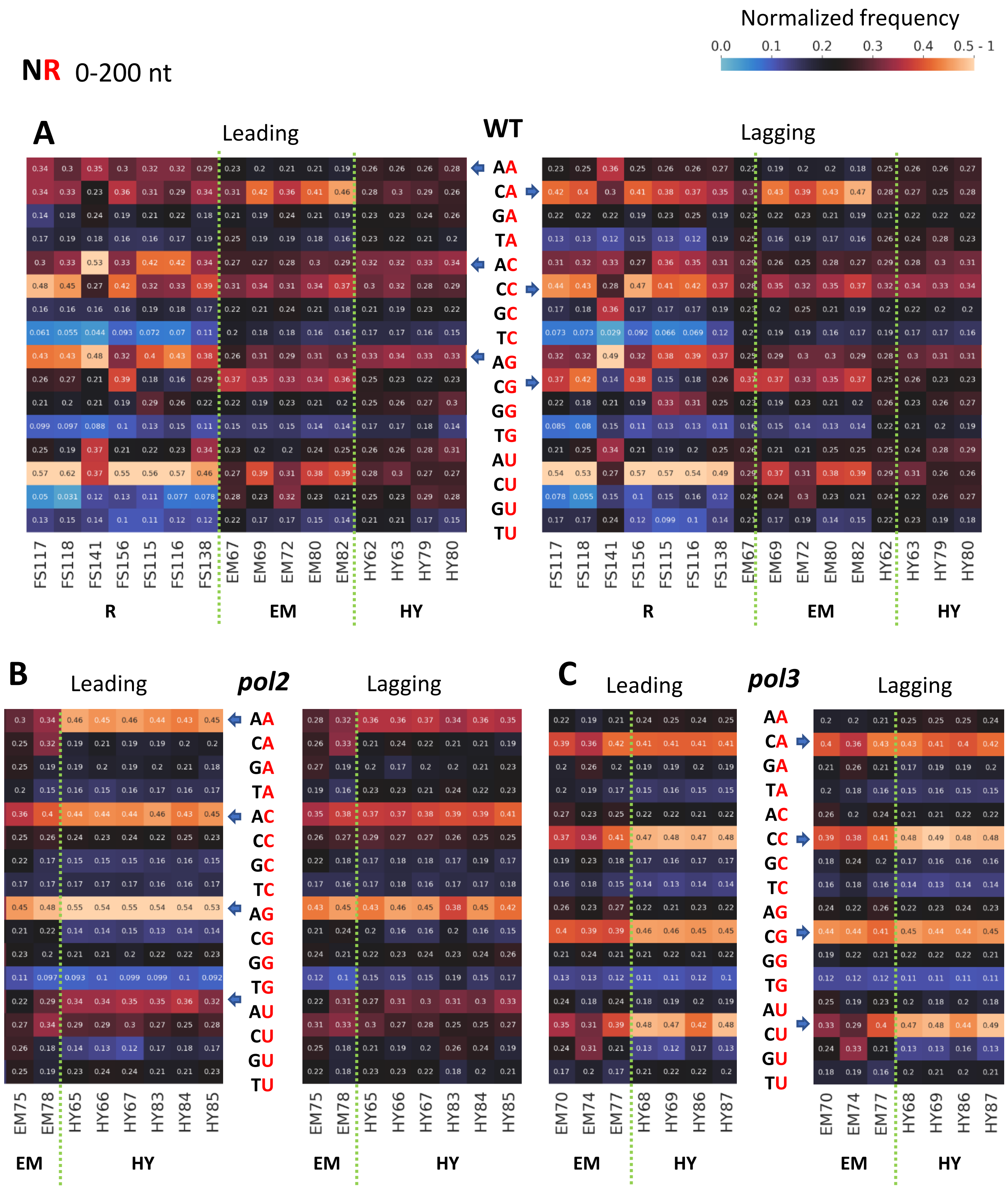

Supplementary Figure 7

Early-firing ARS

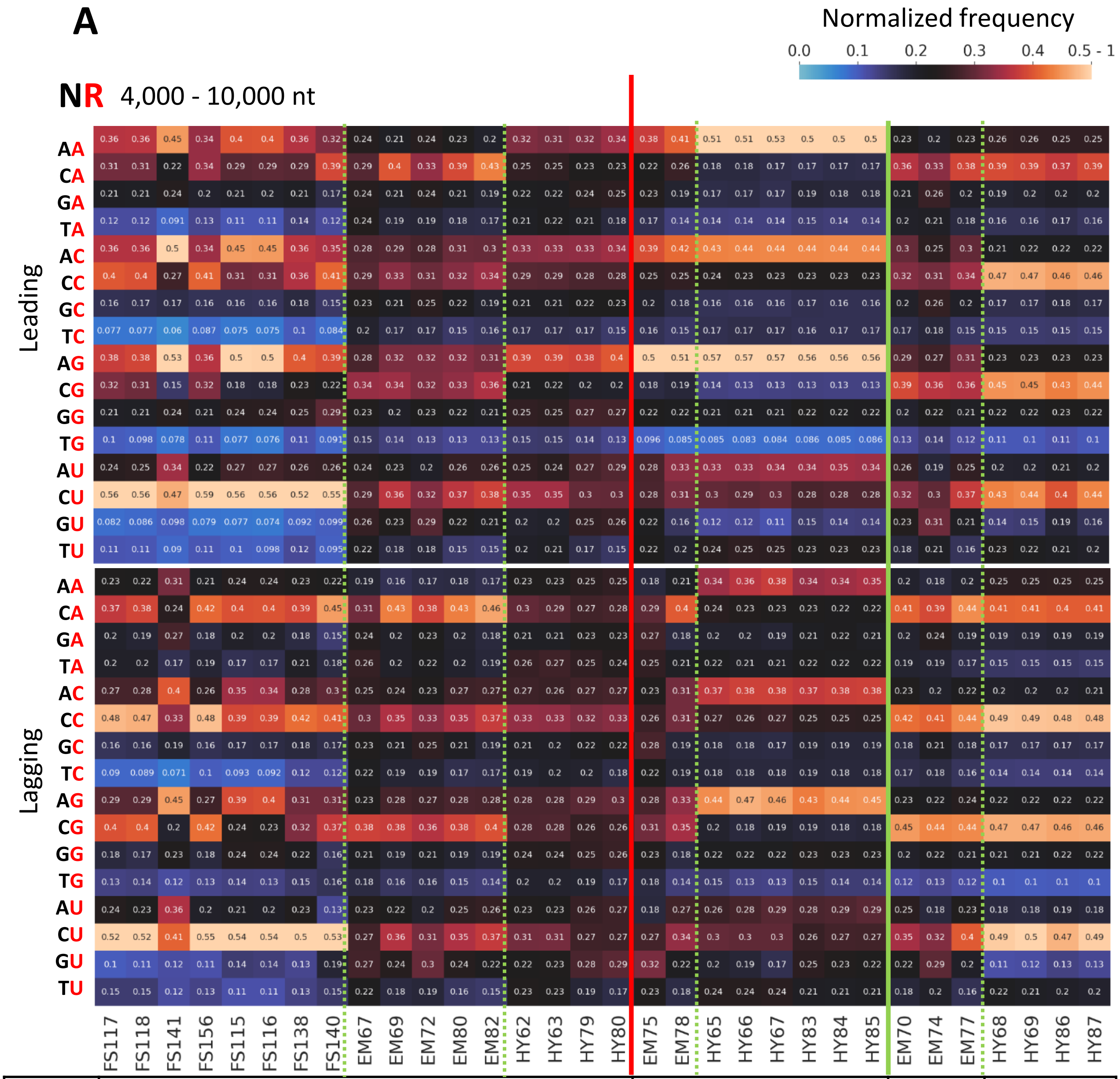

DNA Pol	WT			*pol2MG*		*pol3LM*	*pol3LG*
Method	R	EM	HY	EM	HY	EM	HY

Early-firing ARS

Supplementary Figure 8

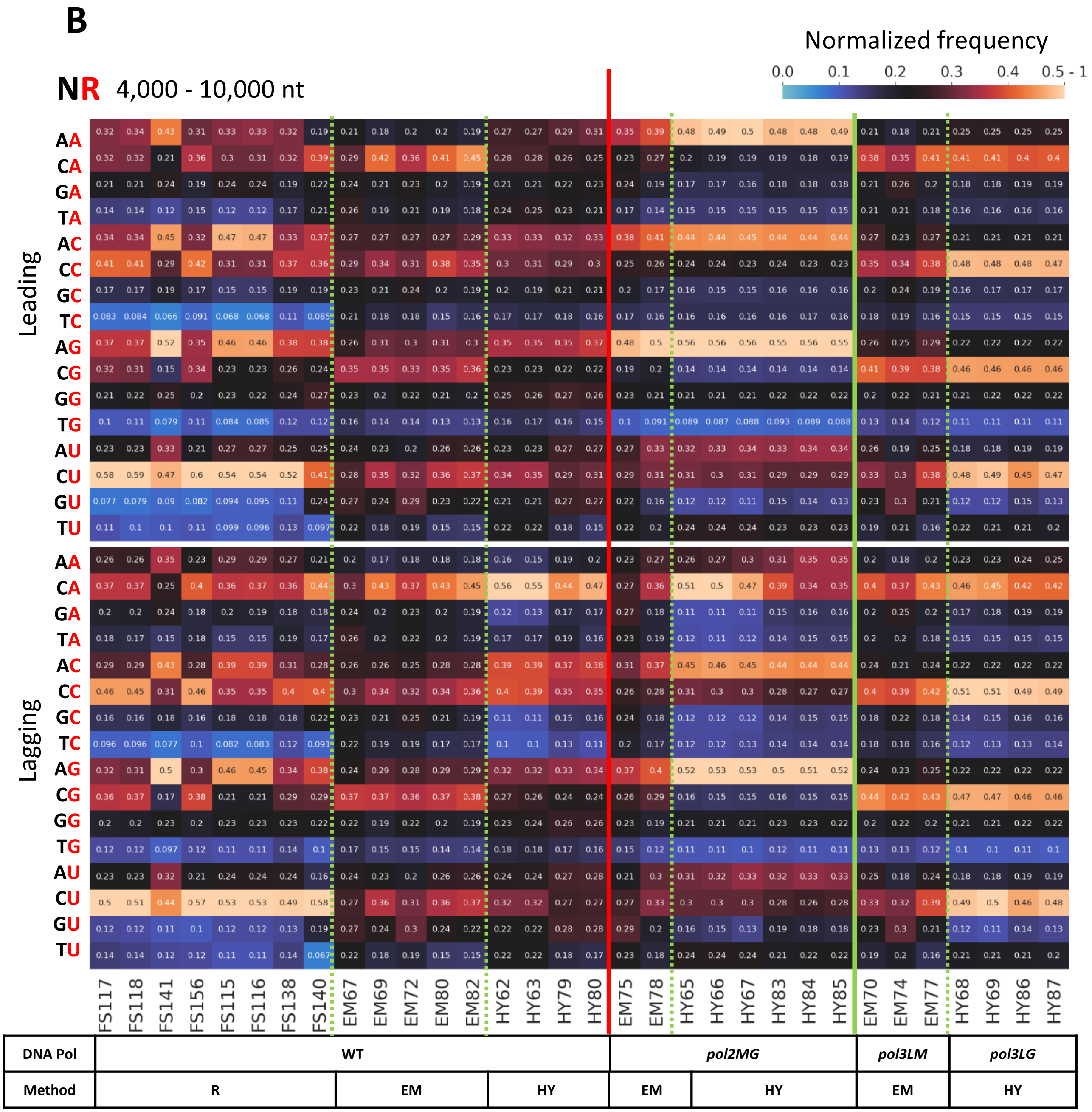

Late-firing ARS

Supplementary Figure 8

C

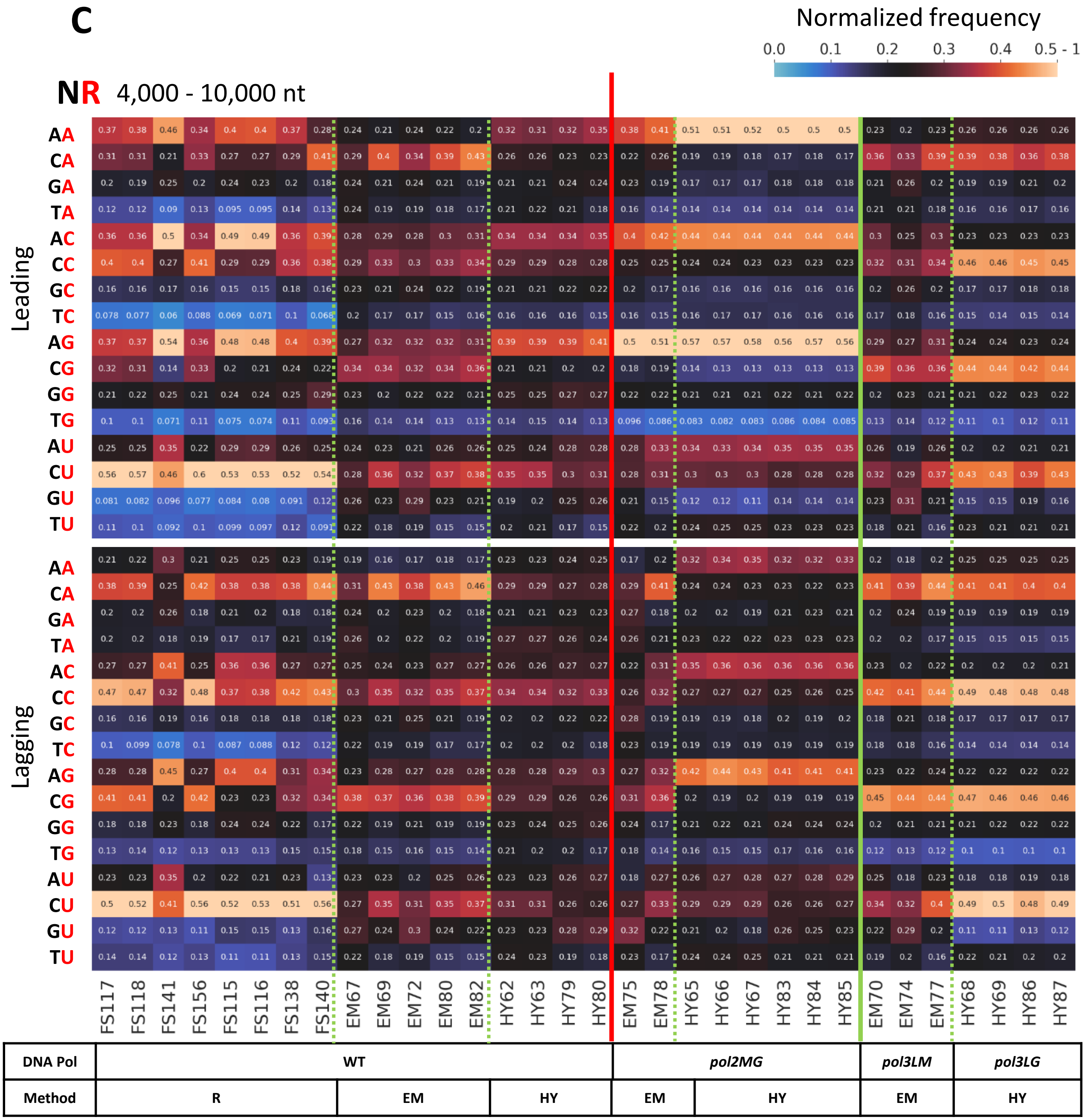

High-efficiency ARS

Supplementary Figure 8

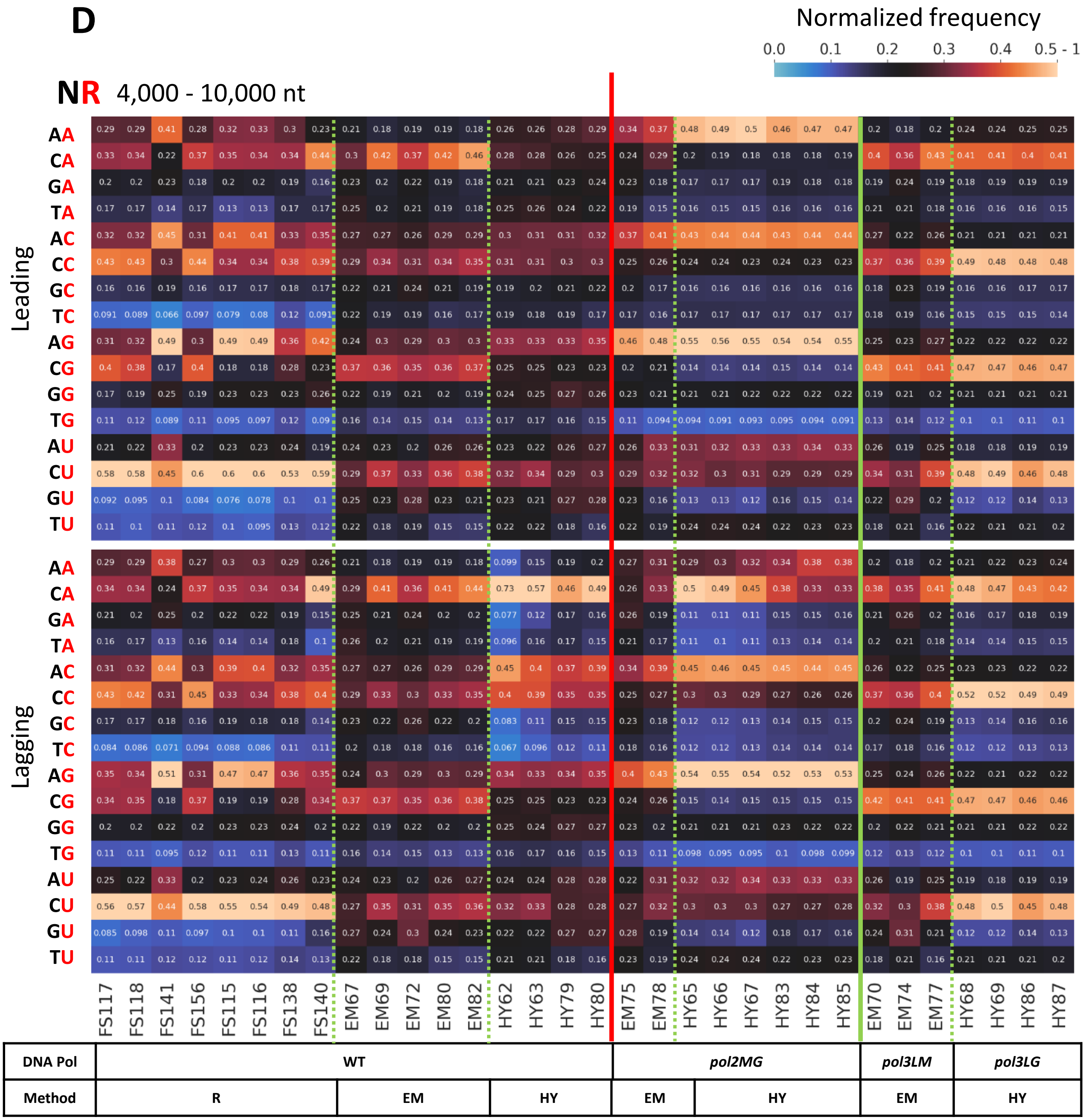

Low-efficiency ARS

Supplementary Figure 8

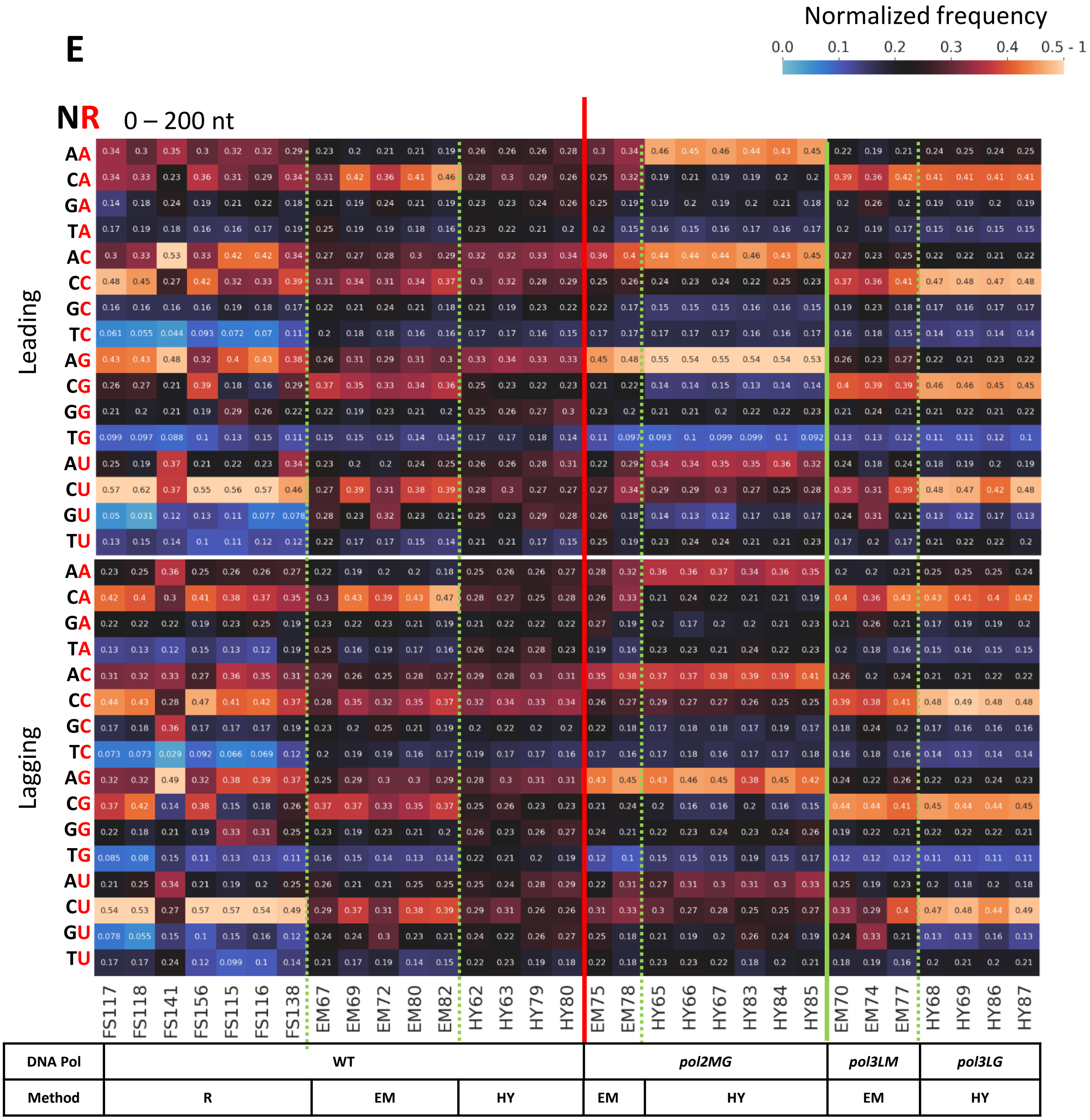

Early-firing ARS

Supplementary Figure 8

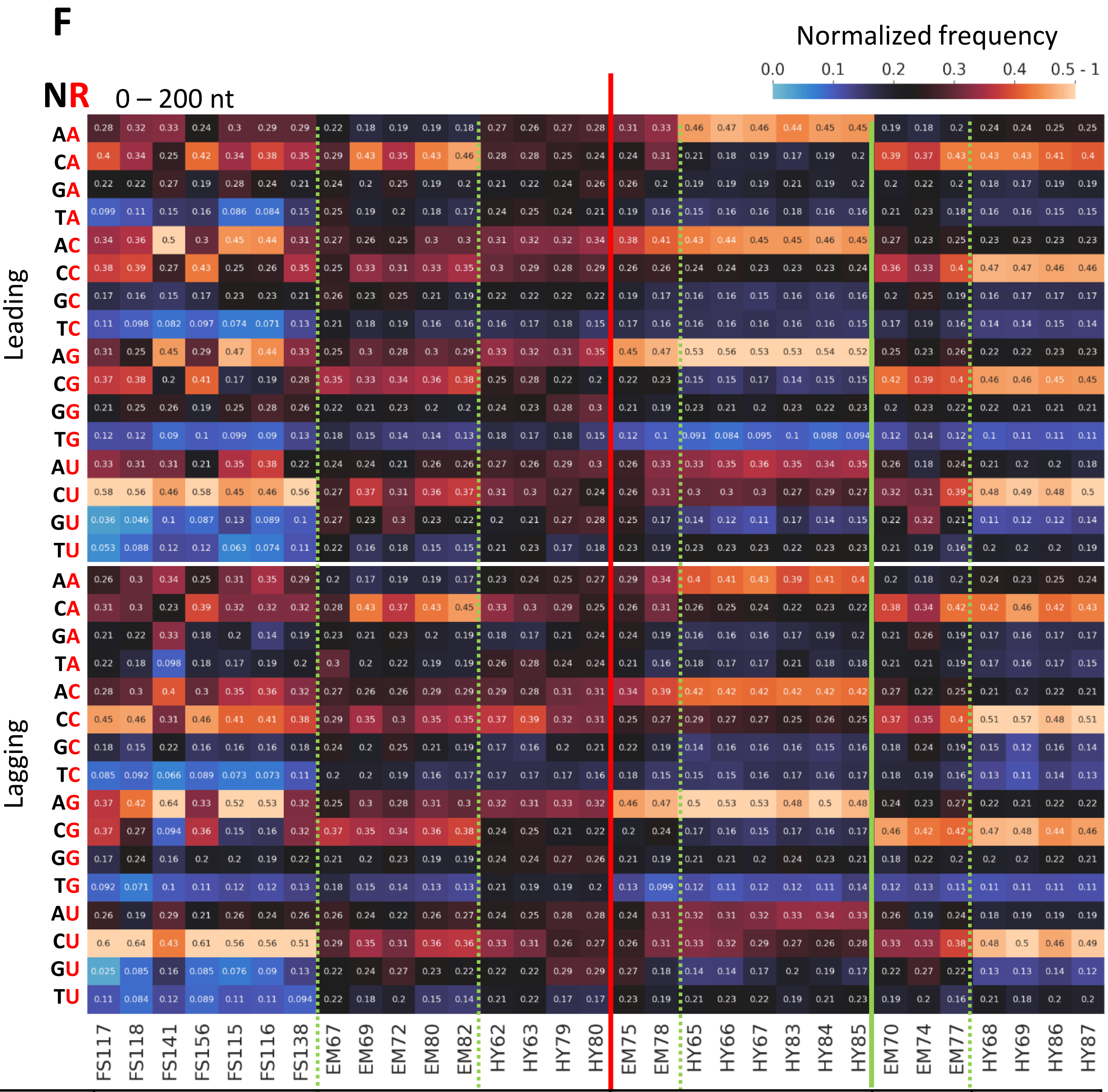

DNA Pol	WT			*pol2MG*		*pol3LM*	*pol3LG*
Method	R	EM	HY	EM	HY	EM	HY

Late-firing ARS

Supplementary Figure 8

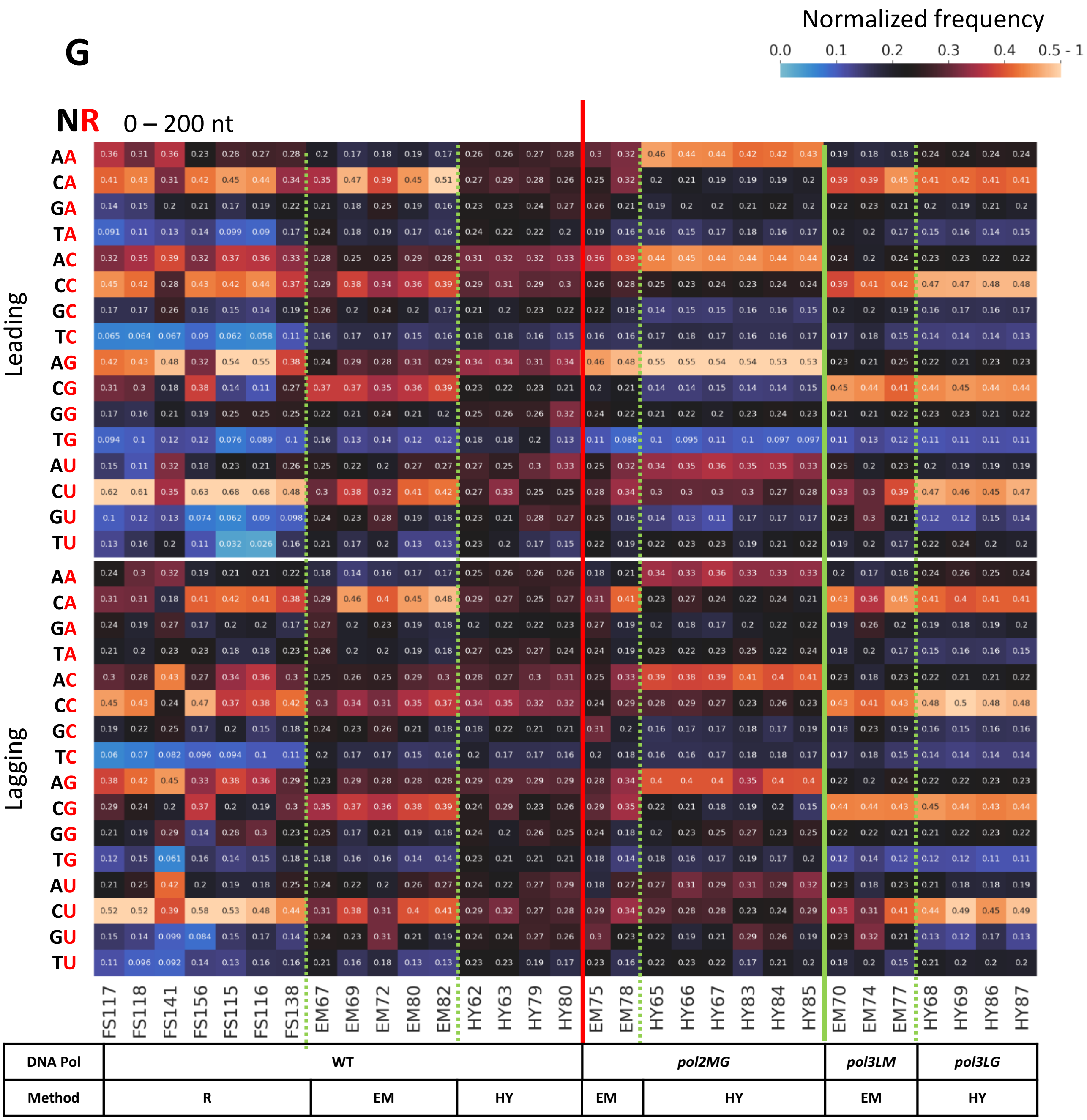

High-efficiency ARS

Supplementary Figure 8

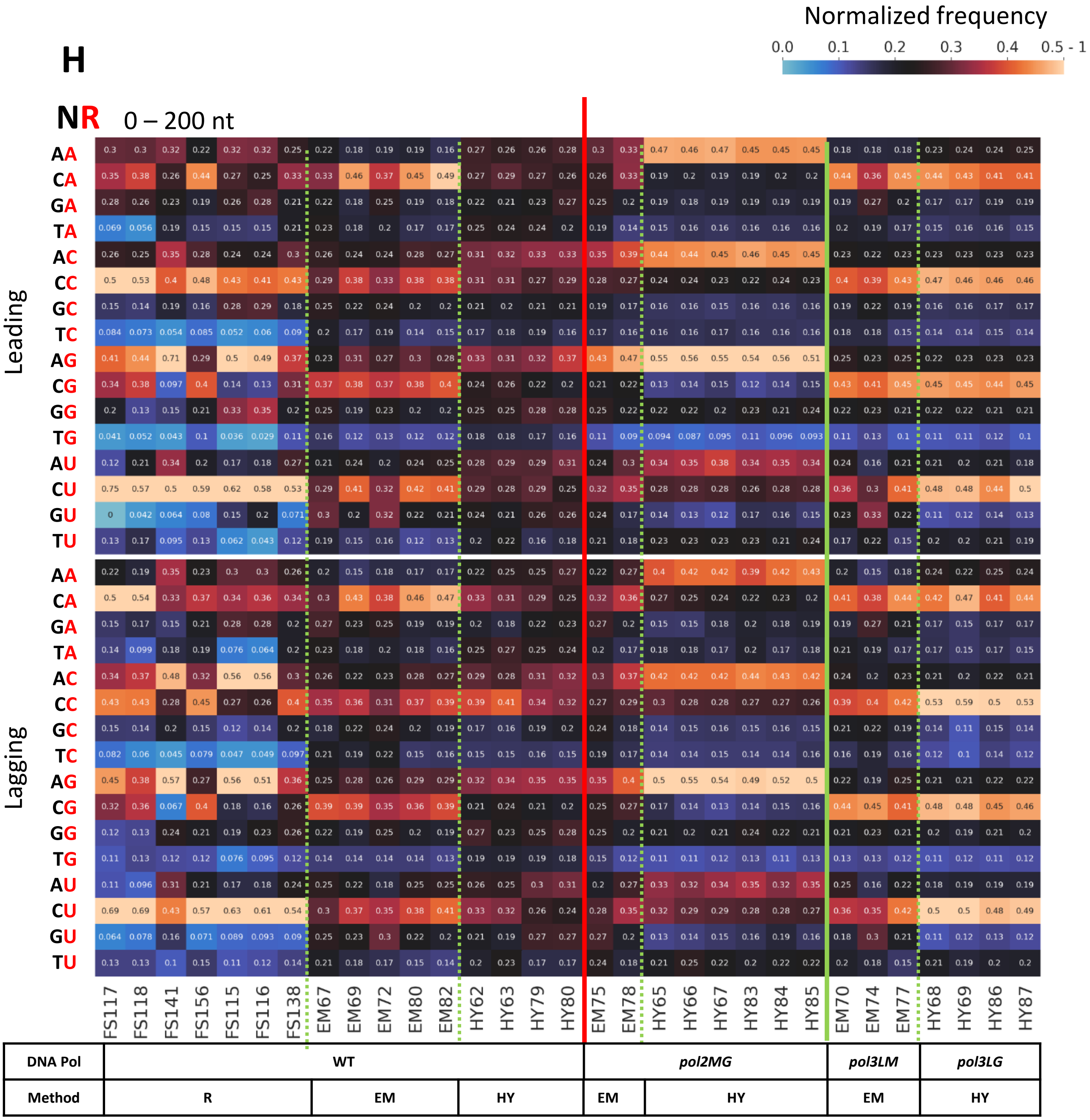

Low-efficiency ARS

Supplementary Figure 8

I

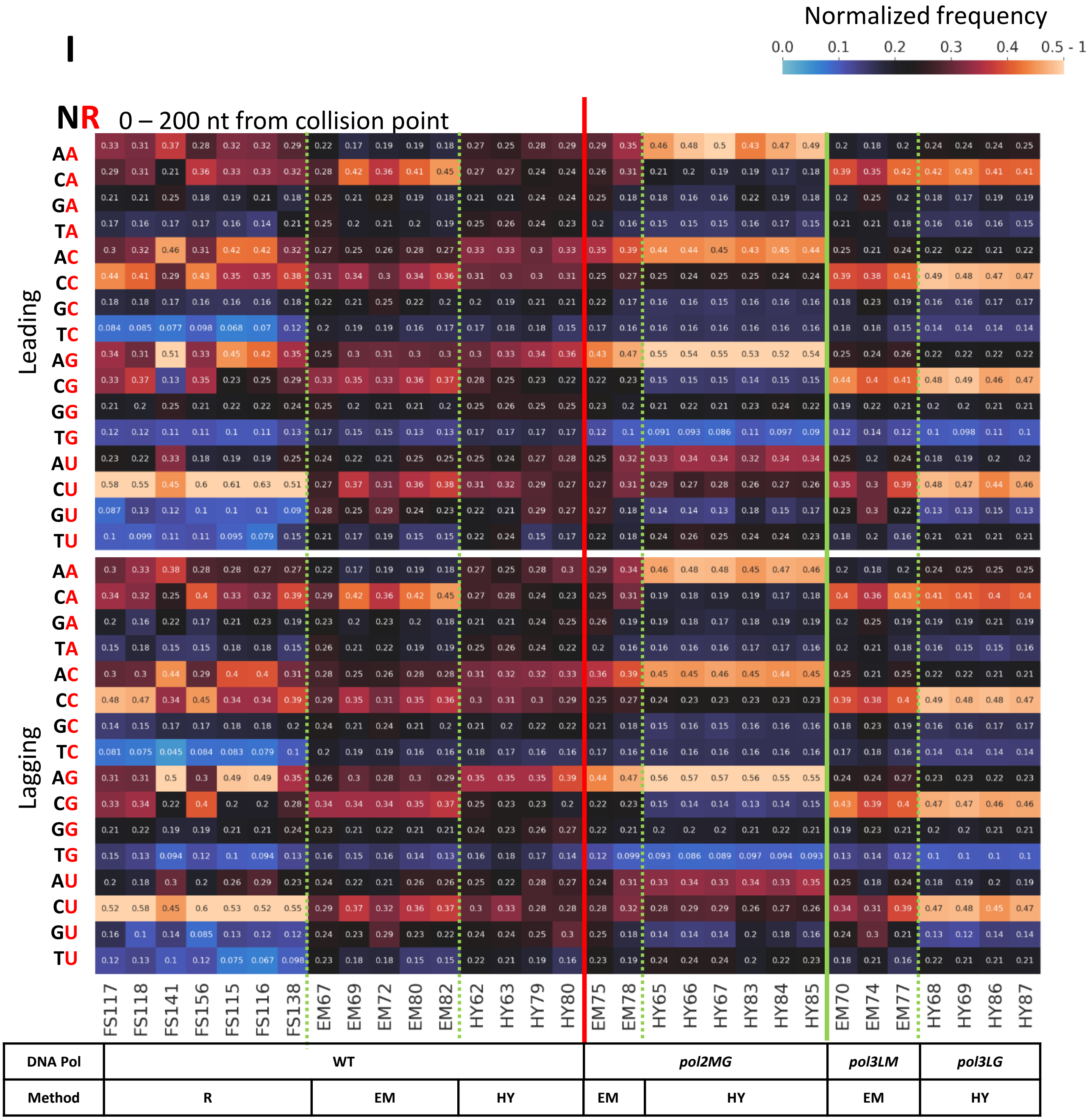

Termination zone

Supplementary Figure 8

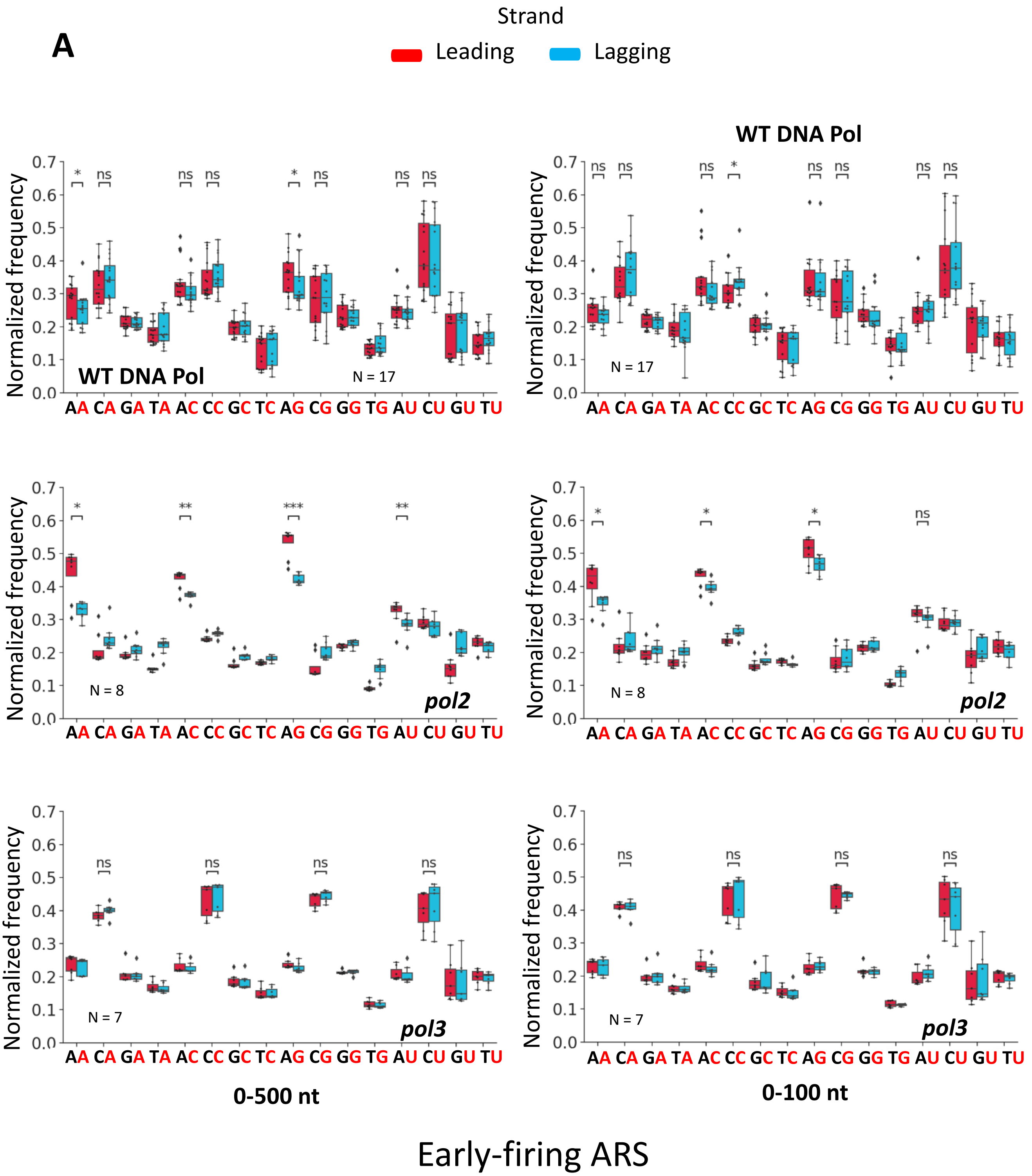

Supplementary Figure 9

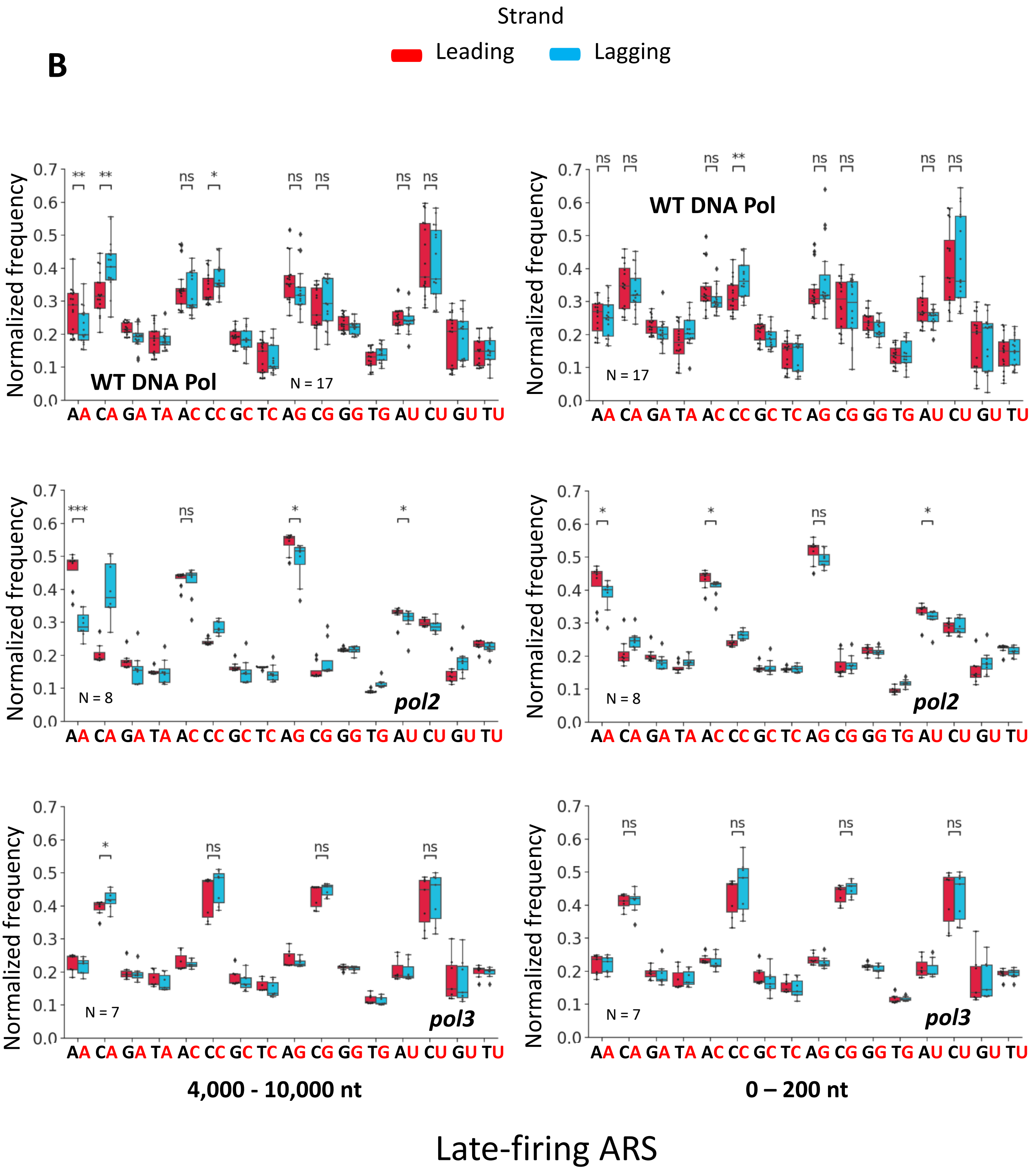

Supplementary Figure 9

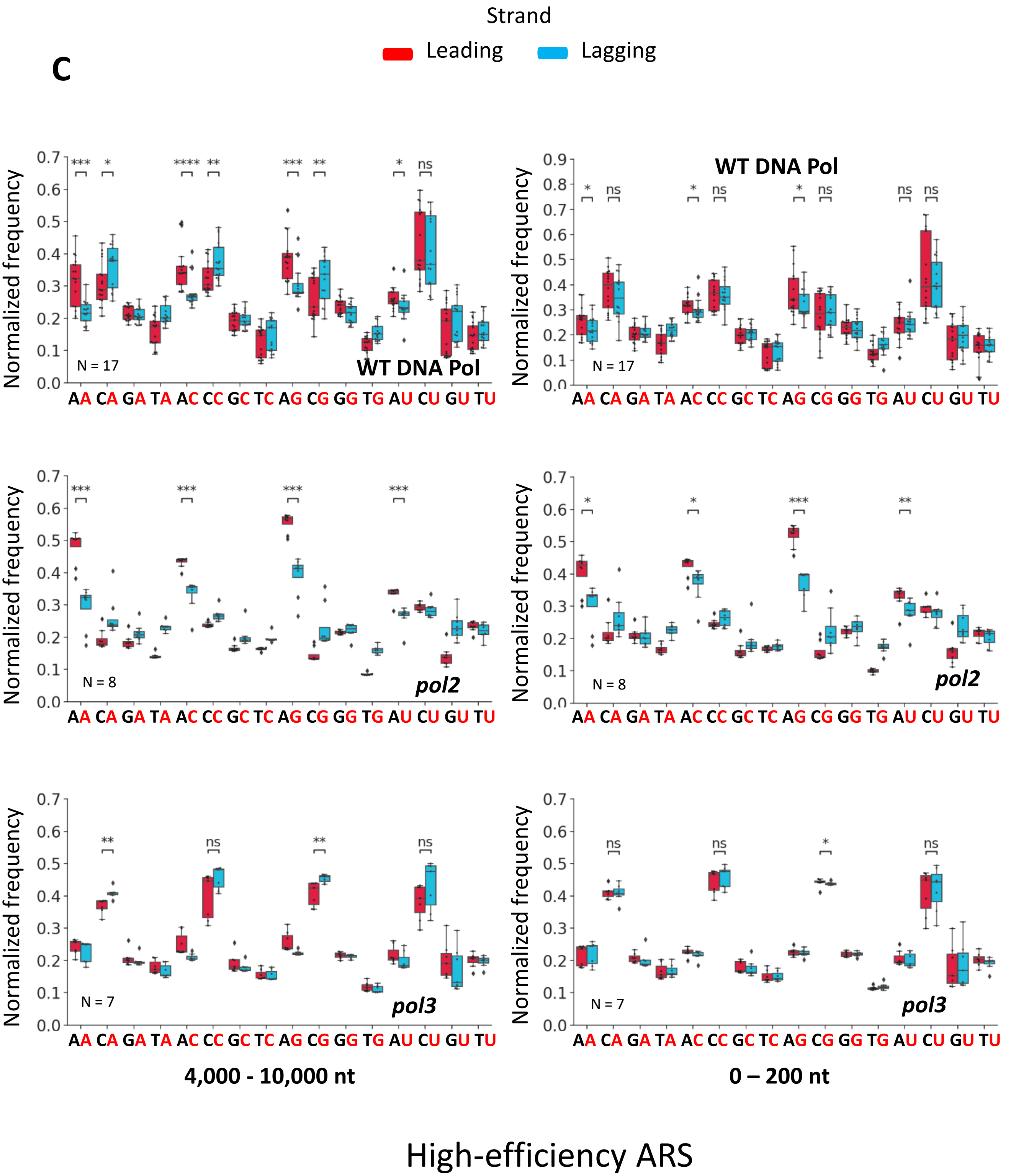

Supplementary Figure 9

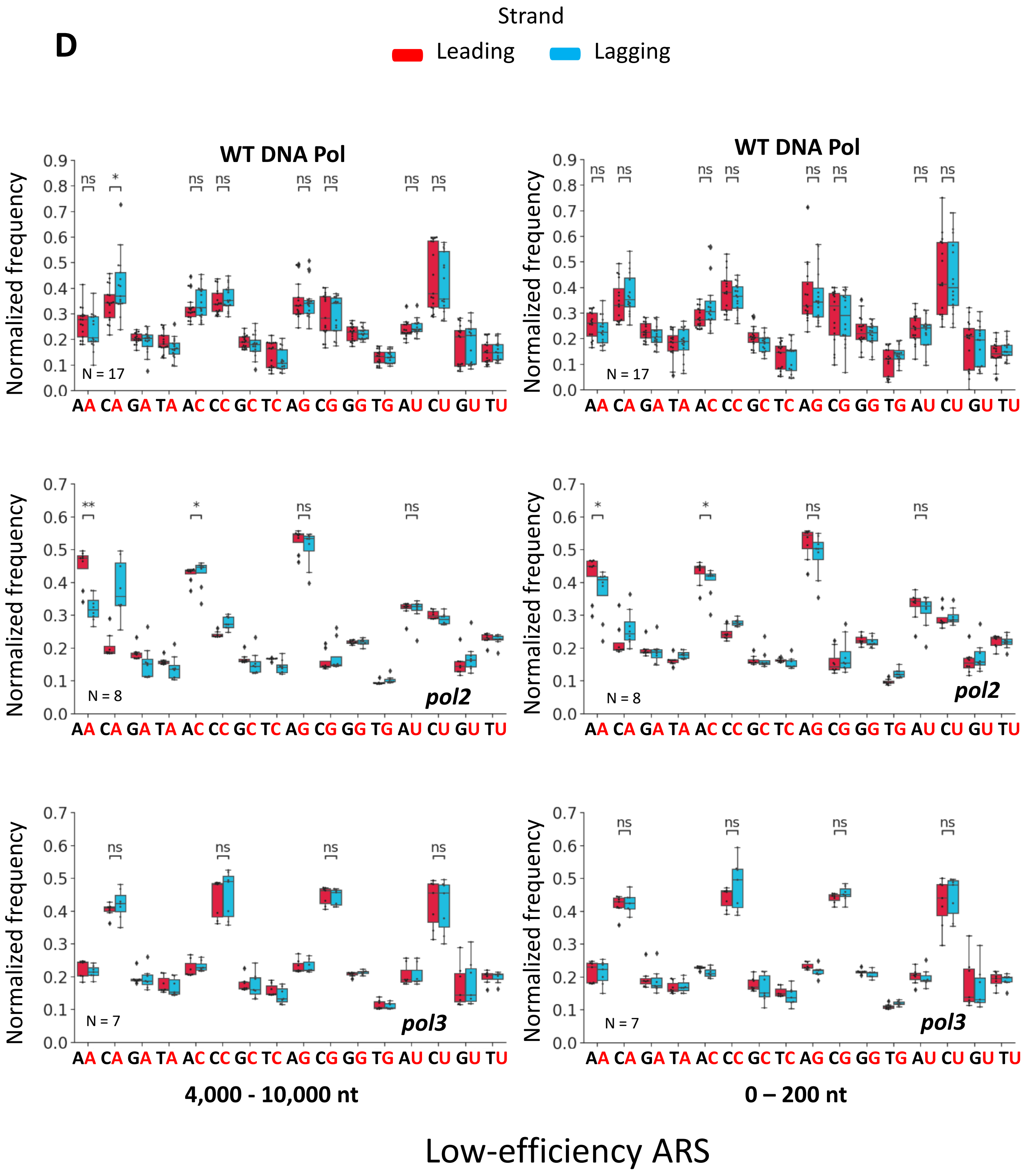

Supplementary Figure 9

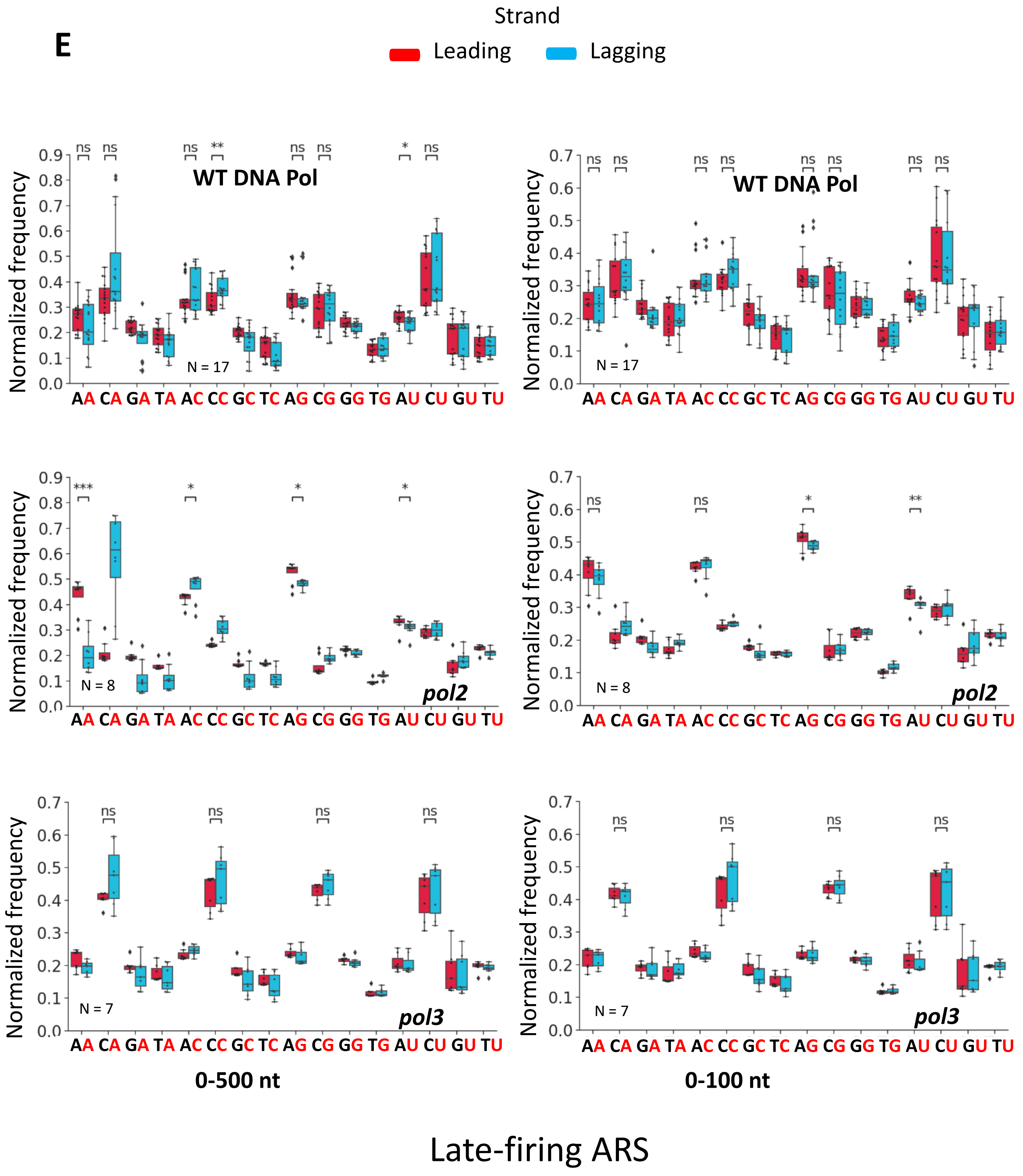

Supplementary Figure 9

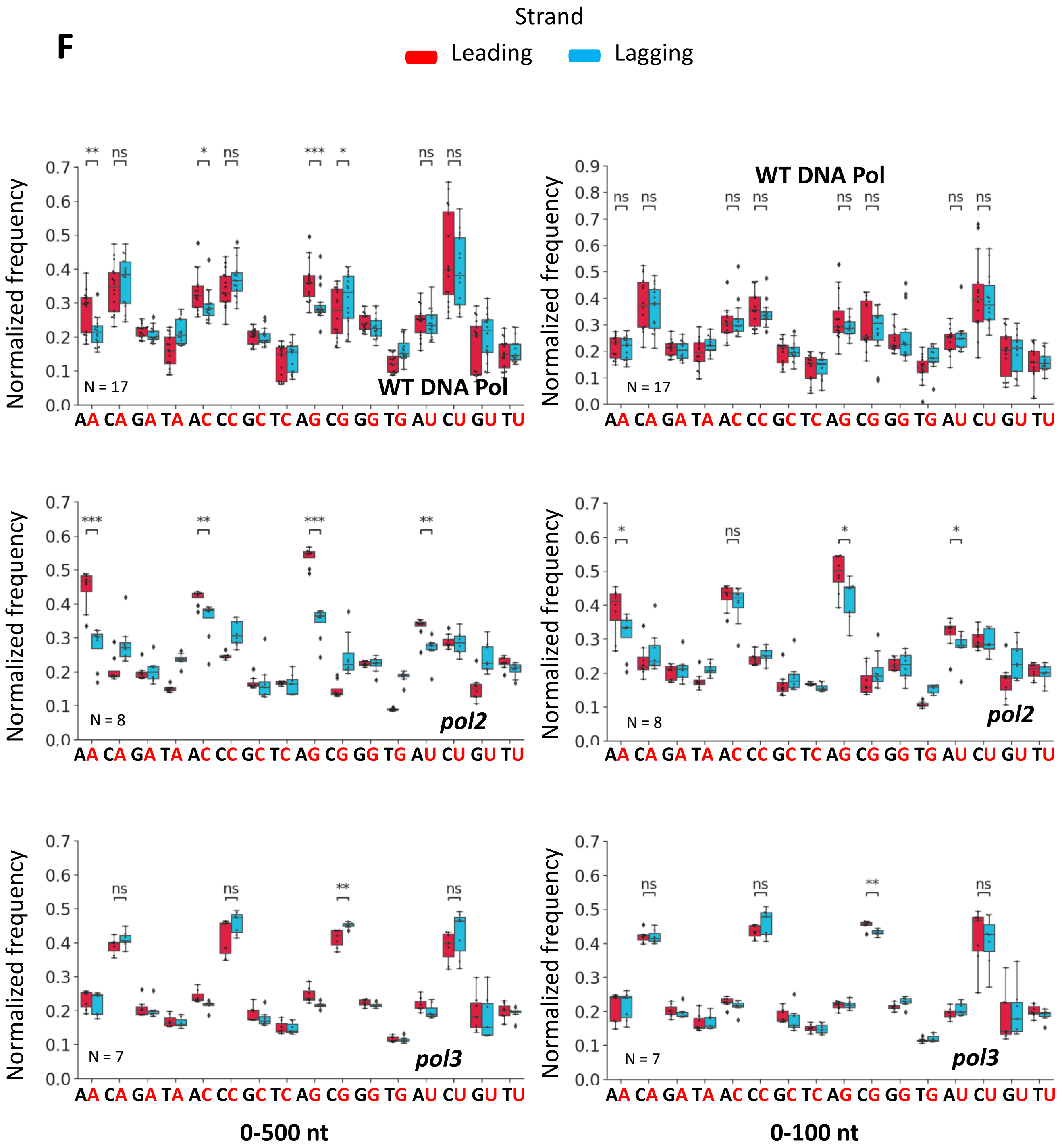

High-efficiency ARS

Supplementary Figure 9

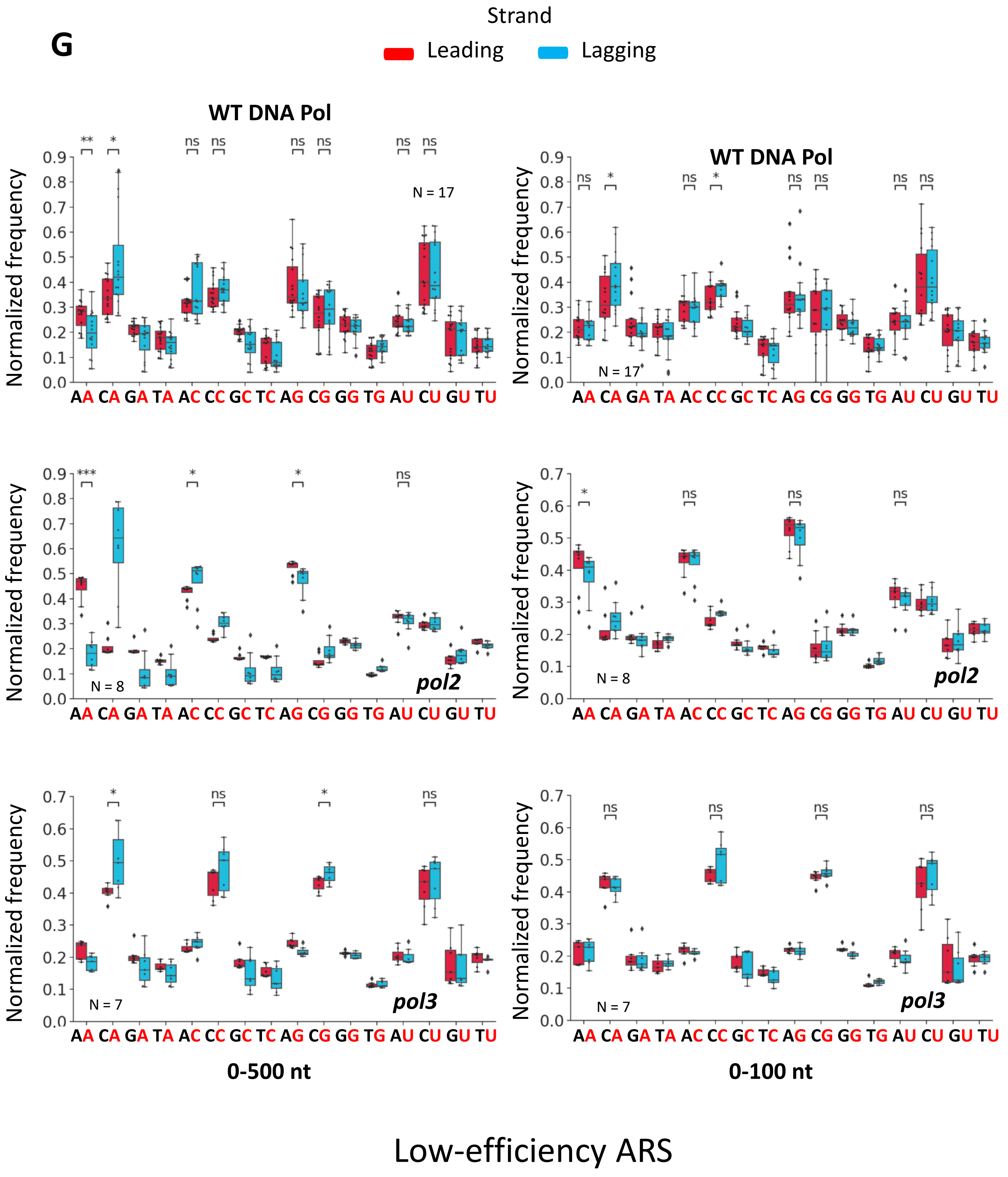

Supplementary Figure 9

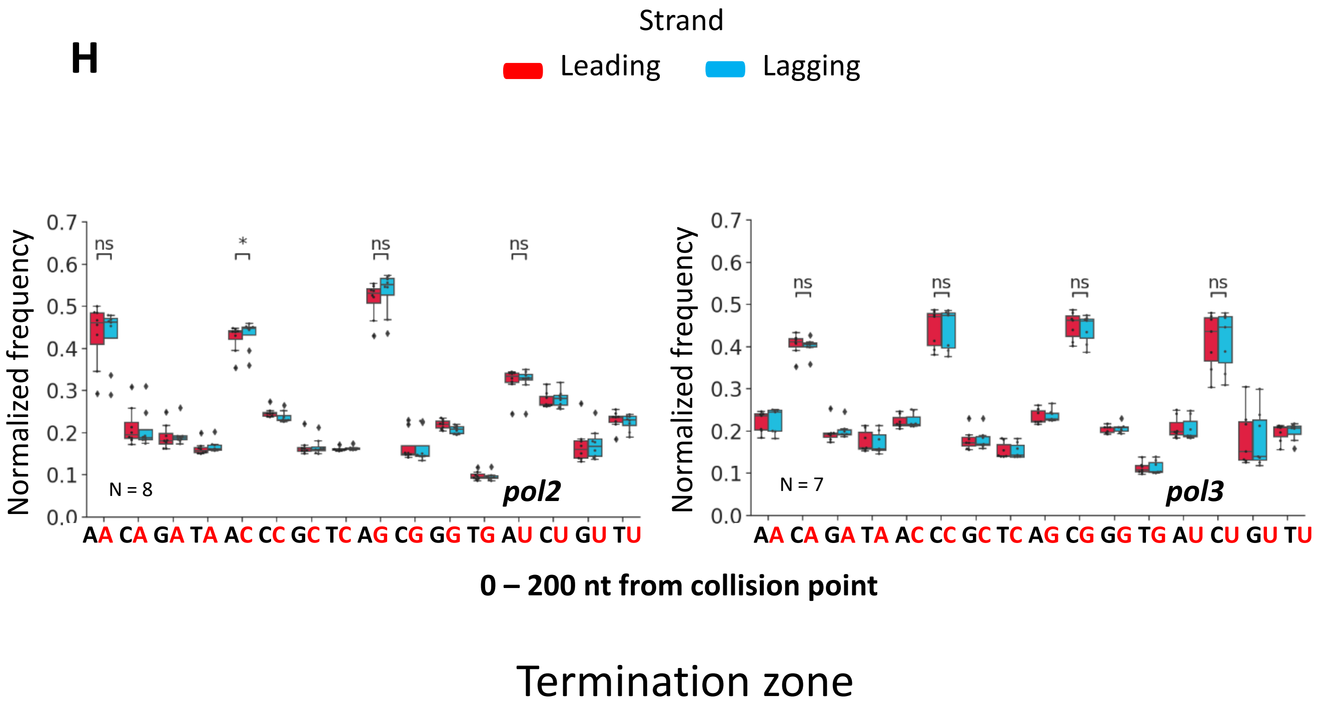

Supplementary Figure 9

A

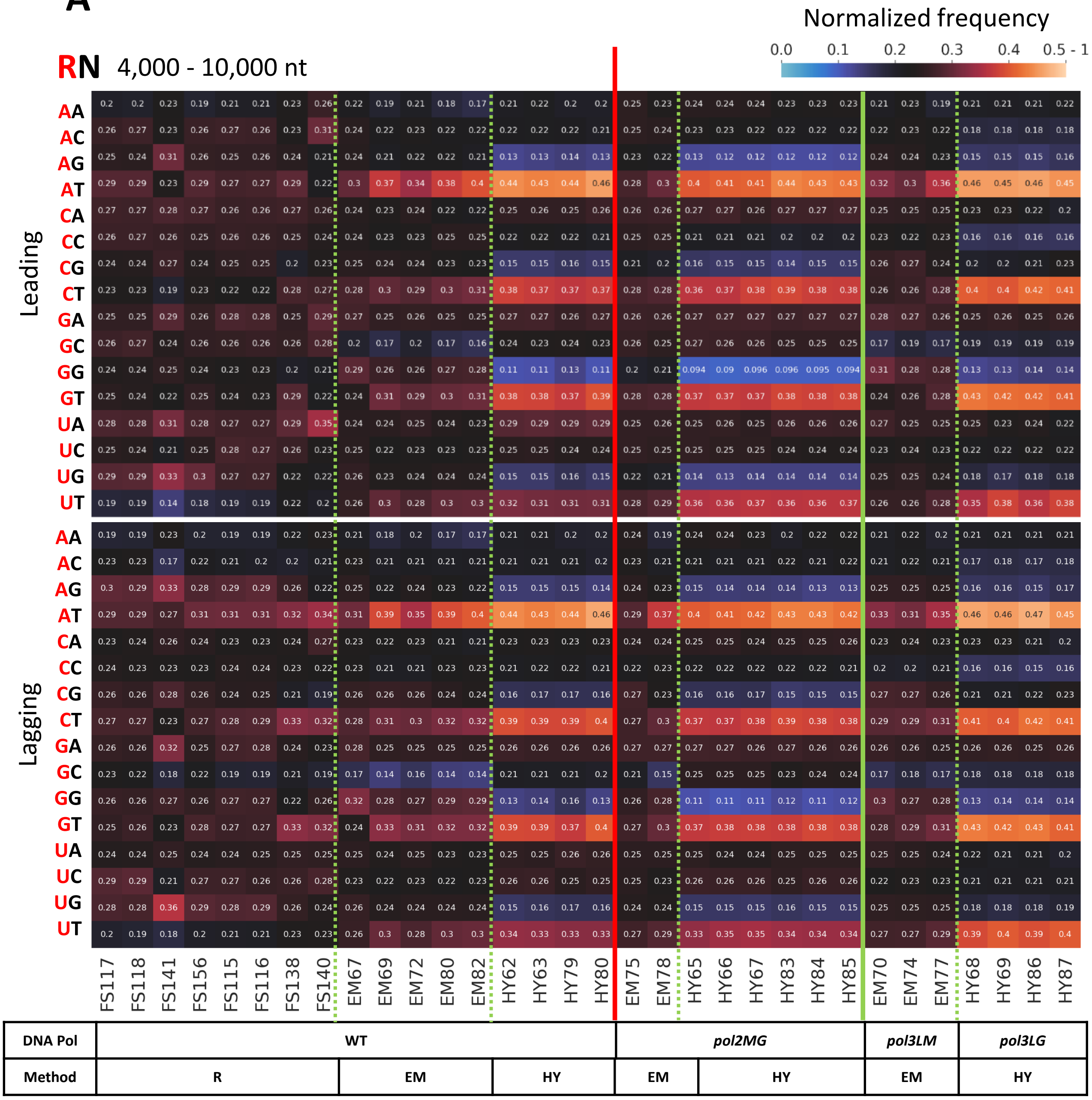

Early-firing ARS

Supplementary Figure 10

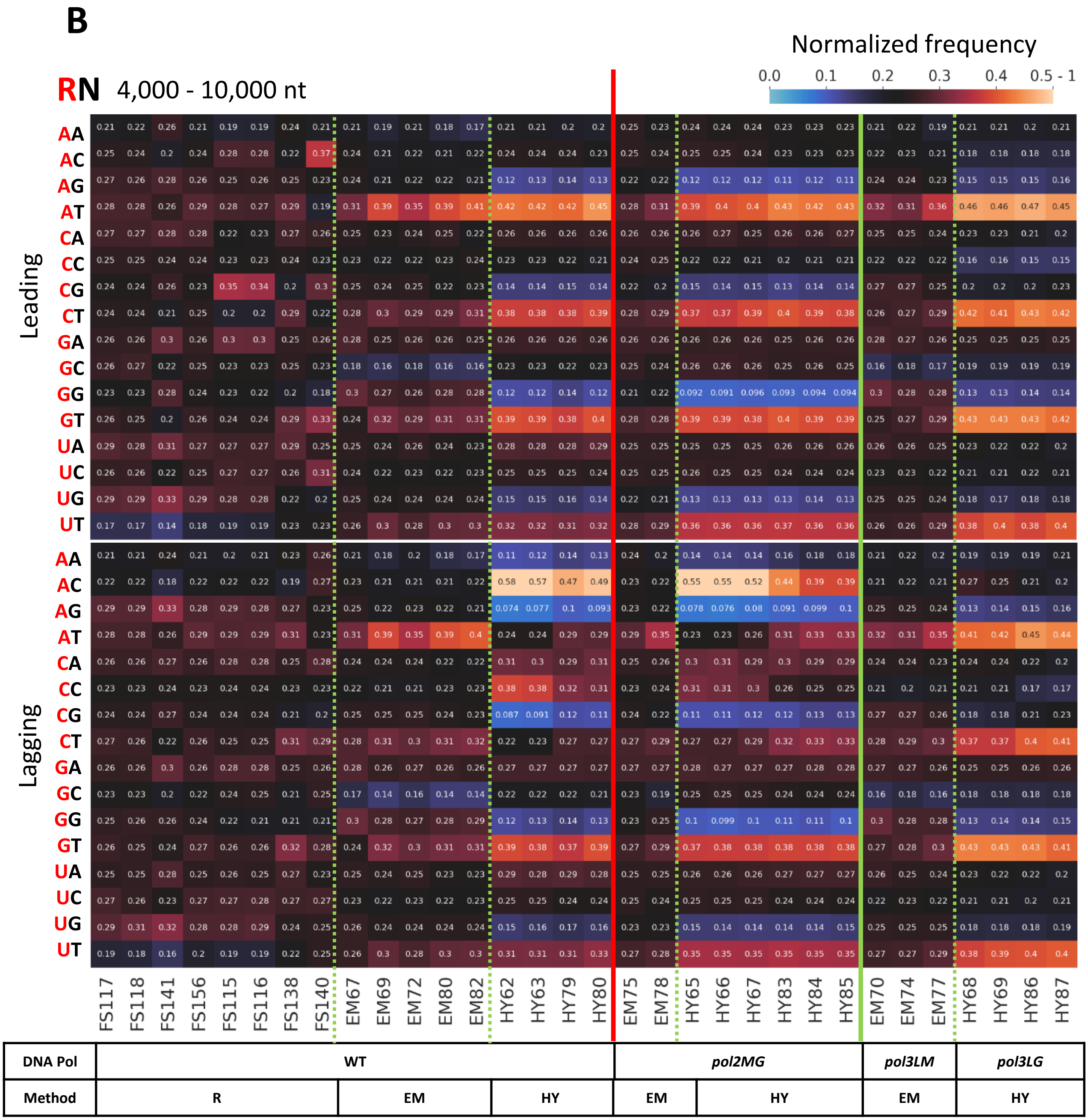

Late-firing ARS

Supplementary Figure 10

C

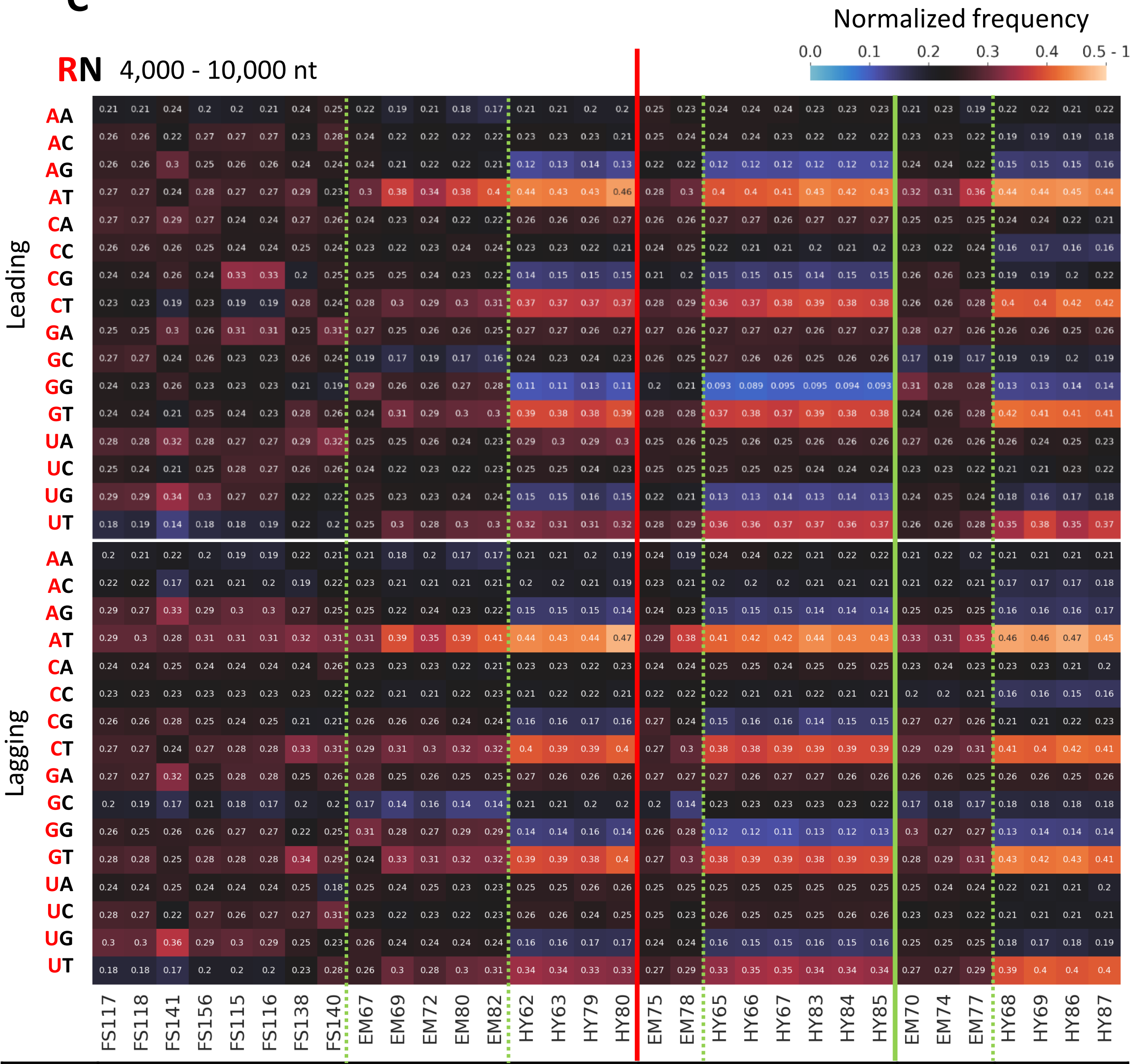

DNA Pol	WT			*pol2MG*		*pol3LM*	*pol3LG*
Method	R	EM	HY	EM	HY	EM	HY

High-efficiency ARS

Supplementary Figure 10

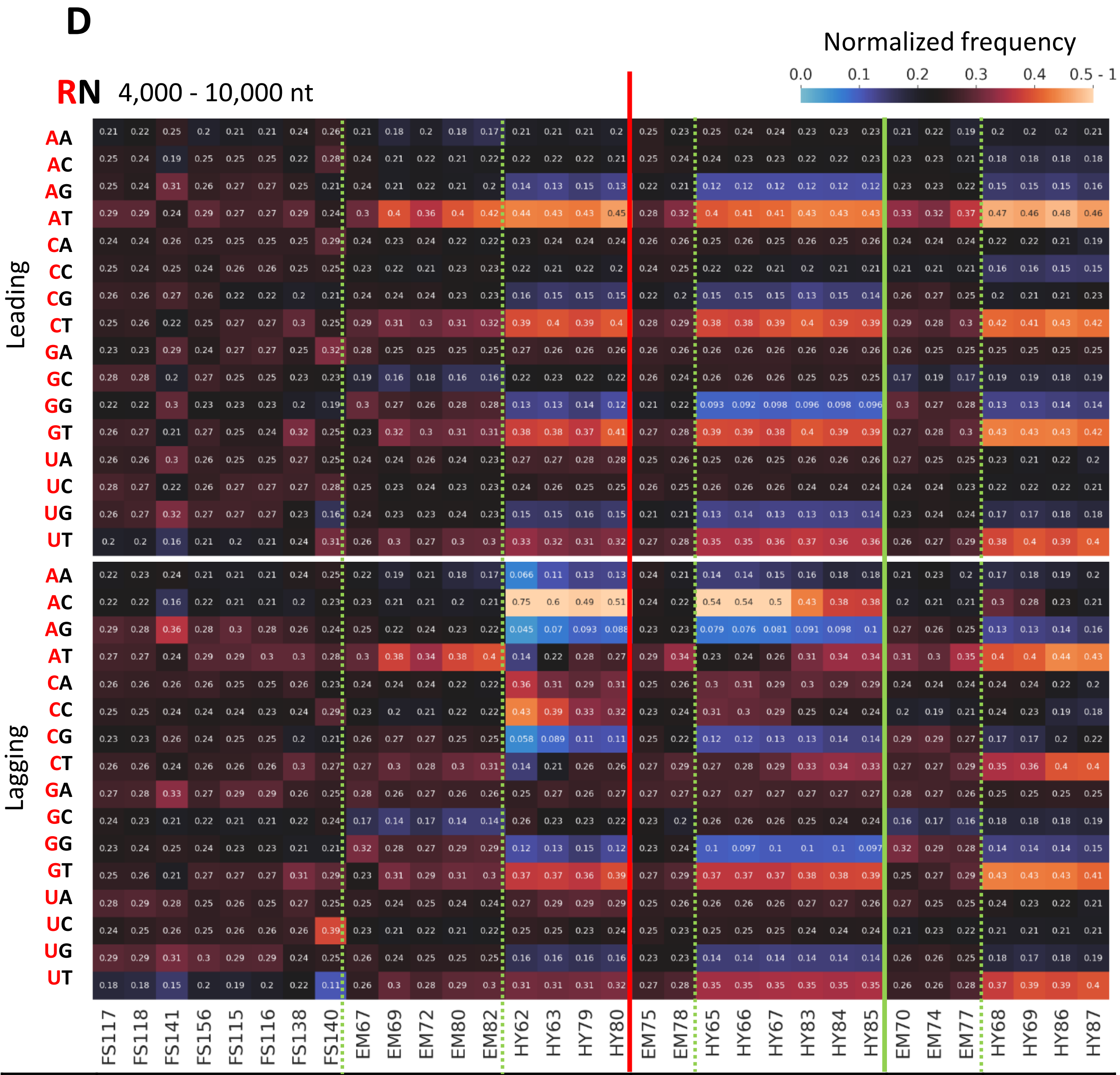

DNA Pol	WT			*pol2MG*		*pol3LM*	*pol3LG*
Method	R	EM	HY	EM	HY	EM	HY

Low-efficiency ARS

Supplementary Figure 10

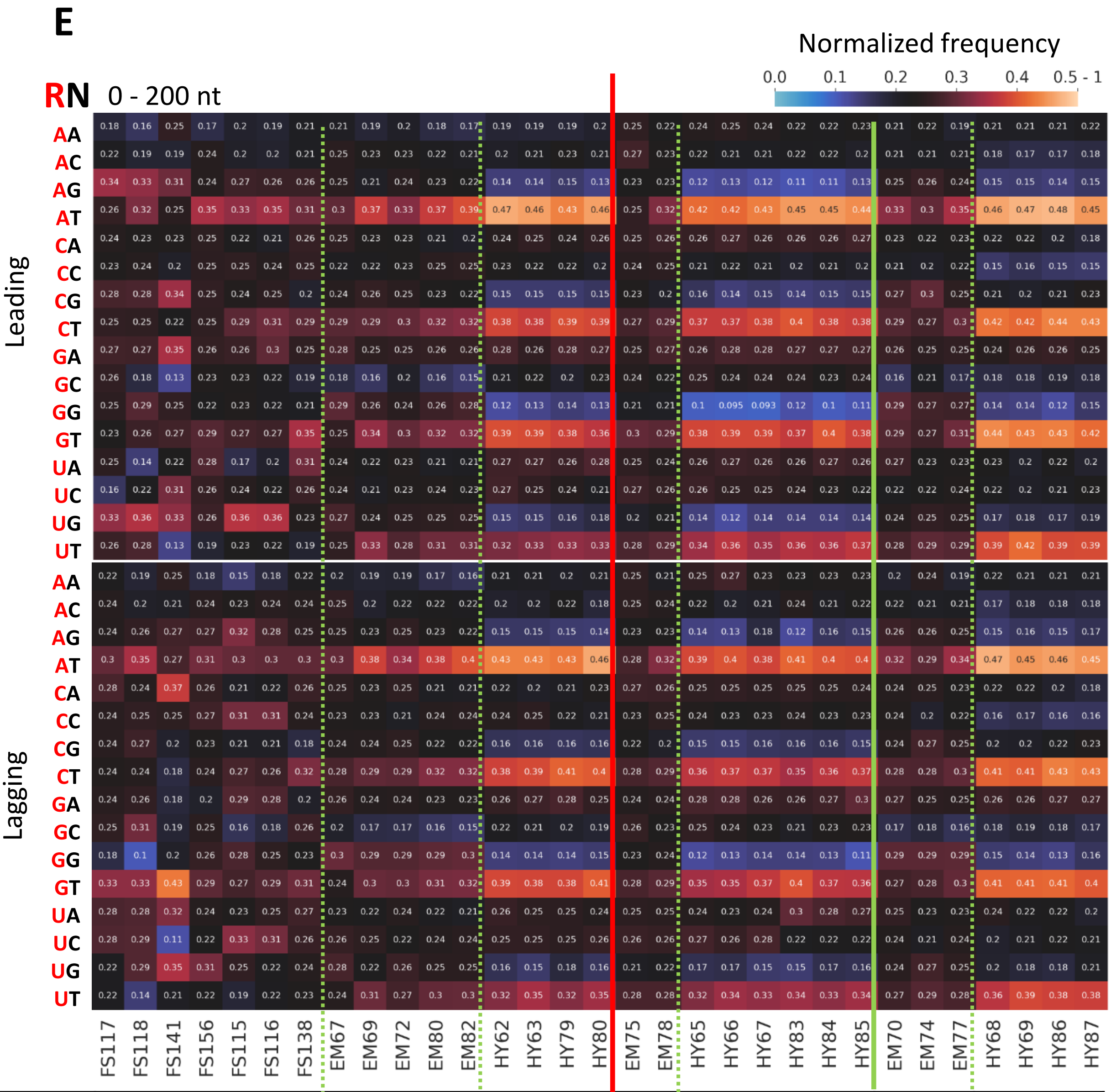

DNA Pol	WT			pol2MG		pol3LM	pol3LG
Method	R	EM	HY	EM	HY	EM	HY

Early-firing ARS

Supplementary Figure 10

F

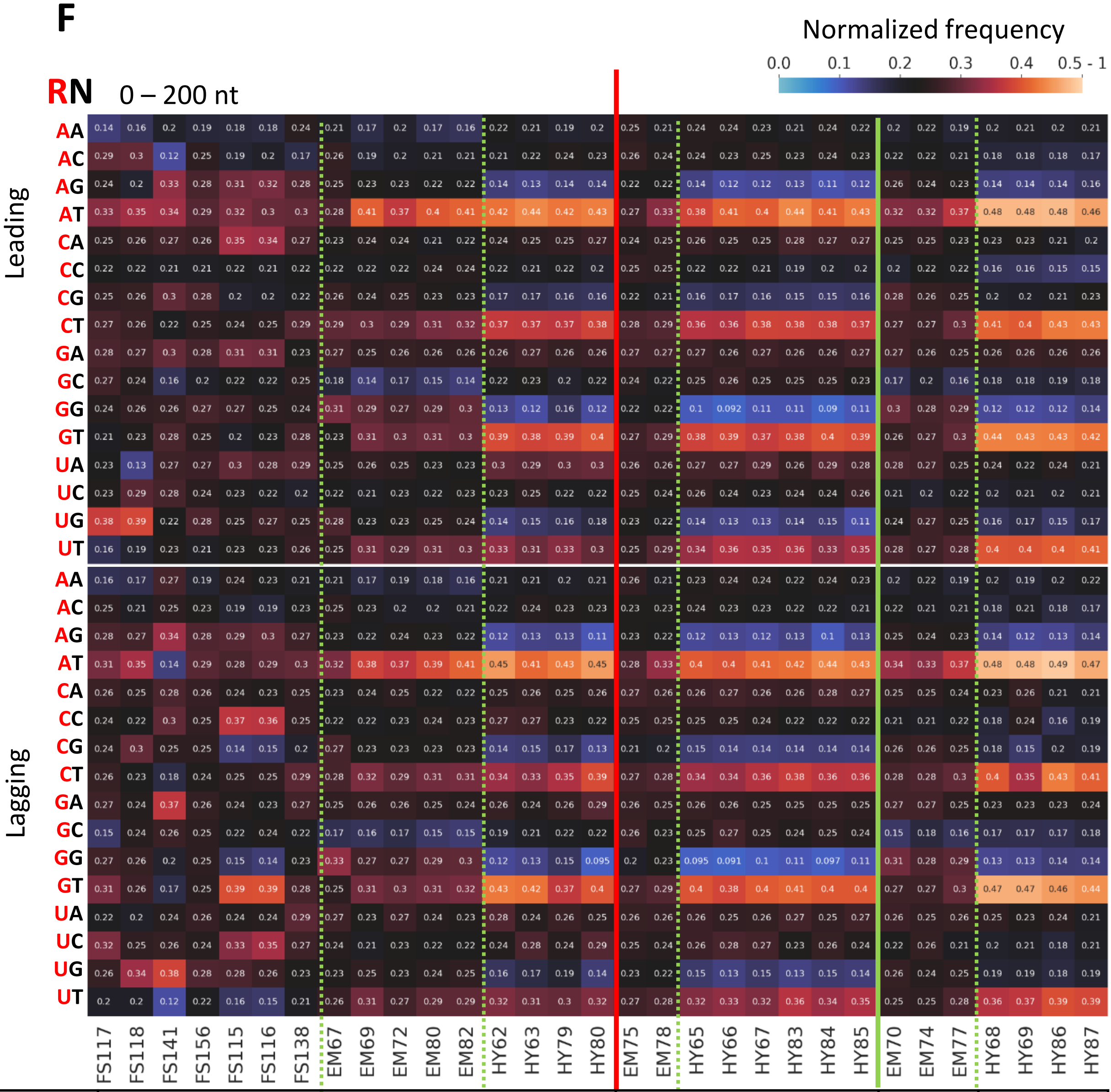

DNA Pol	WT			*pol2MG*		*pol3LM*	*pol3LG*
Method	R	EM	HY	EM	HY	EM	HY

Late-firing ARS

Supplementary Figure 910

G

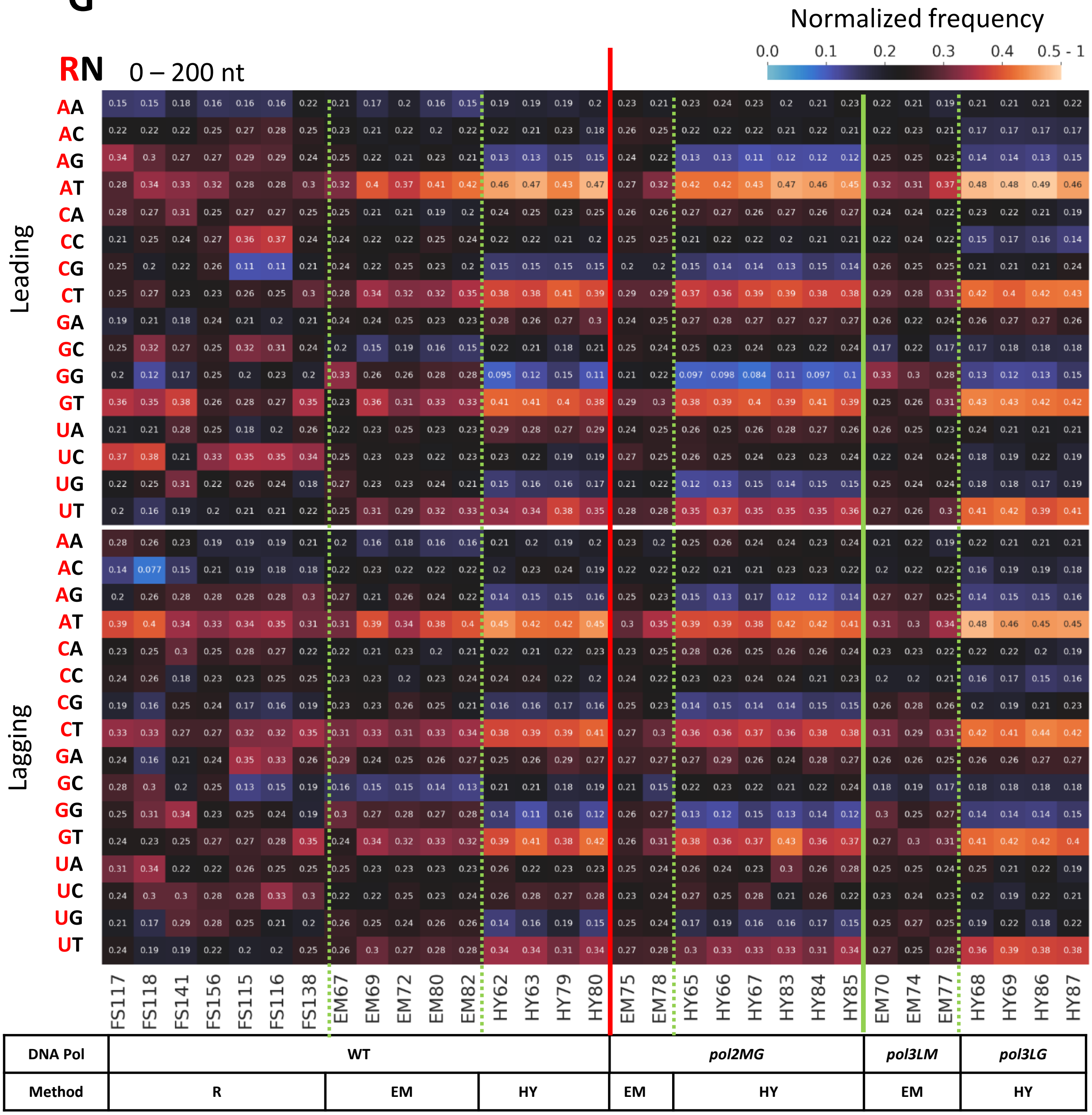

High-efficiency ARS

Supplementary Figure 10

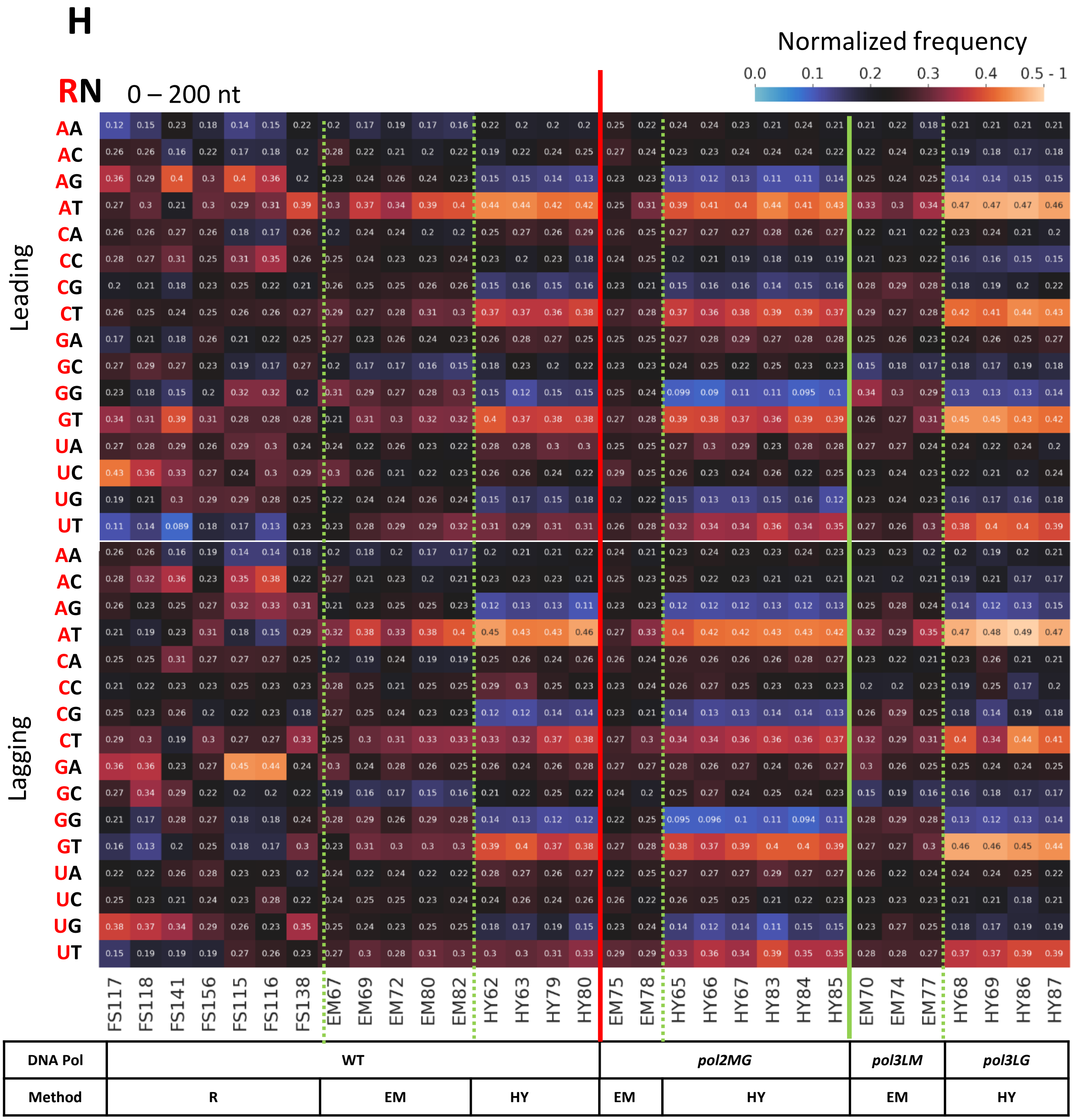

Low-efficiency ARS

Supplementary Figure 10

I

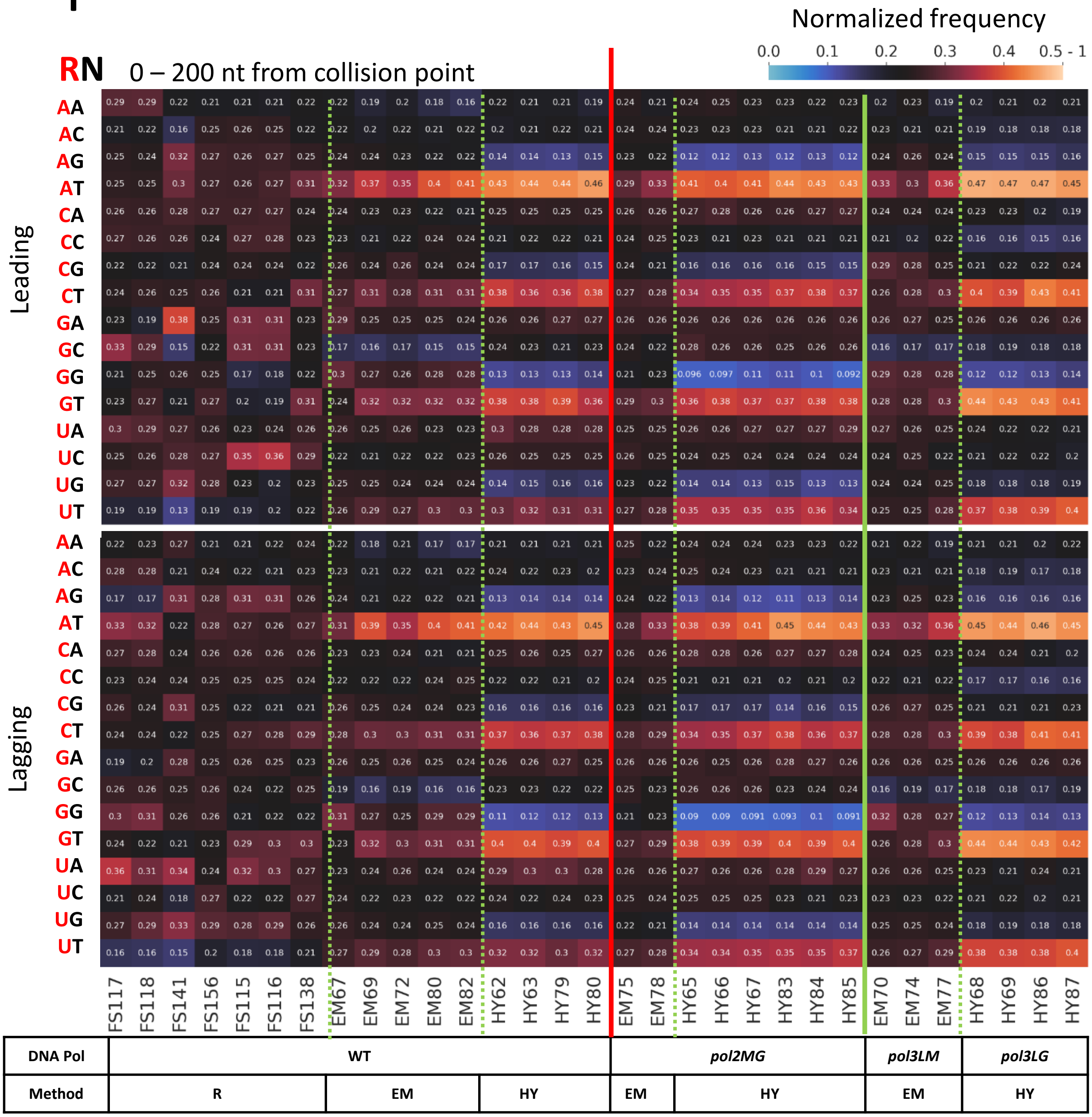

Termination zone

Supplementary Figure 10
